# Skin Penetration Enhancement by Natural Oils for Dihydroquercetin Delivery

**DOI:** 10.3390/molecules22091536

**Published:** 2017-09-12

**Authors:** Vytis Čižinauskas, Nicolas Elie, Alain Brunelle, Vitalis Briedis

**Affiliations:** 1 Department of Clinical Pharmacy, Lithuanian University of Health Sciences, Sukilėlių pr. 13, Kaunas 50166, Lithuania; vitalis.briedis@lsmuni.lt; 2 Institut de Chimie des Substances Naturelles, CNRS UPR 2301, University Paris-Sud, Université Paris-Saclay, Avenue de la Terrasse, Gif-sur-Yvette 91198, France; nicolas.elie@cnrs.fr (N.E.); alain.brunelle@cnrs.fr (A.B.)

**Keywords:** natural oils, skin penetration enhancement, dihydroquercetin, natural oils skin penetration, fatty acids penetration

## Abstract

Natural oils are commonly used in topical pharmaceutical formulations as emulsifiers, stabilizers or solubility enhancers. They are presented as safe and inert components, mainly used for formulation purposes. It is confirmed that natural oils can affect the skin penetration of various substances. Fatty acids are mainly responsible for this effect. Current understanding lacks reliable scientific data on penetration of natural oils into the skin and their skin penetration enhancement potential. In the current study, fatty acid content analysis was used to determine the principal fatty acids in soybean, olive, avocado, sea-buckthorn pulp, raspberry seed and coconut oils. Time of flight secondary ion mass spectrometry bioimaging was used to determine the distribution of these fatty acids in human skin ex vivo after application of the oils. Skin penetration enhancement ratios were determined for a perspective antioxidant compound dihydroquercetin. The results demonstrated skin penetration of fatty acids from all oils tested. Only soybean and olive oils significantly increased the skin distribution of dihydroquercetin and can be used as skin penetration enhancers. However, no correlation can be determined between the fatty acids’ composition and skin penetration enhancement using currently available methodological approaches. This indicates that potential chemical penetration enhancement should be evaluated during formulation of topically applied products containing natural oils.

## 1. Introduction

Natural oils are common ingredients in topical pharmaceutical formulations. They can be categorized into essential and fixed oils [1]. The current study was performed using fixed natural oils. Usually they are presented as “safe agents” and used as emulsifiers, stabilizers or solubility enhancers. Due to favorable technological properties, they can be found in novel nano-scaled dosage forms [2]. Scientific data demonstrate that natural oils can also change the skin’s permeability and be utilized as skin permeability enhancers in topical drug formulations [3].

Natural oils are heterogeneous lipid mixtures composed of triglycerides and minor components—mono- and diglycerides, free fatty acids (FAs), phosphatides, sterols, fatty alcohols, fat-soluble vitamins, and other substances [4]. FAs are the main components in natural oils which can modify the skin barrier. They act by fluidization and disorganization of skin lipids in a reversible manner [5]. Scientific data shows that based on the structure-skin permeability enhancement relationship, unsaturated C16–C18 FAs are more efficient in increasing the permeability of skin barrier if compared to saturated FAs [6,7]. Therefore, natural oils containing high amounts of unsaturated FAs should be expected to demonstrate more significant skin penetration enhancement for drug compounds. FAs’ compositions of olive, avocado, soybean, raspberry seed and sea-buckthorn pulp oils are mainly composed of unsaturated linoleic, oleic, palmitoleic and α-linolenic acids making them promising candidates as skin penetration enhancers [8]. However, existing scientific data provides a controversial view on skin penetration enhancement by natural oils.

Efficient skin penetration enhancement by natural oils was demonstrated for flurbiprofen [3,9]. However, in an in vivo scanning laser microscopy imaging study by Patzelt et al. the possibilities for natural oils to enhance the penetration of curcumin and penetrate deeper regions than the *stratum corneum* were excluded [10], although the method used in the study was specific to curcumin dissolved in oils rather than to the lipid components of natural oils. Also it must be noted that the effect of skin penetration enhancement highly depends on the penetrant molecule [11]. A precise, specific to lipid components and label-free method is needed to evaluate the skin penetration effects of natural oils with certainty. Time of flight secondary mass spectrometry (TOF-SIMS) bioimaging is a label-free surface analysis method suitable for lipid analysis in biological samples. This method provides high spatial resolution and repeatability which was confirmed by numerous studies [12,13,14,15,16], and can also be used for semi-quantitative measurements [17,18]. Our previous studies demonstrated suitability of TOF-SIMS bioimaging for spatial distribution analysis and semi-quantitative evaluation of selected individual FAs in human skin ex vivo [19,20]. In the current study, this method was used to analyze the skin penetration analysis of natural oils.

The overall objective of this study was to evaluate the skin penetration effects of natural oils and their performance as skin penetration enhancers for dihydroquercetin (DHQ). DHQ is a promising compound for treating environment-associated skin conditions. Scientific data shows that DHQ can act as anti-inflammatory [21,22], hypopigmenting [23], anti-tumour [24], mitigating oxidative DNA damage [25] and preventing UV-induced skin carcinogenesis [26] agent. In vivo trials with experimental animals showed positive DHQ effects in treating chemically induced atopic dermatitis-like lesions [27] and chemically induced burns [28,29]. Based on relatively low molecular mass (<600 Da), adequate water/octanol partitioning coefficient in the range of 1–3 and good solubility in oils DHQ can be used for topical drug delivery but low solubility in water (<0.1%) might be a drawback for reaching adequate concentrations in the skin [30]. Currently there is no relevant scientific data on skin penetration properties of DHQ and finding an efficient way for adequate DHQ delivery into the skin is essential for its topical delivery.

## 2. Results

### 2.1. Fatty Acid Composition Analysis of Natural Oils

Fatty acid methyl ester (FAME) analysis of soybean, olive, avocado, sea-buckthorn pulp, raspberry seed and coconut oils was used to evaluate the FAs compositions and select appropriate candidates for the following TOF-SIMS bioimaging analysis. Full FAs composition of each oil is presented in Table 1. Soybean, olive, avocado, sea-buckthorn pulp and raspberry seed oils were mainly composed of different ratios of the same principal FAs: C16:0 (palmitic), C18:0 (stearic), C18:1 (oleic) and C18:2 (linoleic). Raspberry seed and sea-buckthorn pulp oils additionally contained relatively high amounts of C18:3 (linolenic) 28.53% and C16:1 (palmitoleic) 30.54% acids, respectively. Coconut oil was mainly composed of saturated short chain C8:0 (caprylic), C10:0 (capric), C12:0 (lauric), C14:0 (myristic) and lower amounts of C16–C18 fatty acids. Based on the topmost content, occurrence in the oils and potential for skin penetration enhancement C16:0 (palmitic), C18:0 (stearic), C18:1 (oleic) and C18:2 (linoleic) and C16:1 (palmitoleic) acids were selected for TOF-SIMS imaging analysis.

### 2.2. TOF-SIMS Imaging of Skin Samples Treated with Natural Oils

**Figure 1:**
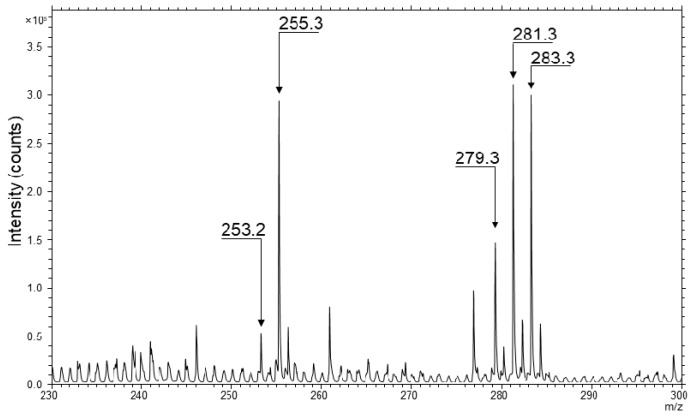
Part of time of flight secondary mass spectrometry (TOF-SIMS) negative ion spectrum of the control skin sample. Marked peaks represent *m*/*z* and intensities of carboxylate ions [M − H]^−^ for selected FA in the skin spectrum: palmitoleic acid (C_16_H_29_O_2_^−^) at *m*/*z* 253.2, palmitic acid (C_16_H_31_O_2_^−^) at *m*/*z* 255.3, linoleic acid (C_18_H_31_O_2_^−^) at *m*/*z* 279.3, oleic acid (C_18_H_33_O_2_^−^) at *m*/*z* 281.3 and stearic acid (C_18_H_35_O_2_^−^) at *m*/*z* 283.3.

TOF-SIMS spectrum was obtained from the control skin samples and presented in Figure 1. Ions of selected fatty acids were determined based on their localization in the skin samples and *m*/*z* values. In addition, cholesteryl sulfate (*m*/*z* 465.3) ion was selected to demonstrate the integrity of each sample because of its predominant distribution in epidermal skin layer of the control samples. High mass resolution was determined for selected ions, M/ΔM = 8000 (full width at half maximum, FWHM).

Pure olive, soybean, coconut, avocado, sea-buckthorn pulp and raspberry seed oils were applied on human skin ex vivo for 6 h to evaluate the effects of natural oils on FAs distribution in the skin. TOF-SIMS bioimaging was performed and C16:0 (palmitic), C18:0 (stearic), C18:1 (oleic), C18:2 (linoleic) and C16:1 (palmitoleic) FAs content changes were evaluated in the samples. TOF-SIMS images obtained allowed to analysis of a skin area representing not less than 1.2 mm depth from the skin surface and 0.5 mm width. This area covers full epidermis and partial dermis (full papillary and upper part of reticular dermis layers) layers of the skin. Imaging area was carefully selected to avoid trans-appendageal transdermal delivery routes containing high amounts of lipids, therefore only the trans-epidermal delivery route was analyzed.

TOF-SIMS images represent the spatial localization of linoleic acid, oleic acid, palmitoleic acid, palmitic acid and stearic acid [M − H]^−^ carboxylate ions corresponding to the optical image obtained from the same skin sample. The integrity of epidermal layer in all samples was confirmed by hematoxylin and eosin stained histological sections and cholesteryl sulfate ion distribution, which is mainly localized in the upper part of epidermis (for all TOF-SIMS images, see supplementary materials).

Samples treated with natural oils were compared to the control skin samples—skin without treatment. All skin samples were obtained from the same patient to avoid inter-individual variability. The control sample data was taken from our previously published research, since the skin samples were obtained from the same patient and skin area [19]. Control sample data was reprocessed along with the data of skin samples treated with natural oils. All experimental procedures for the samples treated with natural oils were performed in the same manner as for the control samples.

**Figure 2:**
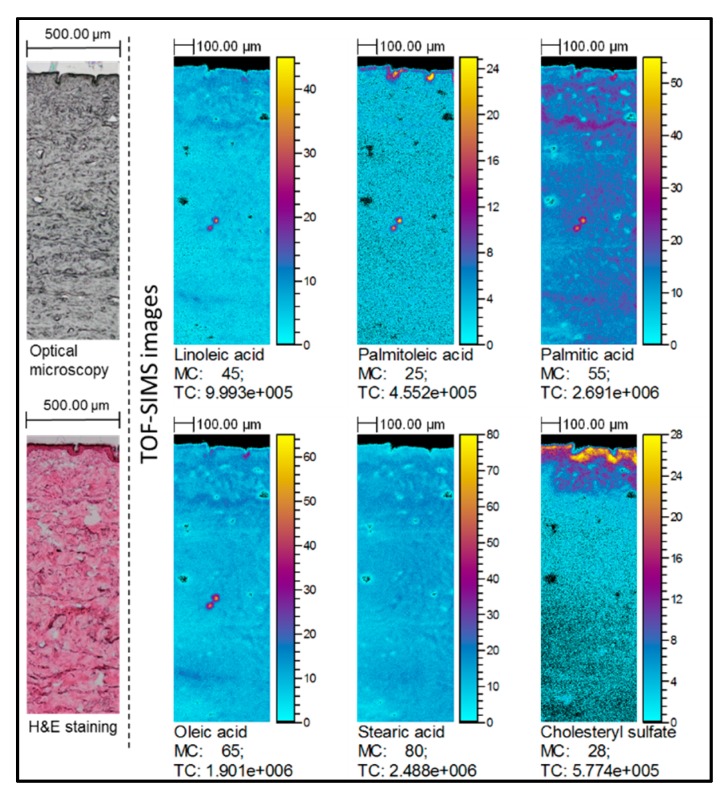
TOF-SIMS images of the skin treated with sea-buckthorn oil: spatial localization of selected ions compared to the optical image. TOF-SIMS images (negative ion mode) showing lateral distribution of palmitoleic, palmitic, linoleic, oleic and stearic acid ions in the skin. Field of view was 1.5 × 0.5 mm^2^, 750 × 250 pixels, pixel size 2 × 2 μm^2^, fluence 5 × 10^11^ ions/cm^2^. The amplitude of the color scale corresponds to the maximum number of counts MC and could be read as [0, MC]. TC is the total number of counts recorded for the specified *m/z* (the sum of counts in all pixels).

**Figure 3:**
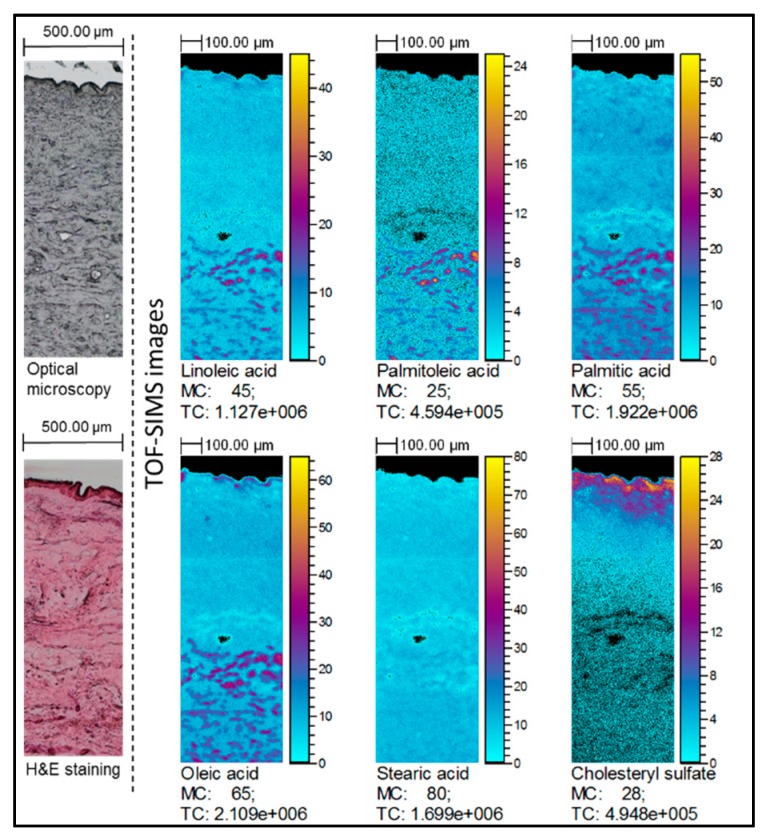
TOF-SIMS images of the skin treated with olive oil: spatial localization of selected ions compared to the optical image. TOF-SIMS images (negative ion mode) showing lateral distribution of palmitoleic, palmitic, linoleic, oleic and stearic acid ions in the skin. Field of view was 1.5 × 0.5 mm^2^, 750 × 250 pixels, pixel size 2 × 2 μm^2^, fluence 5 × 10^11^ ions/cm^2^. The amplitude of the color scale corresponds to the maximum number of counts MC and could be read as [0, MC]. TC is the total number of counts recorded for the specified *m*/*z* (the sum of counts in all pixels).

Notable increase of FAs content was determined in TOF-SIMS images of skin samples treated with natural oils compared to the untreated skin samples. Particularly, increased ion intensities of all selected FAs were determined in the samples treated with sea-buckthorn pulp oil (Figure 2). Increase of linoleic and oleic acids was clearly identified in the skin samples subjected to soybean, olive, avocado and raspberry seed oils. It must be noted that in the samples treated with olive oil (Figure 3) higher intensity regions of co-localized linoleic, oleic, palmitic and palmitoleic acids were determined in dermis layer. However, no visible differences could be determined for the coconut oil-treated samples.

### 2.3. Analysis of Ion Intensity Profiles of the Samples Treated with Natural Oils

Detailed analysis of FAs penetration from natural oils was performed by converting TOF-SIMS ion images to the ion intensity profiles as a function of depth. The profiles show the ion intensity detected in the skin sample from the surface to 1.2 mm depth, and integrated over the entire width of the image (0.5 mm). Average epidermis thickness value (0.06162 ± 0.01781 mm) was measured on HE stained skin sections and used to emphasize the differences between FAs intensities in epidermis and dermis skin layers. Fluctuations of FAs were noted in the ion intensity profiles of natural oils-treated samples, unlike the control skin profiles.

Analysis of ion profiles confirmed the visually observed increase of FAs in the ion intensity images (for all profiles see supplementary materials). Increased content of all FAs analyzed was determined in the samples treated with sea-buckthorn pulp oil. Ion intensity profiles of the samples treated with olive oil demonstrated fluctuating increase of FAs at the upper part of dermis and the end of analysis area. The highest accumulation of linoleic acid was found in samples treated with soybean and raspberry seed oils; oleic acid intensities were highest in the samples treated with avocado, olive and raspberry seed oils, while palmitoleic acid increase was clearly visible only in the epidermis layer of samples treated with sea-buckthorn pulp oil. Coconut oil application notably increased palmitic and stearic acid content thorough all the analysis area and showed a minor accumulation of linoleic acid in epidermis. These observations can be related to the compositions of FAs in natural oils presented in Table 1: soybean and raspberry seed oils contain highest amounts of linoleic acid; olive and avocado oils are mainly composed of oleic acid; sea-buckthorn pulp oil contains the highest percentile ratio of palmitoleic acid; coconut oil contains the lowest amounts of unsaturated FAs, which could alter the skin barrier properties and change the distribution of FAs in the skin.

**Figure 4:**
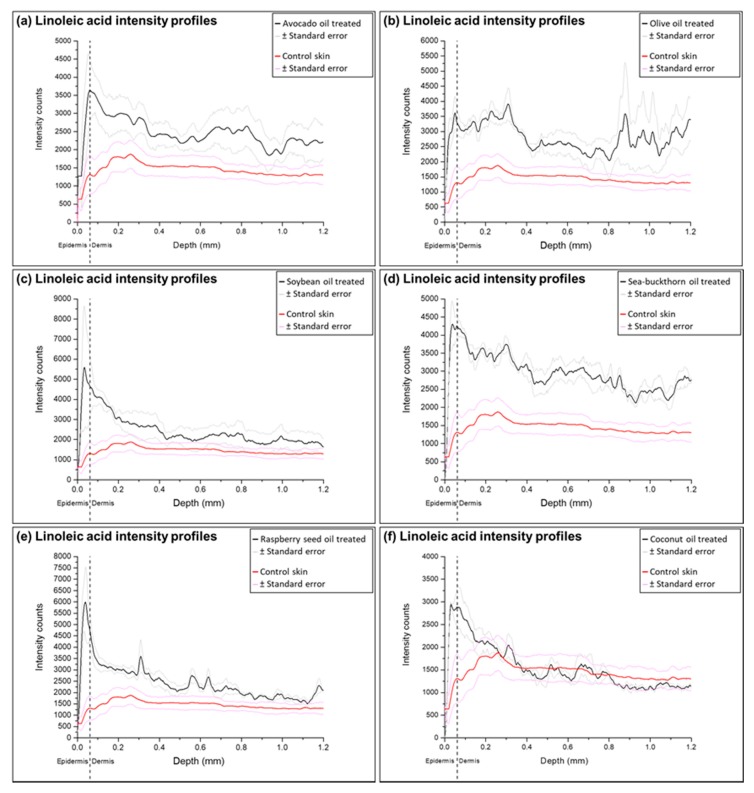
Ion intensity profiles for linoleic acid detected in samples treated with natural oils and compared to the intensity profiles of the control: (**a**) avocado oil; (**b**) olive oil; (**c**) soybean oil; (**d**) sea-buckthorn pulp oil; (**e**) raspberry seed oil; (**f**) coconut oil. The intensity profile of treated samples is shown as a solid black line, and solid red line indicates the control. Similar fluctuations are visible in all FA profiles.

**Figure 5:**
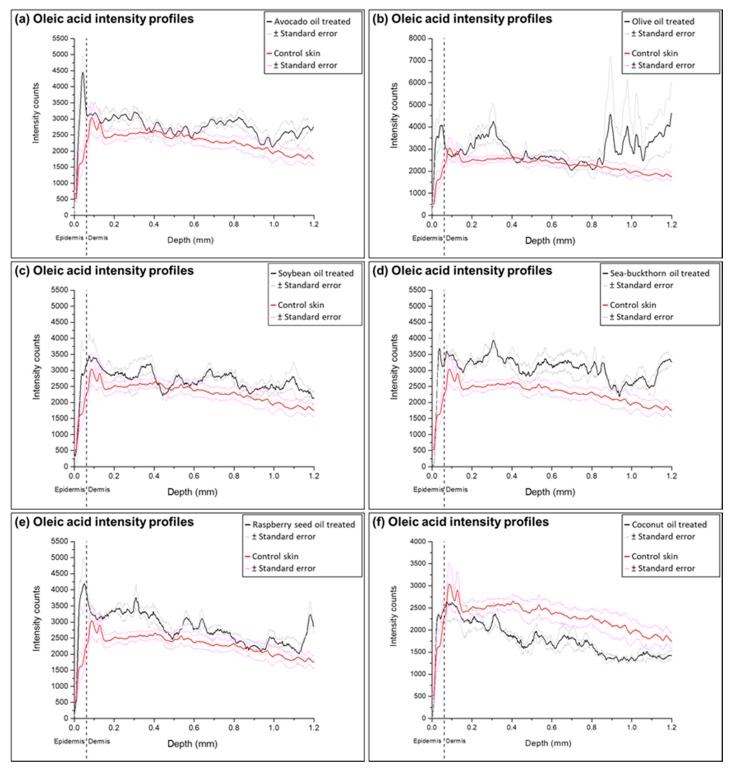
Ion intensity profiles for oleic acid detected in samples treated with natural oils and compared to the intensity profiles of the control: (**a**) avocado oil; (**b**) olive oil; (**c**) soybean oil; (**d**) sea-buckthorn pulp oil; (**e**) raspberry seed oil; (**f**) coconut oil. The intensity profile of treated samples is shown as a solid black line, and solid red line indicates the control. Similar fluctuations are visible in all FA profiles.

Profiles of the samples treated with soybean, raspberry seed and coconut oils, demonstrated the highest increase of linoleic and oleic FAs in epidermis and upper dermis layer. In the samples treated with olive oil, sea-buckthorn pulp oil and avocado oil linoleic and oleic acid content increase is visible through all ion intensity profile area. This suggests that deposition of olive, sea-buckthorn pulp and avocado oils create a smooth fluid region for the transport of linoleic and oleic acids, while they are retained or reaches the concentrations of their saturation in dermis layer of samples treated with soybean, raspberry seed and coconut oils (Figure 4 and Figure 5).

### 2.4. Semi-Quantitative Analysis of Fatty Acid Content Changes

Ion intensity profiles were integrated for a semi-quantitative analysis of FAs content in skin samples treated with natural oils. The profiles were subdivided into two areas corresponding to epidermis (0–0.06162 mm) and dermis (0.06162–1.2 mm) based on the measured average epidermis thickness value. Area under the intensity profile was integrated and divided by the depth of the sample obtaining a unit corresponding to intensity counts per 1 mm of depth in sample. Epidermis and dermis values were used to perform a semi-quantitative analysis by the means of changes compared to the control samples. Statistical comparisons were performed using a non-parametric Mann-Whitney test at the level of significance *p* < 0.05.

**Figure 6:**
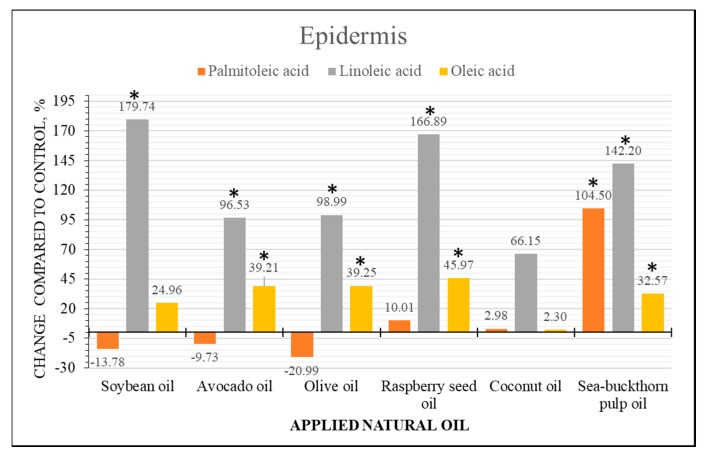
Comparison of FAs content changes in epidermis of human skin ex vivo samples treated with natural oils. The bars represent the percentile changes of intensities detected in the epidermis of natural oils treated samples and compared to the control. Statistically significant changes are marked * *p* < 0.05.

**Figure 7:**
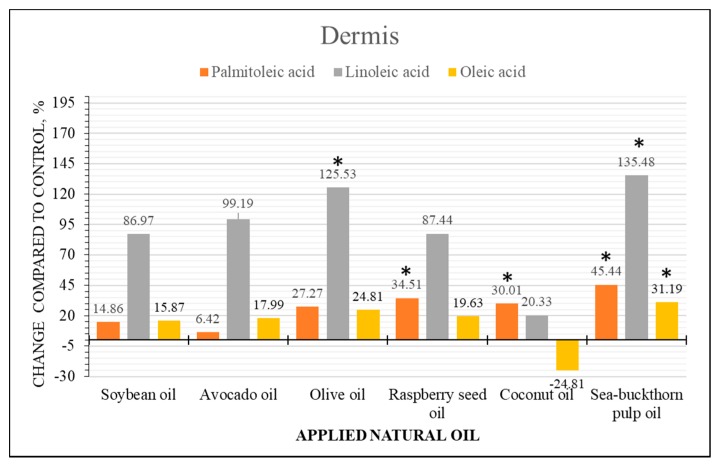
Comparison of FAs content changes in dermis of human skin ex vivo samples treated with natural oils. The bars represent the percentile changes of intensities detected in the dermis of natural oils treated samples and compared to the control. Statistically significant changes are marked * *p* < 0.05.

Results demonstrated that skin treatment with natural oils induced statistically significant FAs distribution changes in both layers of the skin. Distribution changes of linoleic, oleic and palmitoleic acid in epidermis and dermis layers are presented in Figure 6 and Figure 7, respectively. Soybean and raspberry seed oils demonstrated highest increase of linoleic acid in the epidermis layer which could be related to highest linoleic acid content in these oils. It must be noted that migration of linoleic acid is evident in all samples. Oleic acid increase was highest in raspberry seed oil treated samples but showed no statistical difference when treated with other oils. Palmitoleic acid content decrease in epidermis and increase in dermis of the samples treated with soybean, avocado and olive oils suggest migration of endogenous palmitoleic acid. Evident palmitoleic acid increase was determined in both skin layers of the samples treated with sea-buckthorn pulp oil, which contains highest amount of palmitoleic acid. Coconut oil contains low levels of unsaturated FAs and did not induce significant distribution changes of unsaturated FAs in epidermis, although a slight increase of palmitoleic acid content in dermis was determined, and which seems to migrate in the presence of other FAs.

The main distribution changes of saturated palmitic and stearic FAs content were detected in skin samples treated with sea-buckthorn pulp, raspberry seed and coconut oils (see supplementary materials). Despite the FAs composition differences in these oils, the increase of stearic and palmitic acids in both skin layers was similar. Statistical comparison between all skin treatments with natural oils demonstrated that there is no difference between the total effects of soybean, olive and avocado oils (*p* > 0.05). All these results suggest that FAs from natural oils migrate into both skin layers and it is difficult to determine the relationship between the penetration of single FA and FAs composition in the natural oils.

### 2.5. Effect of Natural Oils on Dihydroquercetin Skin Distribution

The skin penetration enhancement effect of natural oils was evaluated for DHQ delivery into the skin layers. Low DHQ penetration levels were expected due to its limited solubility in water. For this reason, in vitro human skin penetration experiments were performed for 24 h using 0.5% (*w*/*w*) DHQ mixed with 10% (*w*/*w*) natural oil solutions in PEG 400. The amount of DHQ (μg/cm^2^, n = 5) penetrating into epidermis and dermis from control solution and natural oil solutions was quantified by UPLC-UV and the results are presented in Table 2 The enhancing effect of natural oils on DHQ penetration into 1 cm^2^ of epidermis and dermis skin layers was expressed as the enhancing ratio (ER) and calculated using the following equation (SL represents a particular skin layer):

(1) $$ER=\frac{\mathrm{DHQ}\;\mathrm{flux}\;\mathrm{into}\;\mathrm{SL}\;\mathrm{from}\;\mathrm{formulation}\;\mathrm{with}\;\mathrm{natural}\;\mathrm{oils}\;(\mu g/{\mathrm{cm}}^{2})}{\mathrm{DHQ}\;\mathrm{flux}\;\mathrm{into}\;\mathrm{SL}\;\mathrm{from}\;\mathrm{control}\;\mathrm{formulation}\;(\mu g/{\mathrm{cm}}^{2})}$$

ANOVA revealed that DHQ flux into both skin layers was significantly greater (*p* < 0.05) in the presence of soybean oil. Olive oil enhanced DHQ penetration into the dermis layer (*p* < 0.05), while avocado, sea-buckthorn pulp, raspberry seed and coconut oils did not significantly increase the flux of DHQ.

## 3. Discussion

The aim of this study was to evaluate the skin penetration of natural oils and their potential application as skin penetration enhancers for DHQ delivery. This is the first experimental study demonstrating penetration of FAs from natural oils into human skin ex vivo. In the current research, we found that the application of olive, avocado, soybean, sea-buckthorn pulp, coconut and soybean oils increases the content of FAs in the ex vivo skin layers. Moreover, soybean and olive oils were determined to be effective skin penetration enhancers for DHQ delivery.

Natural oils are complex lipid mixtures containing various ratios of saturated and unsaturated FAs [8]. There is no relevant scientific data describing the interaction between FAs mixtures and the human skin barrier, therefore knowledge about individual FAs can be used to predict their skin penetration enhancement effects. Lipid protein partitioning theory explains interaction between individual FAs and the skin barrier [31]. According to this theory, FAs containing C18 chain and at least one double bond have the potential to form a “kink” structure and can disrupt the ordered skin lipids. Oleic acid was used as a model FA to prove the theory, since it is a C18 FA and it contains a *cis* double bond at C9 position. [5,31]. H. Tanojo et al. evaluated FAs structure differences, resulting in the perturbation of skin lipids and changes in penetration enhancement, suggesting that C18 FAs with more than one double bond should increase the fluidity of skin lipids even more [32]. This indicates, that C18 linoleic acid containing two unsaturated double bonds should disturb the skin lipids more than oleic acid. Alternatively, C16 palmitoleic acid should be less effective in penetration enhancement. This relation was confirmed in the study of diclofenac penetration enhancement by various FAs [33]. Our recent study demonstrated that the application of linoleic acid on the skin induces the redistribution of endogenous FAs, suggesting that it has more potential to disorder the skin lipids in both skin layers, since no such effect was determined for palmitoleic, oleic, stearic and palmitic acids [19]. However, *s*aturated palmitic and stearic FAs have limited abilities to enhance the skin penetration due to their straight chain structures and high melting points, which were described in a study by Ibrahim et al. [34].

The abovementioned FAs are present in natural oils which were tested in the current study. TOF-SIMS imaging analysis demonstrated significant FAs distribution changes in the skin subjected to the application of natural oils. Based on the effects of individual FAs, natural oils containing high amounts of oleic and linoleic FAs were expected to demonstrate higher disordering of the skin lipids and skin penetration enhancement of DHQ. Soybean, olive, avocado, sea-buckthorn pulp, raspberry seed oils contain high amounts and different concentrations of unsaturated C16–C18 FAs. Significant skin penetration enhancement of DHQ was demonstrated by soybean and olive oils. Soybean oil increased the partitioning of DHQ into both skin layers, while the effect of olive oil was only found in the dermis. These effects can be related to the highest concentration of linoleic acid determined in soybean oil and oleic acid in olive oil. TOF-SIMS analysis of soybean-treated samples demonstrated the highest increase of linoleic acid in the epidermis. Oleic acid content in olive oil treated samples was not as high as in samples treated with raspberry seed oil treated. It must be noted that, also a significant increase of linoleic acid content was determined in both skin layers of the samples treated with olive oil. However, raspberry seed oil contains a similar concentration of linoleic acid as soybean oil, and avocado oil has a very close FAs composition to olive oil, but did not show skin penetration enhancement of DHQ. Moreover, raspberry seed oil and sea-buckthorn pulp oil demonstrated the highest increase of total FAs content in the skin. Alternatively, coconut oil demonstrated minor FAs distribution changes and reduced skin penetration of DHQ. Coconut oil is mainly composed of straight chain unsaturated FAs and the lowest skin penetration is expected due to their higher melting points [34]. It must be noted that skin penetration experiments for natural oils were carried out for 6 h while DHQ penetration experiment in the presence of natural oils was carried out for 24 h. The different duration of experiments was employed to evaluate early effects of FAs penetration from natural oils and determine the penetration of DHQ into the skin layers. Low penetration of DHQ was expected, therefore prolonged experiments allowed the detection of DHQ in both skin layers above the quantification limit. FAs from natural oils penetrate into the skin, but there is no direct relation between the concentration of particular FA in natural oil, its increase in the skin layers and skin penetration enhancement for a target molecule.

It must be noted that predominant components in natural oils are triglycerides while FAs are minor constituents along with mono- and diglycerides, phosphatides, sterols, fatty alcohols, fat-soluble vitamins, and other substances [4]. FAs from natural oils interact with the skin as a mixture of skin penetration enhancers and are also affected by the presence of other components. This increases the complexity of natural oils interaction with the skin barrier and becomes difficult to find a correlation between the composition and penetration enhancement using currently available methodological approaches. Moreover, the quality and FA composition of natural oils can be affected by various factors, such as origin, quality of raw materials, preparation methods or storage conditions [35,36,37,38]. Therefore, natural oils used in formulation of topically applied products should be tested for their FAs content and standardized or tested for their potential interaction with the skin. Standardization of natural oils and evaluation of acceptable variability in FAs composition would provide more possibilities for safe application of natural oils in formulation of topically applied products. This requires large scale skin penetration experiments using a range of oil grades from different sources and one of the ways to perform such study would be a high-throughput screening, as it was demonstrated by Karande, Jain and Mitragotri for discovering of optimal skin penetration enhancer for macromolecules [39].

In overall, this study demonstrates that natural oils should not be treated as passive components. TOF-SIMS bioimaging analysis showed that FAs from natural oils penetrate the skin and change FA distribution in the skin layers. However, no correlation can be determined using currently available methodological approaches. This indicates that potential chemical penetration enhancement should be evaluated during formulation of topically applied products containing natural oils. Moreover, such approach can support implementation of quality by design in pharmaceutical development of topically applied products containing natural oils.

## 4. Materials and Methods 

### 4.1. Materials

Dihydroquercetin (DHQ) (purity 98.4%) from *Larix gmelinii* (Rupr.) was obtained from Taxifolia, Ltd (Belgorod, Russia), polyethylene glycol 400 (PEG 400) was purchased from Carl Roth GmbH (Karlsruhe, Germany), olive oil, soybean oil and coconut oil were purchased from Sigma-Aldrich Chemie GmbH (Steinheim, Germany), avocado oil (European pharmacopeia grade) from Farmalabor (Canosa di Puglia, Italy), sea buckthorn pulp oil was obtained from Sanddorn UAB (Klaipeda, Lithuania), raspberry seed oil from Aroma Zone (Cabrieres d’Avignon, France). Sodium azide (NaN_3_) was obtained from POCh (Gliwice, Poland). Ethanol (96.3%) was obtained from Stumbras AB (Kaunas, Lithuania). All other reagents were of analytical grade.

### 4.2. Analysis of Fatty Acid Composition in Natural Oils

Gas chromatography analysis was performed to determine the fatty acid compositions of natural oils. The fatty acids in natural oils were converted in their methyl esters (FAMEs), using 2 M potassium hydroxide solution in methanol. Quantitative determinations of FAMEs were conducted using Shimadzu GC-2010 gas chromatograph (Shimadzu Corporation, Tokyo, Japan) with a flame ionization detector (FID). Biscyanopropyl polysiloxane stationary phase and 0.2 μm thickness capillary column Rt-2560 (Restek, Bellefonte, PA, USA) (100 m × 0.25 mm) was used for FAMEs separation. The injection volume was 2.0 μL, the temperature of the injection port was 240 °C with the split ratio of 1:100. Nitrogen was used as a carrier gas; the temperature program was 160 °C/5 min, 180 °C/13 min, and 230 °C/28 min. The flow rate was 0.79 mL/min. Identification of FAMEs was performed by comparing their retention times with those of reference standards (Supelco FAME Mix, Trans FAME mix K110, and linolenic acid methyl ester isomer mix Sigma Aldrich Chemical Co., St. Louis, MO, USA). The results of FAs were expressed as percentages of total FAMEs.

### 4.3. Human Skin Distribution Experiments

Experiments to evaluate the penetration of DHQ and fatty acids from natural oils into the human skin ex vivo were approved by Kaunas Region Bioethical Committee (corresponding bioethical permission approval number: BE-2-41). Skin samples were obtained with informed consent from female patients (of age 25–40) undergoing elective abdominoplasty in the Department of Plastic and Reconstructive Surgery, Hospital of Lithuanian University of Health Sciences, Kaunas Clinics. Any extraneous subcutaneous fat was removed from the dermal surface. The skin was frozen and stored at −20 °C for not longer than 6 months before use.

In vitro skin penetration experiments were carried out using Bronaugh-type flow-through diffusion cells (diffusion area 0.64 cm^2^). The acceptor medium was circulated by a peristaltic pump (Masterflex L/SW, Cole-Parmer Instrument Co., Vernon Hills, Illinois, USA). Full-thickness human skin was thawed slowly, pre-cut and mounted on the diffusion cells. The temperature of 37 ± 1 °C was maintained in the cell holder. Skin was equilibrated for 12 h by circulation of the acceptor fluid (0.9% NaCl + 0.005% NaN_3_) underneath the skin. The acceptor fluid was replaced to 4 mL of fresh acceptor fluid. Not less than 0.2 g of donor solutions were applied on the *stratum corneum* side of the skin surface, to ensure full contact of the donor phase with the skin surface and a steady concentration gradient throughout all the experiments.

Pure natural oils were applied to evaluate their effects on the FA distribution in the skin. To evaluate the effect of natural oils for DHQ penetration into the skin donor solutions were prepared by dissolving dihydroquercetin (0.5%, *w*/*w*) in PEG 400 following the addition of natural oil (olive oil, soybean oil, coconut oil, avocado oil, sea buckthorn pulp oil and raspberry seed oil) to comprise 10% (*w*/*w*) of total amount. The solutions were protected from light to avoid possible breakdown of DHQ and stirred overnight to obtain a homogenous dispersion. In the case of coconut oil, heating up to 35 °C was employed while stirring, since it is solid at room temperature. No heating was applied for other natural oil solutions. The solutions were equilibrated to the temperature of 25 °C in a water bath before their deposition on the skin. It must be noted that solution with coconut oil remained clear after equilibration to the room temperature. Dihydroquercetin 0.5% (*w*/*w*) solution in PEG 400 was used as a control.

The acceptor fluid flow rate was 0.6 mL/min. The duration of the skin penetration experiment was 6 h for fatty acid distribution analysis and 24 h for DHQ content analysis. Donor phase was carefully removed and the skin surface was rinsed with ethanol (96.3%) and NaCl solution (0.9%). Residual water from the samples was carefully removed with a tissue wipe. Subsequently, the skin samples were trimmed off, removing the outer residuals. DHQ content analysis was performed directly. Skin samples for fatty acid distribution analysis were immediately frozen using dry ice, wrapped into aluminum foil and stored at −80 °C.

### 4.4. Quantitative Analysis of Dihydroquercetin in the Skin Samples

Epidermis was separated from dermis by applying the dry heat separation method. DHQ was extracted from separated skin layers with methanol and sonication for 30 minutes in sonication bath (USC1200 THD, VWR). DHQ content in skin extracts was quantified at 289 nm using Acquity UPLC H-Class chromatography system (Waters, Milford, MA, USA). Separation of DHQ from endogenous skin matrix compounds was performed on Acquity UPLC BEH C18 (130 Å, 1.7 µm, 2.1 mm × 50 mm, Waters, Milford, MA, USA) column. The mobile phase was delivered in a linear elution gradient from 20 to 58% of solvent A (acetonitrile) in B (0.1% (*v*/*v*) trifluoracetic acid in ultrapure water) for 3 min; the injection volume was 1 µL, flow rate was 0.5 mL/min, and the column temperature was 40 °C. A standard calibration curve was built up by using standard solutions (0.35–28.35 µg/mL). Calibration graphs were plotted according to the linear regression analysis, which gave a correlation coefficient (*R*^2^) of 0.9999. The method was tested and validated for DHQ analysis in methanolic extracts of human skin layers.

### 4.5. Fatty Acids Distribution Analysis in the Skin Samples

TOF-SIMS analysis was used to evaluate the distribution of FAs in the skin samples. Skin specimens were embedded in carboxymethyl cellulose (CMC) (average MW ~250,000), following a standard embedding procedure. Briefly, the skin samples were equilibrated in the cryostat temperature (−25 °C) for approximately 30 minutes and pre-cut to fit the cryomolds. The samples fitted into the cryomolds were covered with a pre-cooled 4% CMC (*w*/*w* %) solution in water, avoiding bubble formation. Cryomolds with CMC covered skin specimens were snap frozen with liquid nitrogen.

CMC embedded skin was cut into 16 μm sections at −25 °C using a CM3050-S cryostat (Leica Microsystèmes SA, Nanterre, France) and immediately deposited on a silicon wafer (2-in.-diameter polished silicon wafers, ACM, Villiers-Saint-Frédéric, France). The cutting direction was parallel to the epidermis, which was selected to avoid sample contamination with *stratum corneum* lipids. The samples were dried under vacuum at a pressure of a few hectopascals for 15 min before the TOF-SIMS analysis. A subsequent skin section of each sample was cut and deposited on a glass slide to measure the thickness of epidermis. A standard histological hematoxylin and eosin (HE) staining method was performed. Optical images of samples for TOF-SIMS analysis and images of HE stained sections were recorded with Olympus BX51 microscope (Olympus, Rungis, France) equipped with lenses ×1.25 to ×50, SC30 color camera, monitored by Olympus Stream Motion 1.9 software and a motorized scanning stage (Marzhauser Wetzlar GmbH, Wetzlar, Germany). Olympus Stream Motion software was used to measure the thickness of epidermis in images of H&E stained samples. 5 points of each sample were measured and results were expressed as average epidermis thickness ± standard deviation.

The experiments were performed using a commercial TOF-SIMS IV time-of-flight mass spectrometer (ION-TOF GmbH, Münster, Germany). The spectrometer was equipped with a liquid metal ion gun (LMIG) filled with bismuth. Bi_3_^+^ cluster ions were used for all experiments. Using this method, primary ions extracted from the source reached the sample surface with a kinetic energy of 25 keV and at an angle of incidence of 45°. Secondary ions were accelerated to an energy of 2 keV, flew through a field-free region, and were reflected with a single stage reflector (effective flight path ~2 m). Then, they were post-accelerated to 10 keV just before hitting the entrance surface of the hybrid detector, which is made of one single micro-channel plate, followed by a scintillator and a photomultiplier. A low-energy electron flood gun was activated between the two primary ions pulses in order to neutralize the sample surface with the minimum damage.

The so-called “high-current bunched mode” has been used for the primary ion column operation during the experiments, providing both a beam focus of 2 μm and a pulse duration of less than 1 ns. Such experimental conditions enabled a high mass resolution, M/ΔM = 5000 (full width at half maximum, FWHM), at *m*/*z* 500. The Bi_3_^+^ primary ion current, measured at 10 kHz with a Faraday cup on the grounded sample holder, is ~0.65 pA in the high-current bunched mode. For images of human skin sections, a large-area analysis (1.5 mm × 0.5 mm) was performed using these same LMIG conditions, and the stage scan. The number of pixels was 750 × 250, with each pixel having a size of 2 × 2 μm^2^. Mass calibrations were conducted internally, and the signals used for initial calibration were H^−^, C^−^, CH^−^, CH_2_^−^, C_2_^−^, C_3_^−^ and C_4_H^−^. The intensities of linoleic, palmitoleic, palmitic, oleic and stearic acids as well as cholesteryl sulfate were measured using the conditions described above in negative ionization mode and expressed as ion intensity images. TOF-SIMS analysis was repeated in triplicate for each natural oil exposed tissue to confirm analytical repeatability and biological reproducibility.

The data was acquired and processed using SurfaceLab Version 6.2 (ION-TOF GmbH, Münster, Germany) software. Further analysis and manipulation of acquired data was carried out using Origin Version 9.0 (OriginLab, Northampton, MA, USA, 2012) software.

### 4.6. Statistical Analysis of Data

The non-parametric Mann-Whitney test was applied to determine the significance of FA content differences determined in the skin by TOF-SIMS analysis. One-way ANOVA (LSD honestly significant difference criterion) test was used to compare DHQ flux values into the skin from control formulations and formulations containing natural oils. The level of significance was determined as *p* < 0.05. Statistical analysis of experimental data was performed using IBM^®^ SPSS^®^ Statisticsfor Windows, Version 21.0 (Armonk, New York, USA: IBM Corp. 2012).

## 5. Conclusions

FAs from natural oils penetrate the human skin ex vivo, but there is no direct relation between the concentration of a particular FA in natural oil, its increase in the skin layers and penetration enhancement for DHQ into the skin layers. Soybean and olive oils efficiently increase the penetration of DHQ into the skin layers and can be used as skin penetration enhancers. However, the composition variability of natural oils based on their origin, quality of raw materials, preparation methods and storage conditions implies that natural oils used in the formulation of topically applied products should be tested for their FA content and standardized or tested for their potential interaction with the skin. A large scale high-throughput screening study should be advisable for the standardization of natural oils.

## Figures and Tables

**Table 1:** Amounts of fatty acids expressed as percentages of total fatty acids methyl esters in natural oils.

Fatty Acid	Soybean Oil	Olive Oil	Avocado Oil	Sea-Buckthorn Pulp Oil	Raspberry Seed Oil	Coconut Oil
C6:0	0.000	0.000	0.000	0.000	0.000	0.285
C8:0	0.000	0.019	0.000	0.016	0.000	5.582
C10:0	0.000	0.000	0.000	0.032	0.000	5.216
C12:0	0.000	0.000	0.000	0.363	0.000	45.943
C14:0	0.067	0.011	0.042	0.402	0.044	17.983
C14:1	0.000	0.000	0.000	0.097	0.000	0.000
C15:0	0.015	0.000	0.000	0.068	0.000	0.010
C16:0	10.478	10.037	8.252	33.294	3.705	9.212
C16:1	0.100	0.859	1.974	30.540	0.122	0.024
C17:0	0.094	0.056	0.041	0.157	0.055	0.039
C17:1	0.052	0.075	0.065	0.102	0.046	0.000
C18:0	3.702	3.475	2.300	0.814	1.812	2.774
C18:1	23.033	79.585	71.820	29.126	14.320	7.754
C18:2	53.909	3.959	14.596	3.547	45.929	4.565
C18:3	7.251	0.580	0.133	0.898	28.530	0.356
C20:0	0.286	0.345	0.133	0.140	0.719	0.098
C20:1	0.354	0.218	0.167	0.124	2.939	0.065
C20:2	0.088	0.010	0.174	0.000	0.276	0.000
C20:3	0.000	0.574	0.029	0.000	0.000	0.000
C20:4	0.036	0.037	0.000	0.000	0.038	0.000
C20:5	0.000	0.000	0.000	0.000	0.112	0.000
C21:0	0.055	0.013	0.094	0.061	0.117	0.000
C22:0	0.323	0.095	0.108	0.032	0.148	0.035
C22:1	0.000	0.000	0.000	0.023	0.423	0.000
C24:0	0.102	0.043	0.044	0.046	0.055	0.034
C24:1	0.016	0.000	0.000	0.019	0.037	0.000
Unidentified	0.041	0.012	0.031	0.105	0.576	0.029

**Table 2:** Dihydroquercetin flux into human epidermis and dermis, and enhancing ratios of natural oils (n = 5).

Donor Phase	DHQ Flux Into Epidermis	ER Epidermis	DHQ Flux Into Dermis	ER Dermis
Control solution	0.109 ± 0.070	-	0.095 ± 0.056	-
Soybean oil (10%)	1.577 ± 1.011	14.451 *	0.457 ± 0.273	4.810 *
Olive oil (10%)	0.104 ± 0.085	0.950	0.352 ± 0.261	3.708 *
Avocado oil (10%)	0.139 ± 0.038	1.276	0.106 ± 0.035	1.116
Sea buckthorn oil (10%)	0.099 ± 0.061	0.909	0.171 ± 0.117	1.802
Raspberry seed oil (10%)	0.036 ± 0.018	0.330	0.147 ± 0.061	1.549
Coconut oil (10%)	0.022 ± 0.011	0.201	0.025 ± 0.016	0.259

Statistically significant changes compared to the control are marked * *p* < 0.05.

## Supplementary Materials

SupplementaryMaterials are available online.
