# Supplementary material for: Skin Penetration Enhancement by Natural Oils for Dihydroquercetin Delivery
Source: Molecules. 2017 Sep 12;22(9):1536. doi: 10.3390/molecules22091536 (PMC6151382; doi:10.3390/molecules22091536)
Supplement: Supplementary file 1 [file molecules-22-01536-s001.pdf]

Supplementary material

- TOF-SIMS analysis images
- Ion intensity profiles of fatty acids as a function of depth
- Semi-quantitative changes of fatty acid content in the samples treated with natural oils

TOF-SIMS analysis of control skin samples

Control skin sample (1)

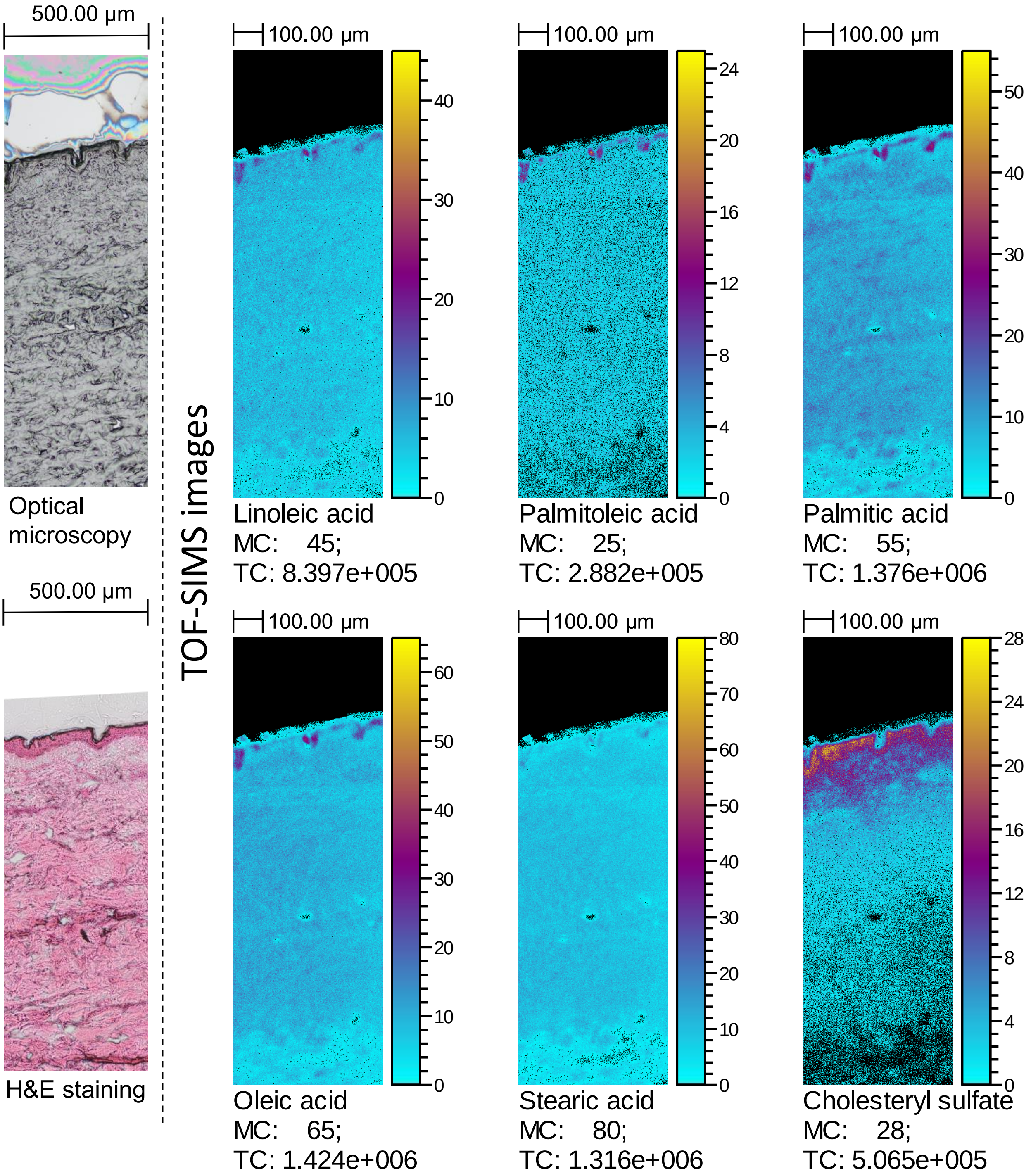

Human skin *ex vivo* control sample.

TOF-SIMS analysis in negative ionisation mode. Each ion image represents spatial localisation of FA analysed in the sample. MC – maximum ion counts detected per image; TC – total ion counts per image.

Optical image represents the sample before TOF-SIMS analysis. H&E staining represents a subsequent section stained with hematoxylin and eosin.

Control skin sample (2)

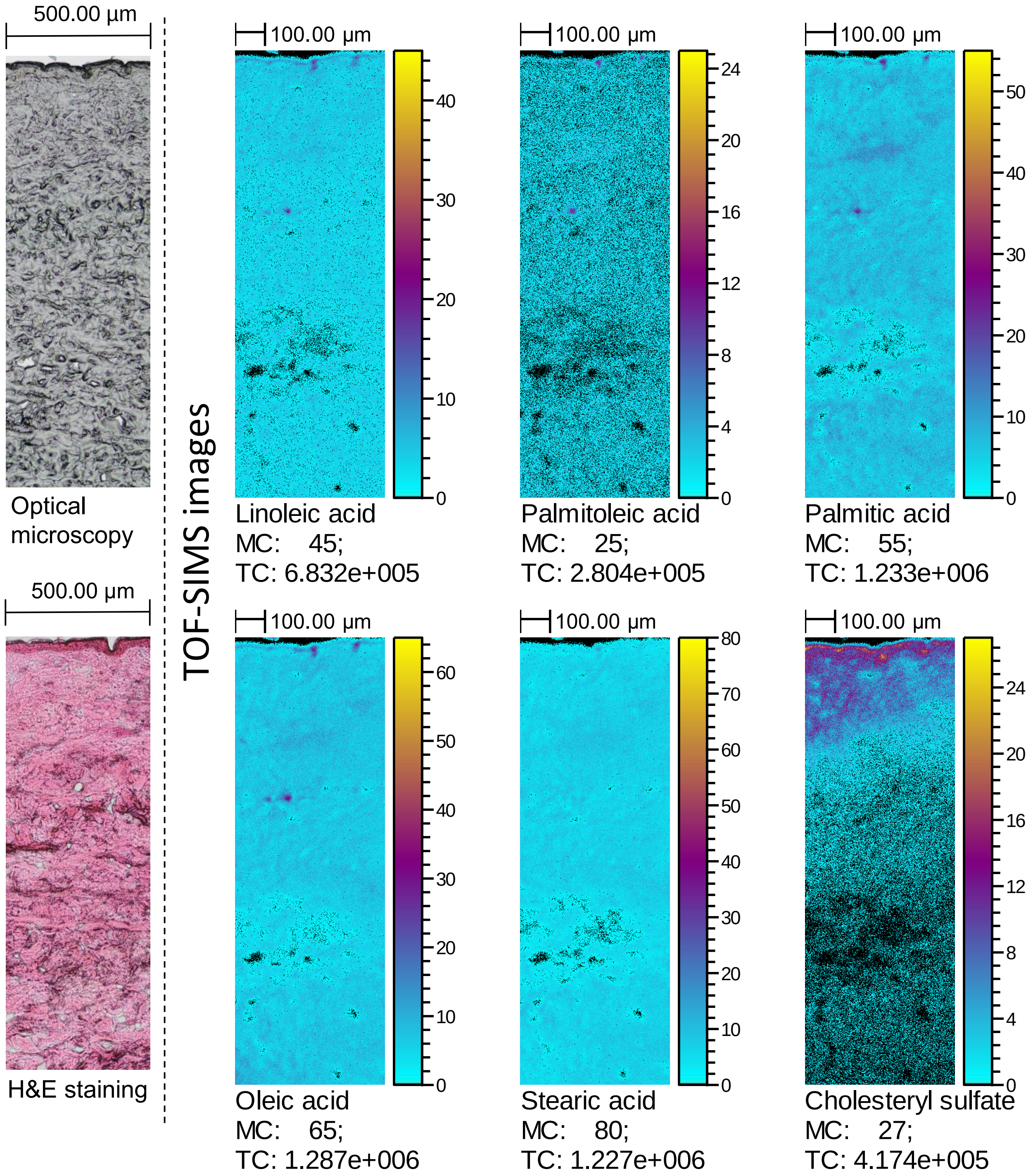

Human skin *ex vivo* control sample.

TOF-SIMS analysis in negative ionisation mode. Each ion image represents spatial localisation of FA analysed in the sample. MC – maximum ion counts detected per image; TC – total ion counts per image.

Optical image represents the sample before TOF-SIMS analysis. H&E staining represents a subsequent section stained with hematoxylin and eosin.

Control skin sample (3)

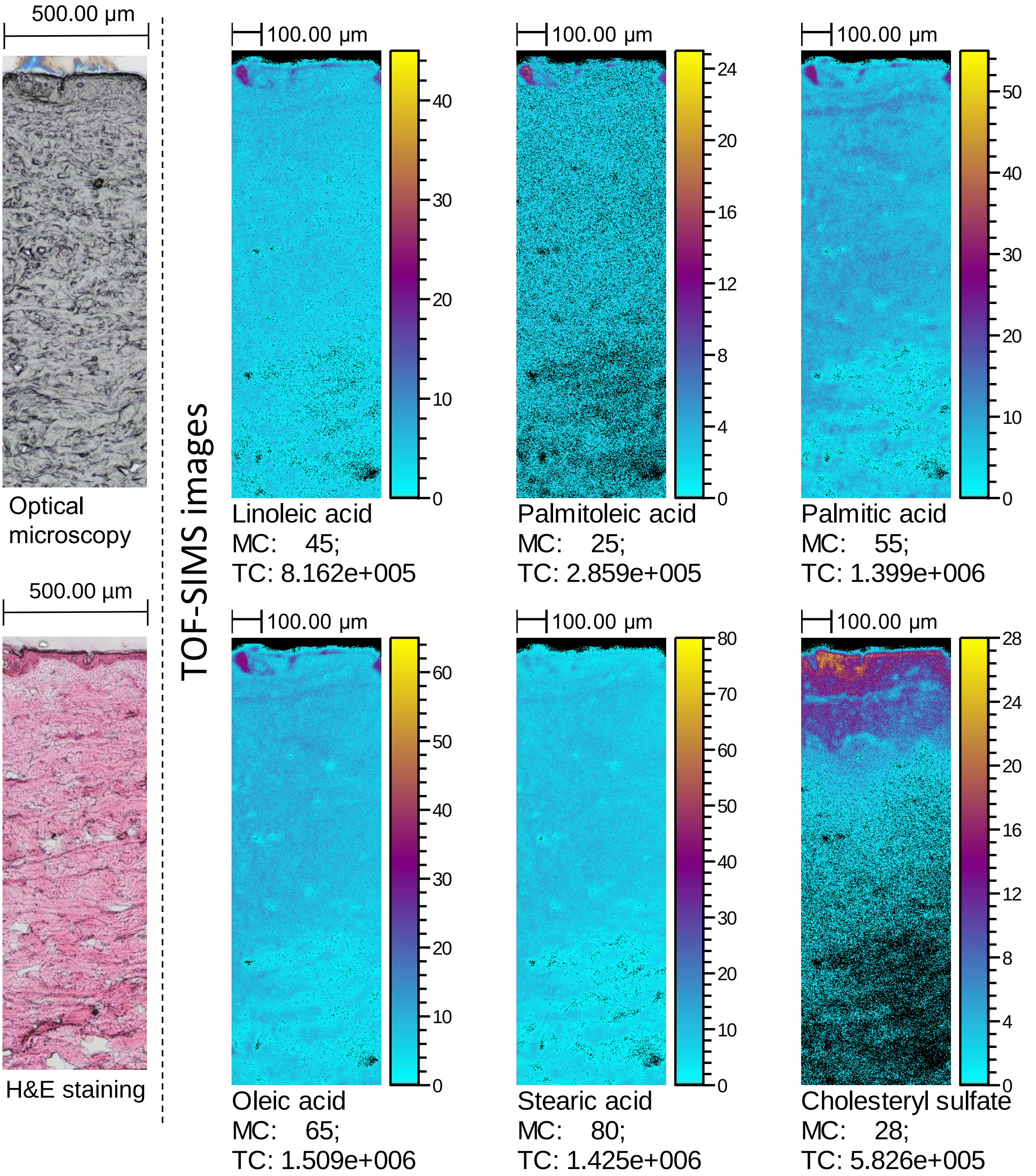

Human skin *ex vivo* control sample.

TOF-SIMS analysis in negative ionisation mode. Each ion image represents spatial localisation of FA analysed in the sample. MC – maximum ion counts detected per image; TC – total ion counts per image.

Optical image represents the sample before TOF-SIMS analysis. H&E staining represents a subsequent section stained with hematoxylin and eosin.

Control skin sample (4)

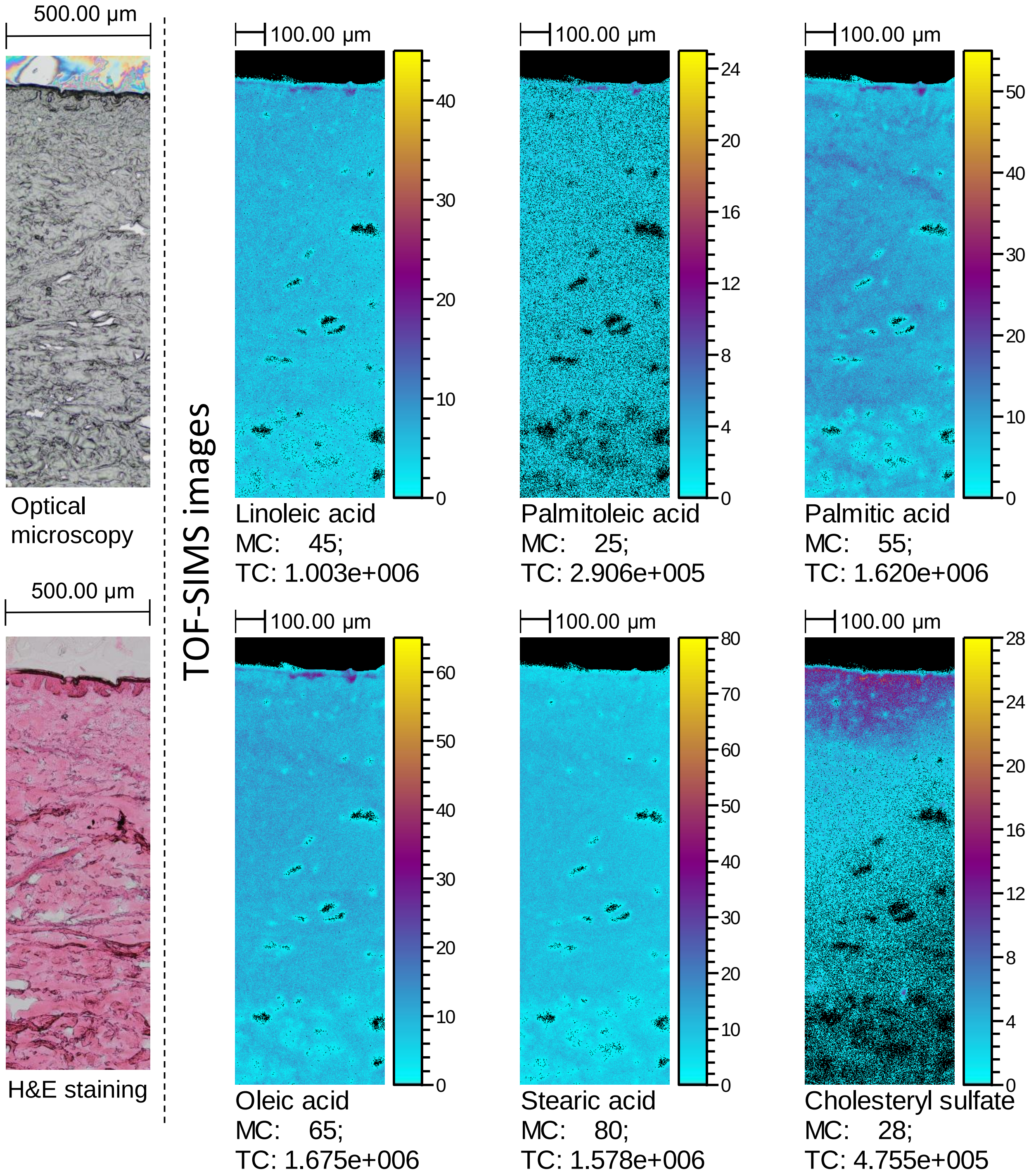

Human skin *ex vivo* control sample.
TOF-SIMS analysis in negative ionisation mode. Each ion image represents spatial localisation of FA analysed in the sample. MC – maximum ion counts detected per image; TC – total ion counts per image.
Optical image represents the sample before TOF-SIMS analysis. H&E staining represents a subsequent section stained with hematoxylin and eosin.

Control skin sample (5)

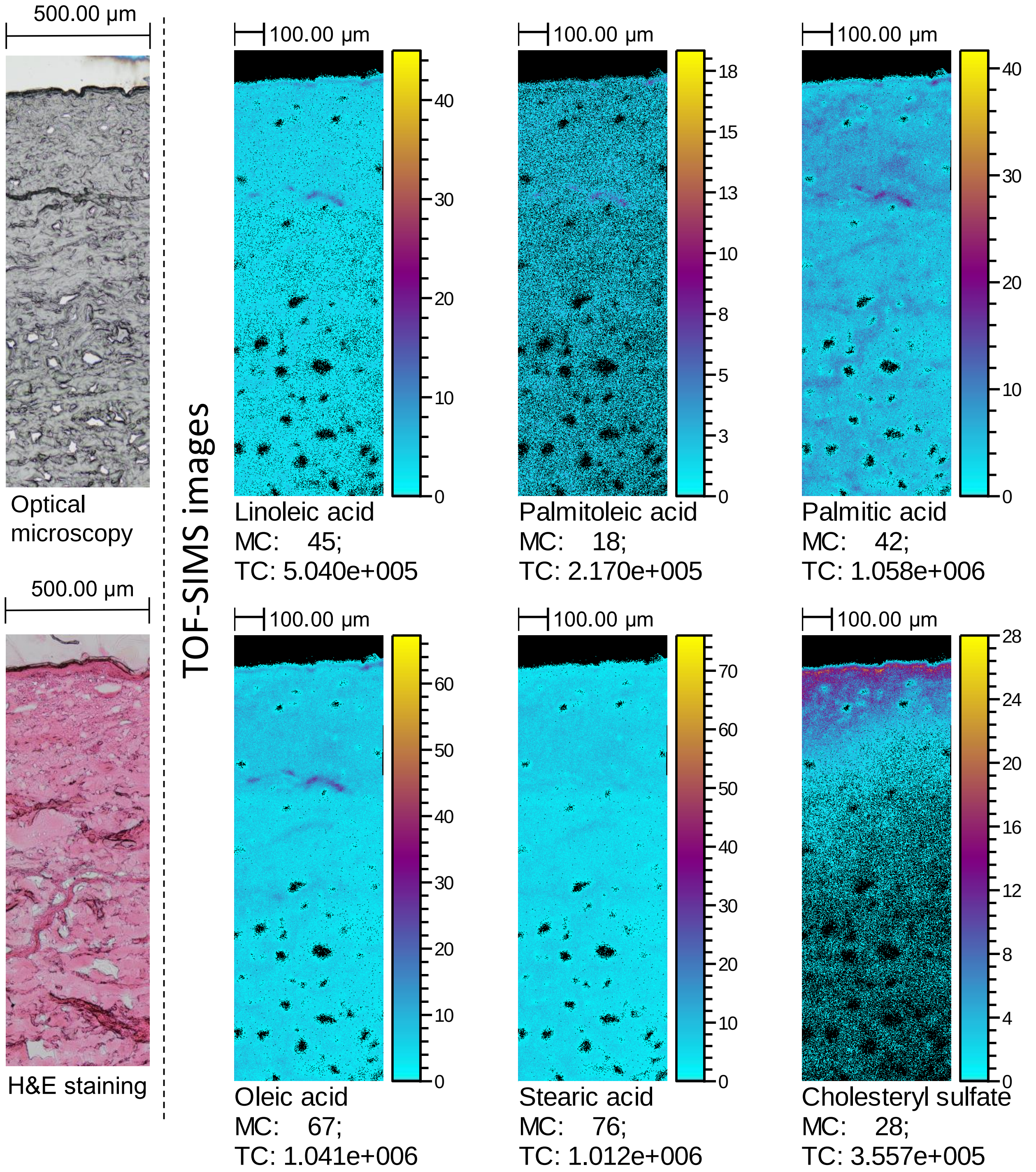

Human skin *ex vivo* control sample.
TOF-SIMS analysis in negative ionisation mode. Each ion image represents spatial localisation of FA analysed in the sample. MC – maximum ion counts detected per image; TC – total ion counts per image.
Optical image represents the sample before TOF-SIMS analysis. H&E staining represents a subsequent section stained with hematoxylin and eosin.

Control skin sample (6)

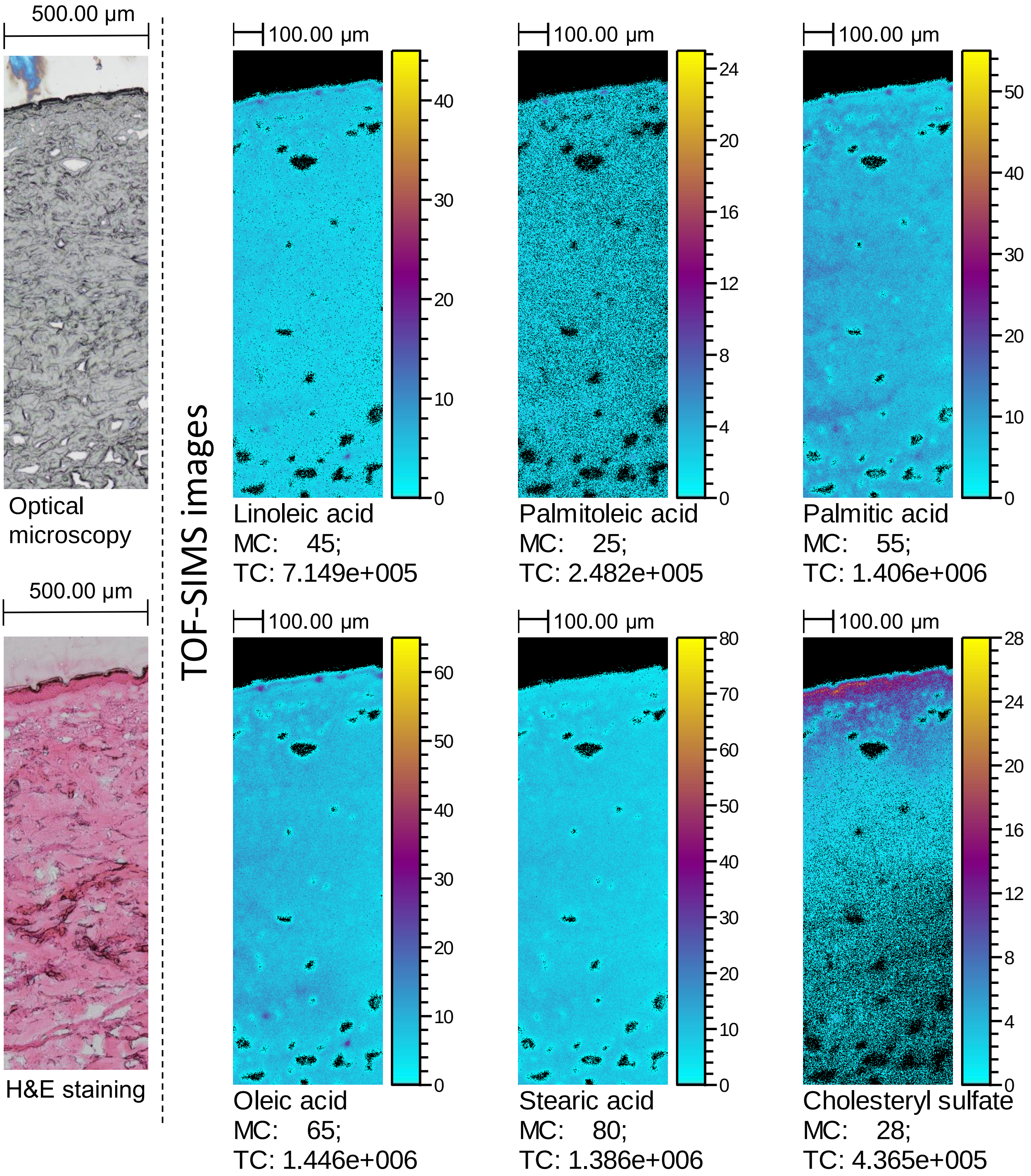

Human skin *ex vivo* control sample.

TOF-SIMS analysis in negative ionisation mode. Each ion image represents spatial localisation of FA analysed in the sample. MC – maximum ion counts detected per image; TC – total ion counts per image.

Optical image represents the sample before TOF-SIMS analysis. H&E staining represents a subsequent section stained with hematoxylin and eosin.

TOF-SIMS analysis of skin samples treated with sea buckthorn pulp oil

Skin sample treated with sea-buckthorn oil (1)

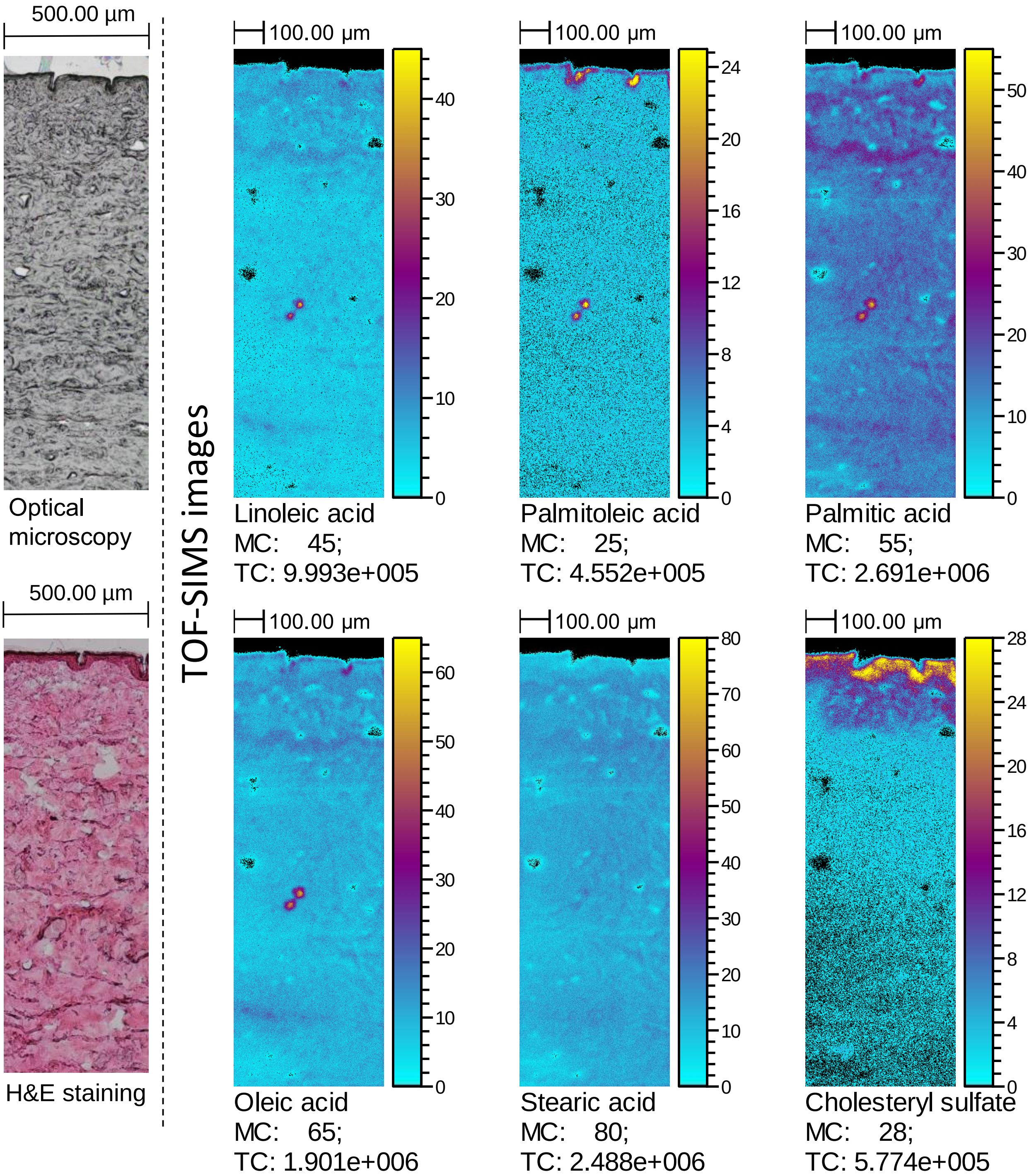

Human skin *ex vivo* sample treated with sea-buckthorn oil.

TOF-SIMS analysis in negative ionisation mode. Each ion image represents spatial localisation of FA analysed in the sample. MC – maximum ion counts detected per image; TC – total ion counts per image.

Optical image represents the sample before TOF-SIMS analysis. H&E staining represents a subsequent section stained with hematoxylin and eosin.

Skin sample treated with sea-buckthorn oil (2)

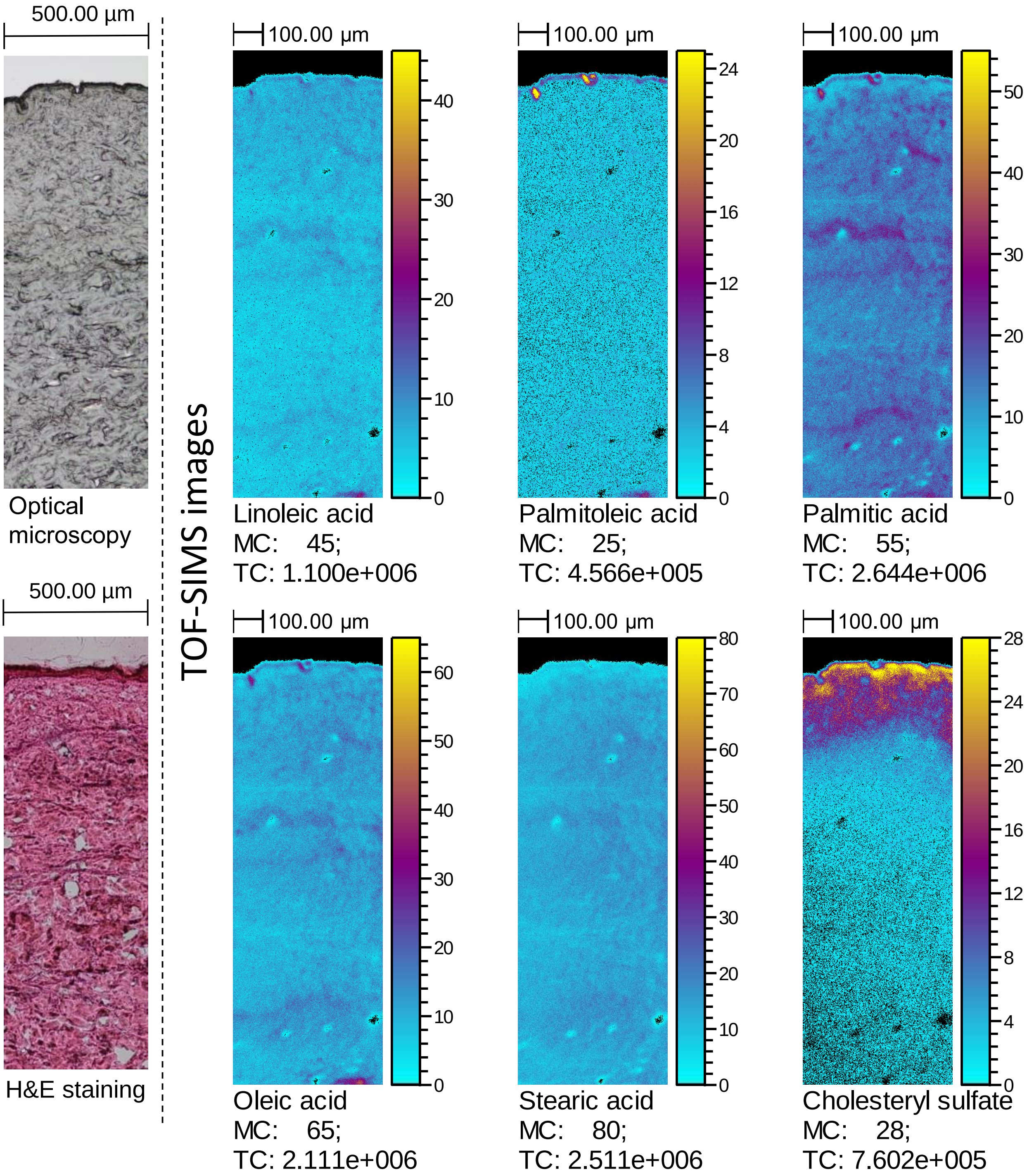

Human skin *ex vivo* sample treated with sea-buckthorn oil.

TOF-SIMS analysis in negative ionisation mode. Each ion image represents spatial localisation of FA analysed in the sample. MC – maximum ion counts detected per image; TC – total ion counts per image.

Optical image represents the sample before TOF-SIMS analysis. H&E staining represents a subsequent section stained with hematoxylin and eosin.

Skin sample treated with sea-buckthorn oil (3)

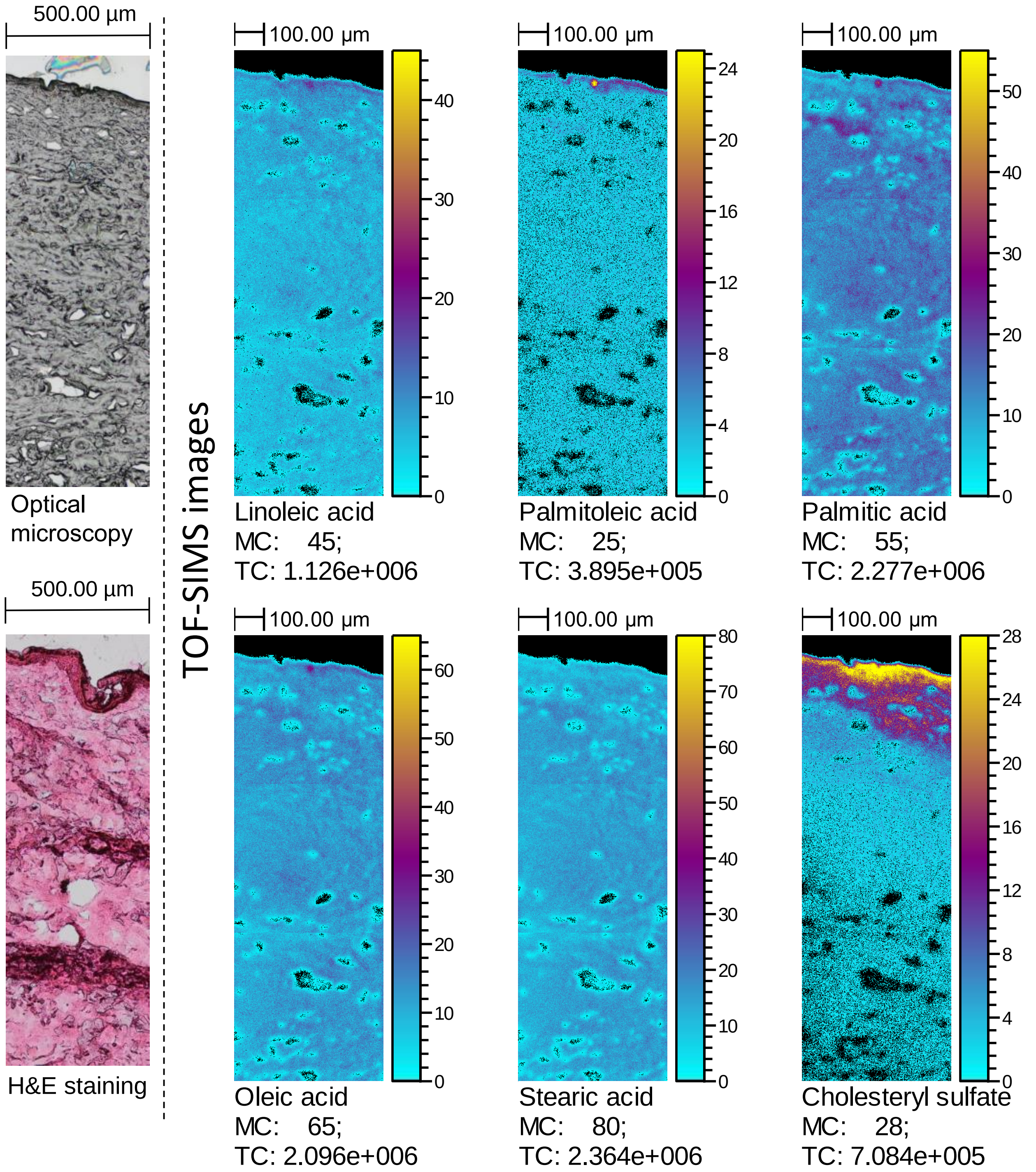

Human skin *ex vivo* sample treated with sea-buckthorn oil.

TOF-SIMS analysis in negative ionisation mode. Each ion image represents spatial localisation of FA analysed in the sample. MC – maximum ion counts detected per image; TC – total ion counts per image.

Optical image represents the sample before TOF-SIMS analysis. H&E staining represents a subsequent section stained with hematoxylin and eosin.

TOF-SIMS analysis of skin samples treated with raspberry seed oil

Skin sample treated with raspberry seed oil (1)

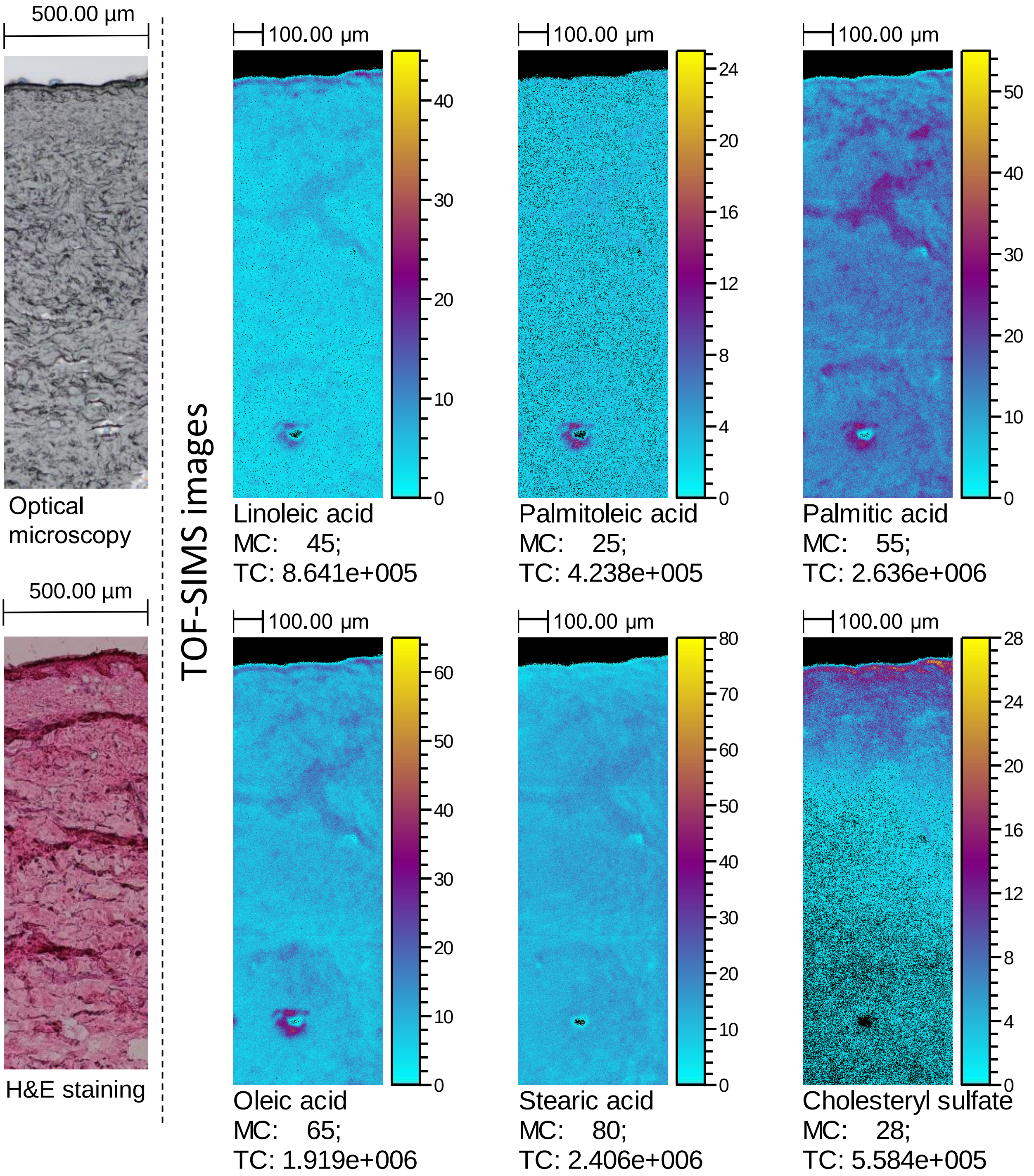

Human skin *ex vivo* sample treated with raspberry seed oil.
TOF-SIMS analysis in negative ionisation mode. Each ion image represents spatial localisation of FA analysed in the sample. MC – maximum ion counts detected per image; TC – total ion counts per image.
Optical image represents the sample before TOF-SIMS analysis. H&E staining represents a subsequent section stained with hematoxylin and eosin.

Skin sample treated with raspberry seed oil (2)

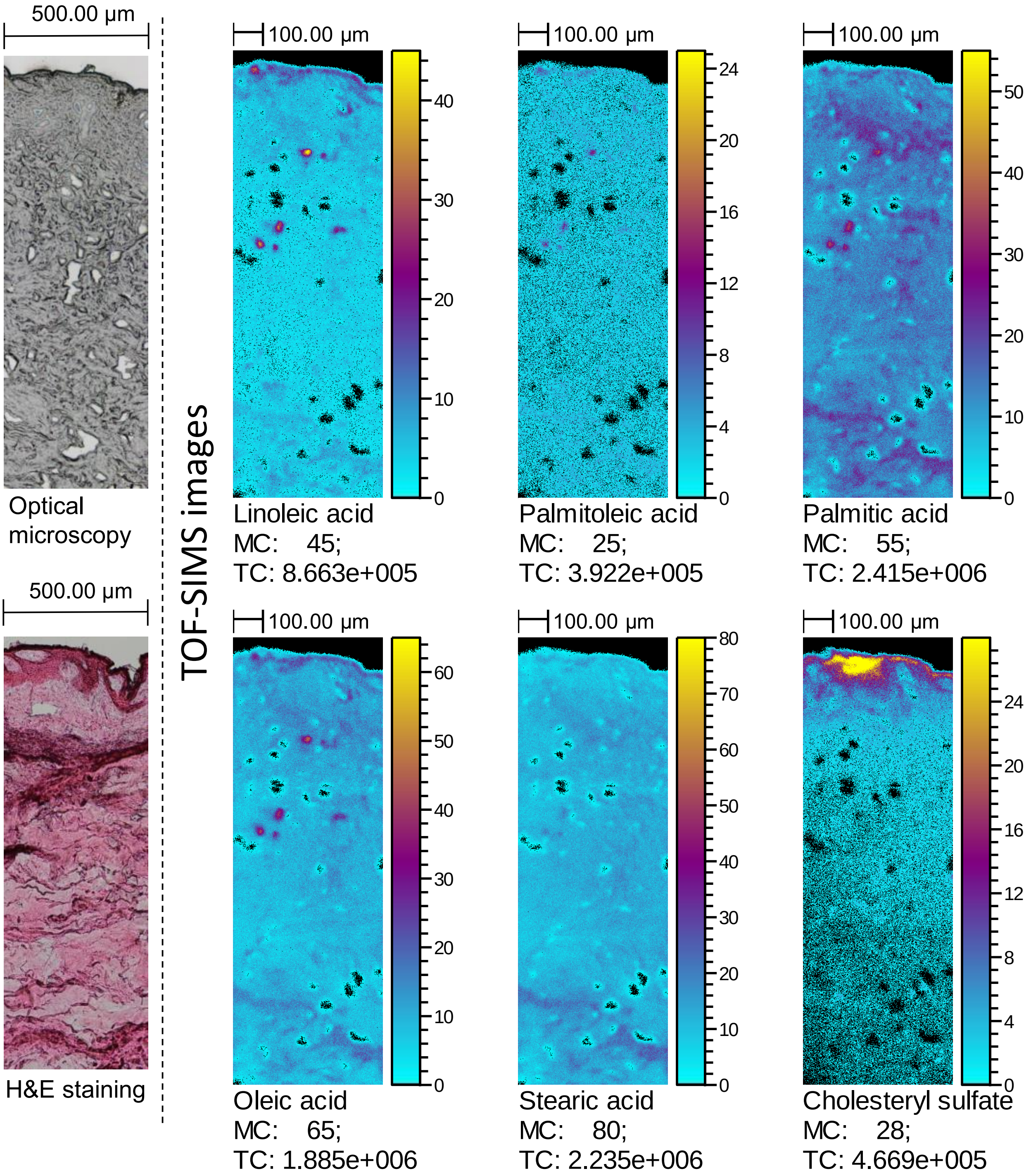

Human skin *ex vivo* sample treated with raspberry seed oil.
TOF-SIMS analysis in negative ionisation mode. Each ion image represents spatial localisation of FA analysed in the sample. MC – maximum ion counts detected per image; TC – total ion counts per image.
Optical image represents the sample before TOF-SIMS analysis. H&E staining represents a subsequent section stained with hematoxylin and eosin.

Skin sample treated with raspberry seed oil (3)

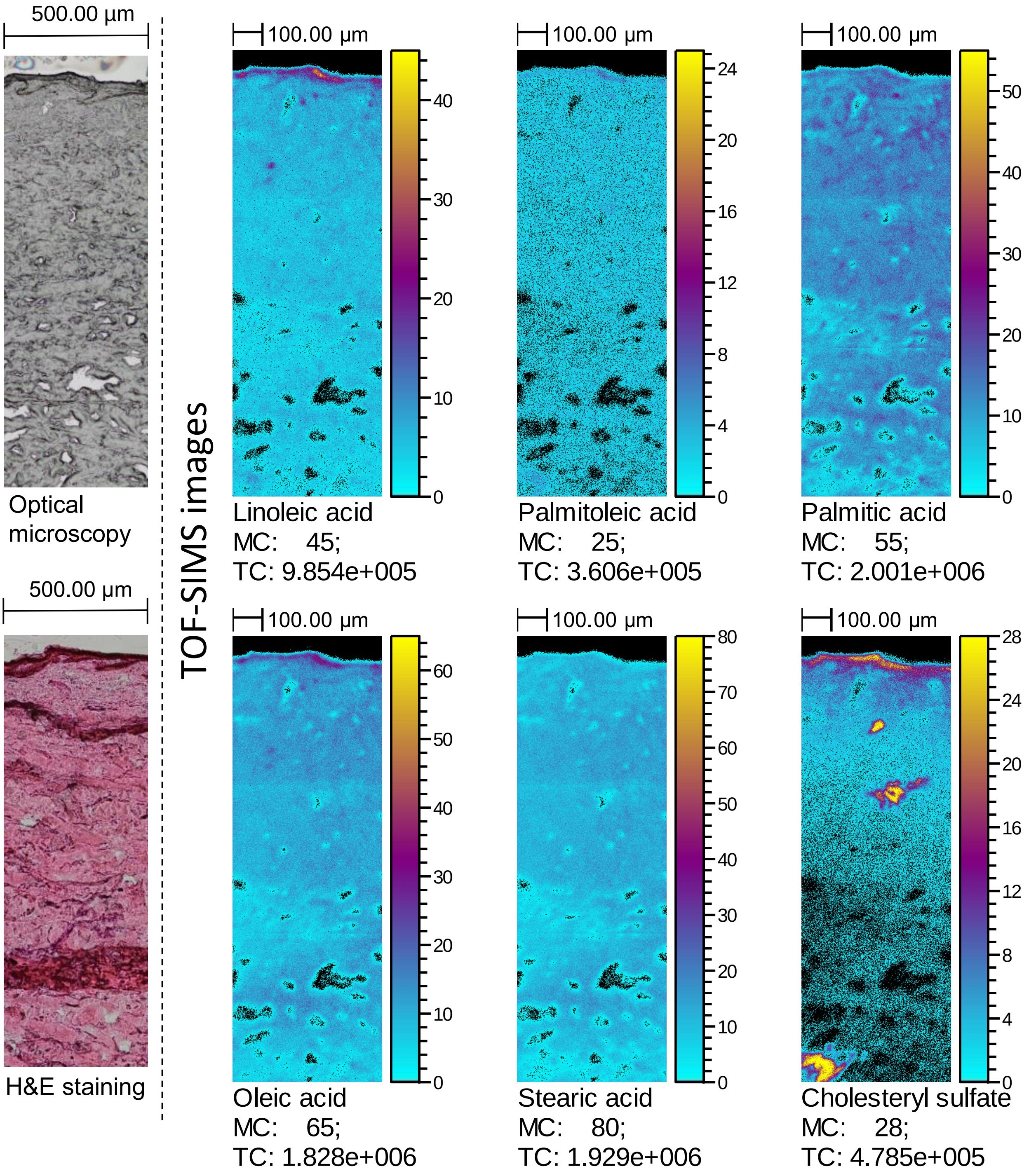

Human skin *ex vivo* sample treated with raspberry seed oil.
TOF-SIMS analysis in negative ionisation mode. Each ion image represents spatial localisation of FA analysed in the sample. MC – maximum ion counts detected per image; TC – total ion counts per image.
Optical image represents the sample before TOF-SIMS analysis. H&E staining represents a subsequent section stained with hematoxylin and eosin.

TOF-SIMS analysis of skin samples treated with coconut oil

Skin sample treated with coconut oil (1)

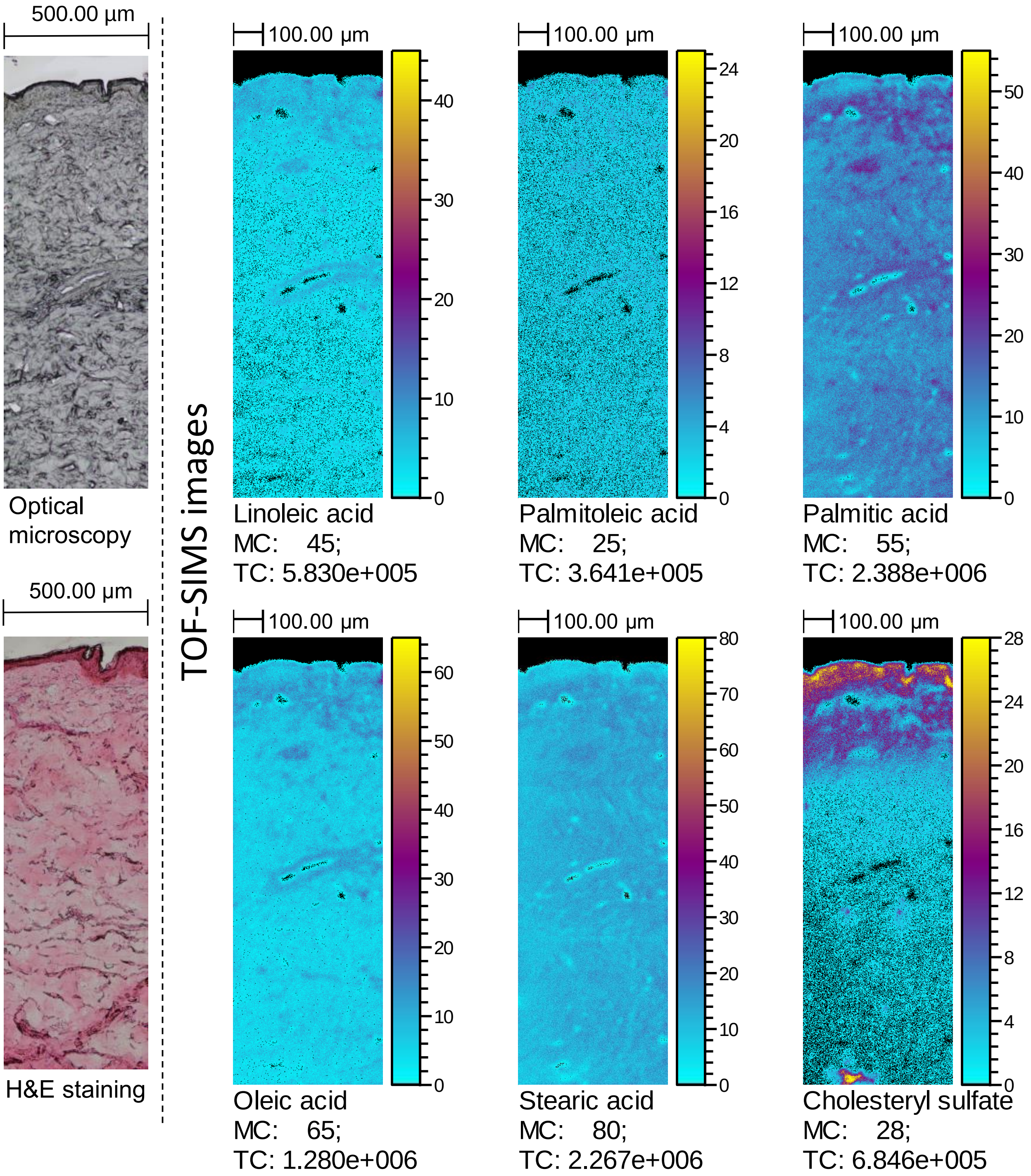

Human skin *ex vivo* sample treated with coconut oil.

TOF-SIMS analysis in negative ionisation mode. Each ion image represents spatial localisation of FA analysed in the sample. MC – maximum ion counts detected per image; TC – total ion counts per image.

Optical image represents the sample before TOF-SIMS analysis. H&E staining represents a subsequent section stained with hematoxylin and eosin.

Skin sample treated with coconut oil (2)

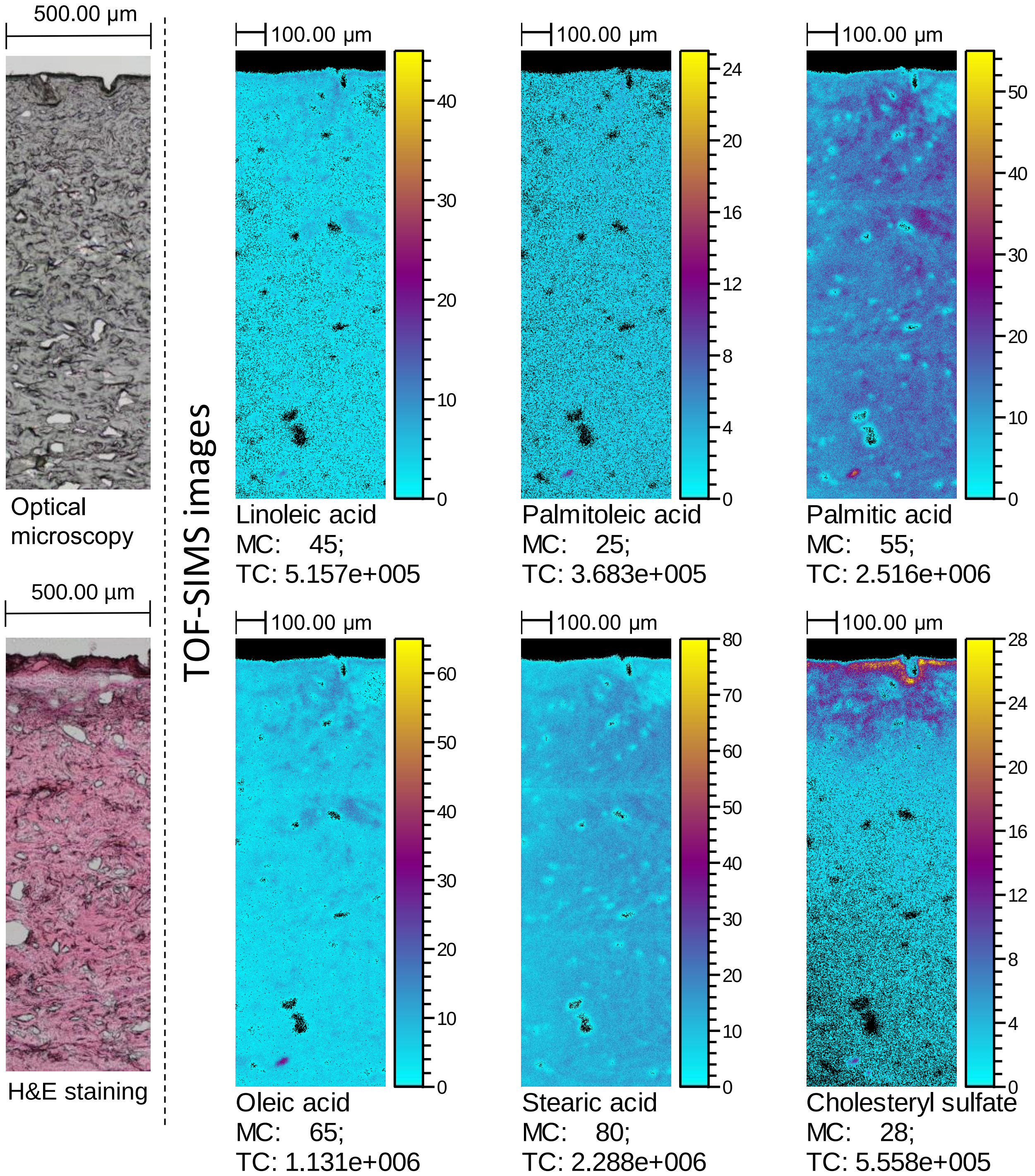

Human skin *ex vivo* sample treated with coconut oil.
TOF-SIMS analysis in negative ionisation mode. Each ion image represents spatial localisation of FA analysed in the sample. MC – maximum ion counts detected per image; TC – total ion counts per image.
Optical image represents the sample before TOF-SIMS analysis. H&E staining represents a subsequent section stained with hematoxylin and eosin.

Skin sample treated with coconut oil (3)

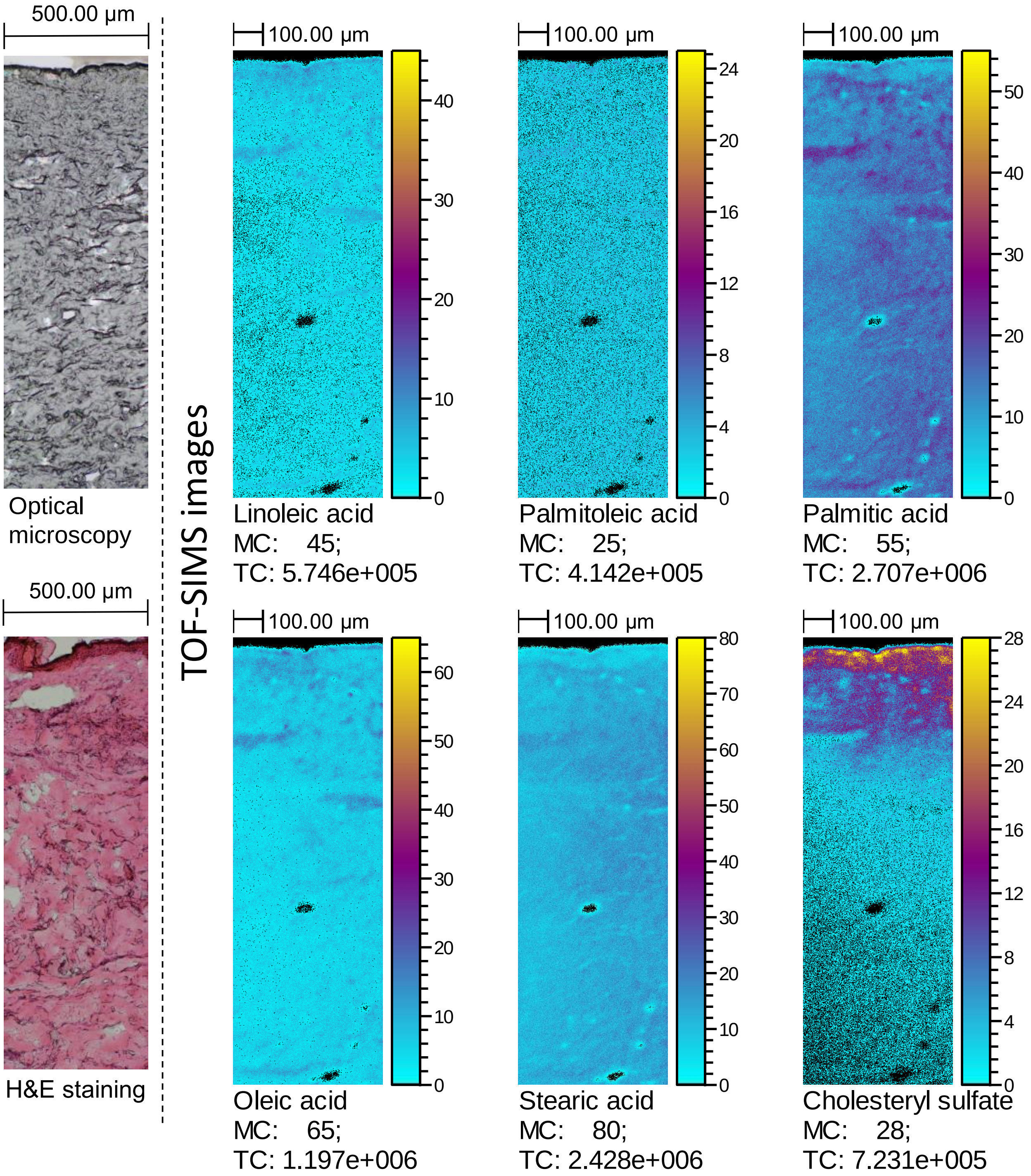

Human skin *ex vivo* sample treated with coconut oil.

TOF-SIMS analysis in negative ionisation mode. Each ion image represents spatial localisation of FA analysed in the sample. MC – maximum ion counts detected per image; TC – total ion counts per image.

Optical image represents the sample before TOF-SIMS analysis. H&E staining represents a subsequent section stained with hematoxylin and eosin.

TOF-SIMS analysis of skin samples treated with olive oil

Skin sample treated with olive oil (1)

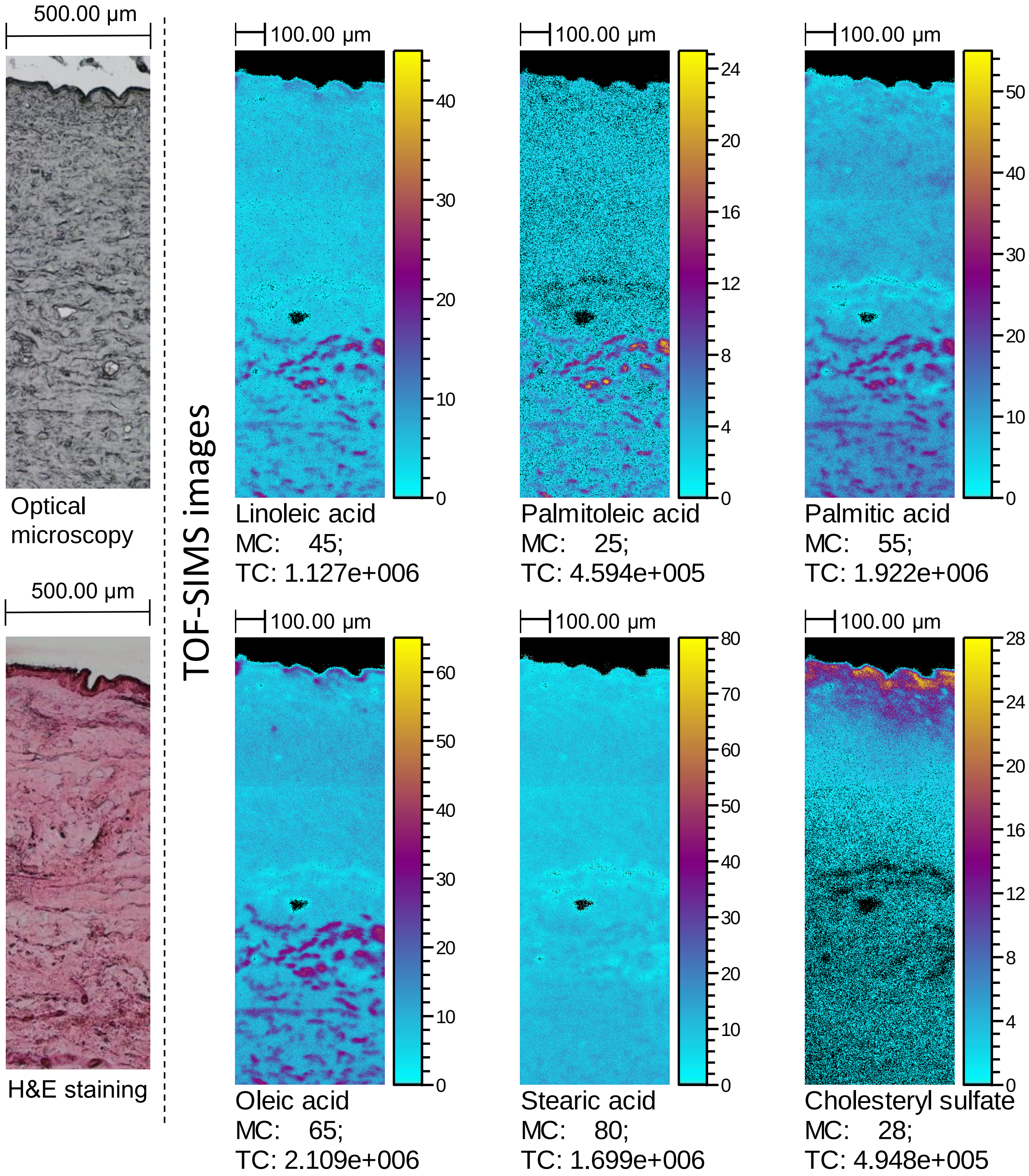

Human skin *ex vivo* sample treated with olive oil.
TOF-SIMS analysis in negative ionisation mode. Each ion image represents spatial localisation of FA analysed in the sample. MC – maximum ion counts detected per image; TC – total ion counts per image.
Optical image represents the sample before TOF-SIMS analysis. H&E staining represents a subsequent section stained with hematoxylin and eosin.

Skin sample treated with olive oil (2)

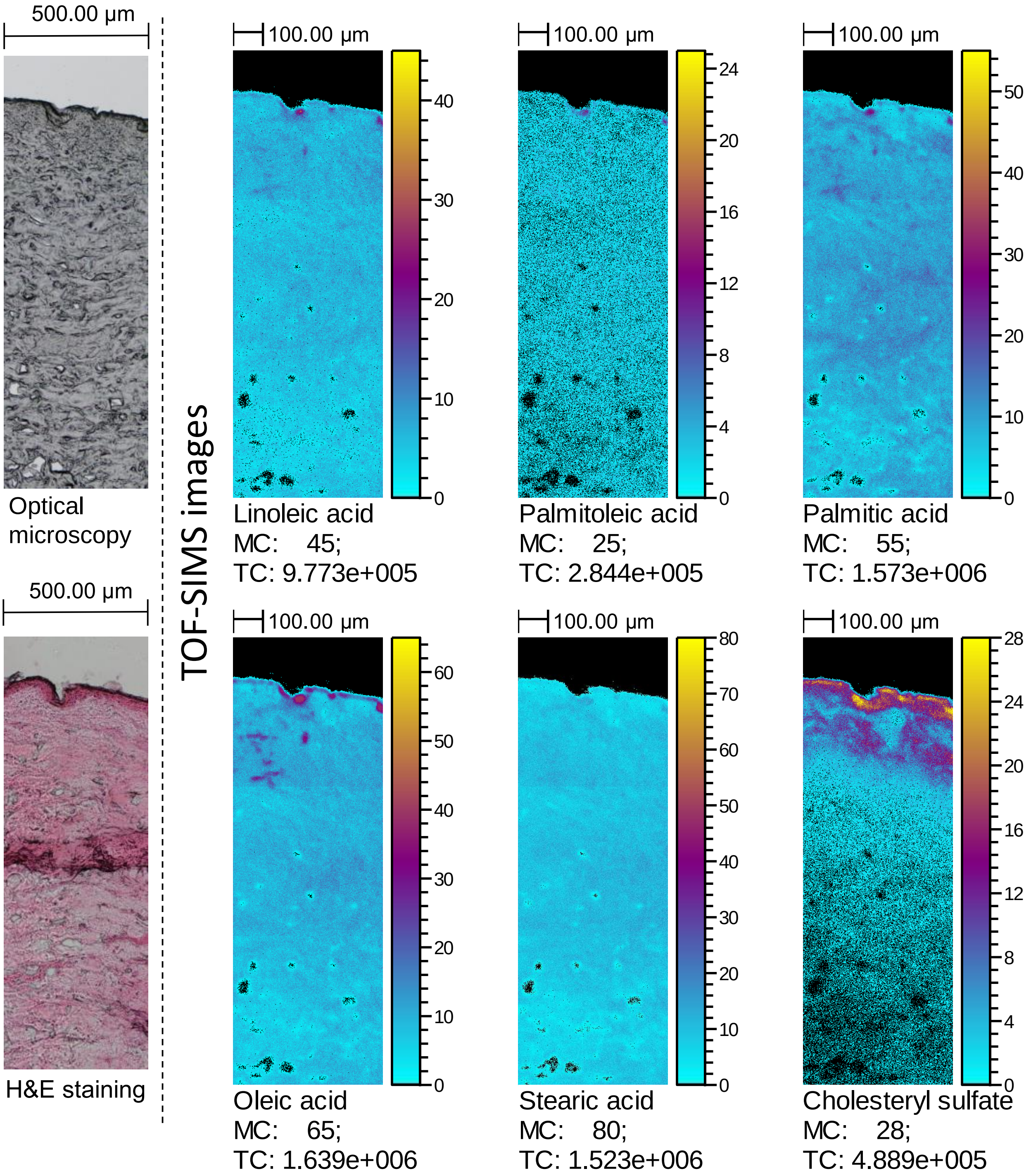

Human skin *ex vivo* sample treated with olive oil.
TOF-SIMS analysis in negative ionisation mode. Each ion image represents spatial localisation of FA analysed in the sample. MC – maximum ion counts detected per image; TC – total ion counts per image.
Optical image represents the sample before TOF-SIMS analysis. H&E staining represents a subsequent section stained with hematoxylin and eosin.

Skin sample treated with olive oil (3)

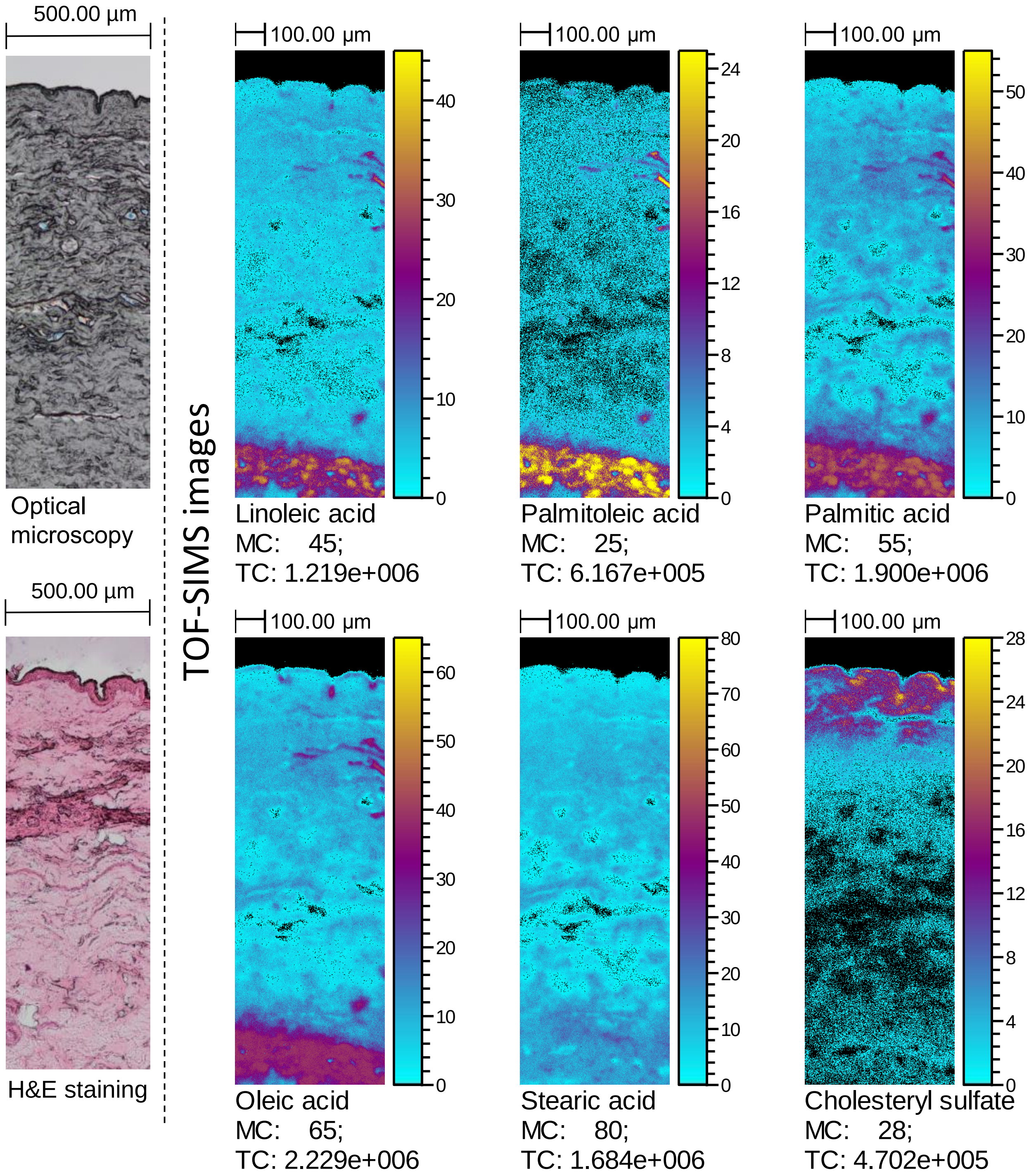

Human skin *ex vivo* sample treated with olive oil.
TOF-SIMS analysis in negative ionisation mode. Each ion image represents spatial localisation of FA analysed in the sample. MC – maximum ion counts detected per image; TC – total ion counts per image.
Optical image represents the sample before TOF-SIMS analysis. H&E staining represents a subsequent section stained with hematoxylin and eosin.

TOF-SIMS analysis of skin samples treated with soybean oil

Skin sample treated with soybean oil (1)

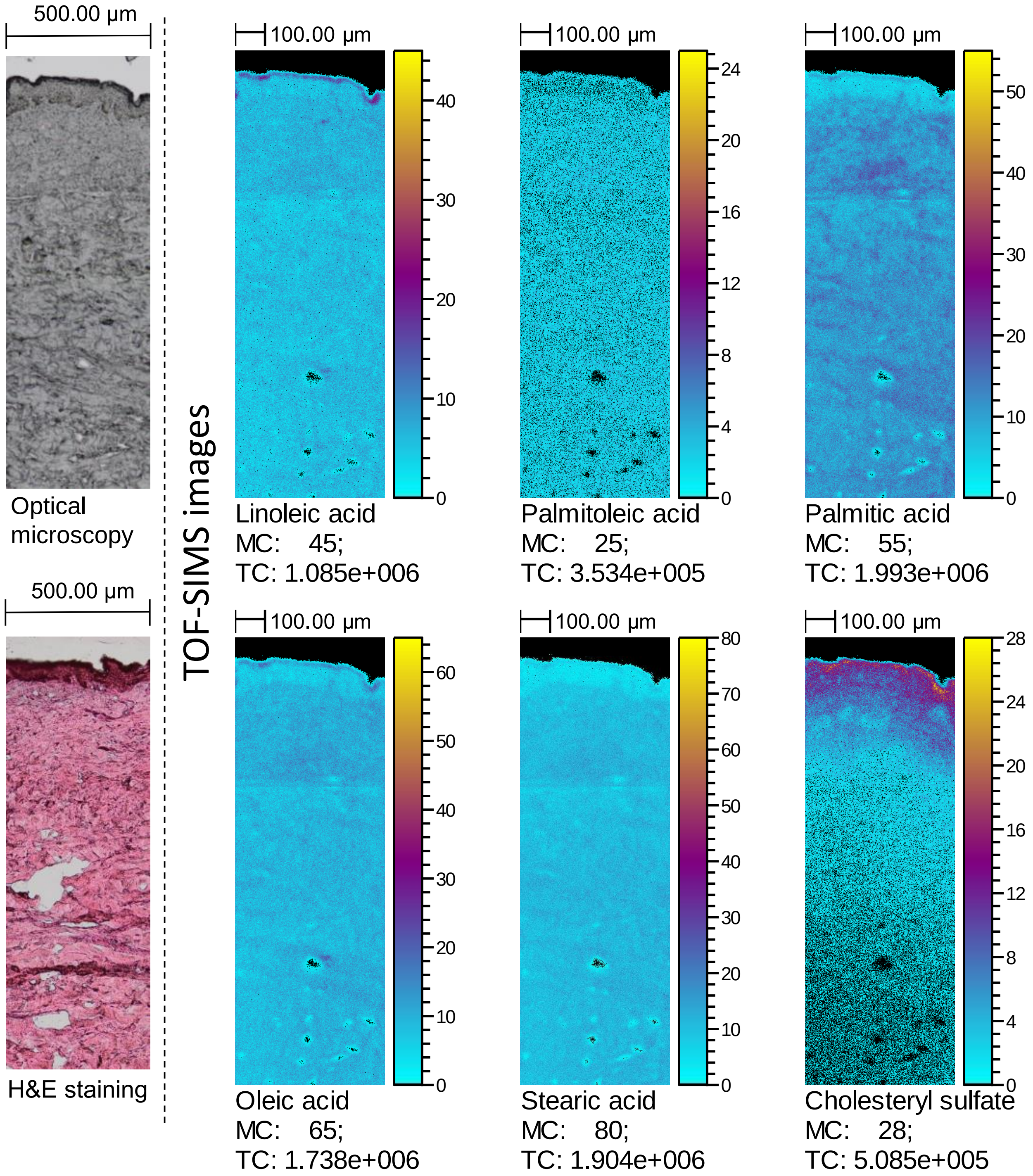

Human skin *ex vivo* sample treated with soybean oil.
TOF-SIMS analysis in negative ionisation mode. Each ion image represents spatial localisation of FA analysed in the sample. MC – maximum ion counts detected per image; TC – total ion counts per image.
Optical image represents the sample before TOF-SIMS analysis. H&E staining represents a subsequent section stained with hematoxylin and eosin.

Skin sample treated with soybean oil (2)

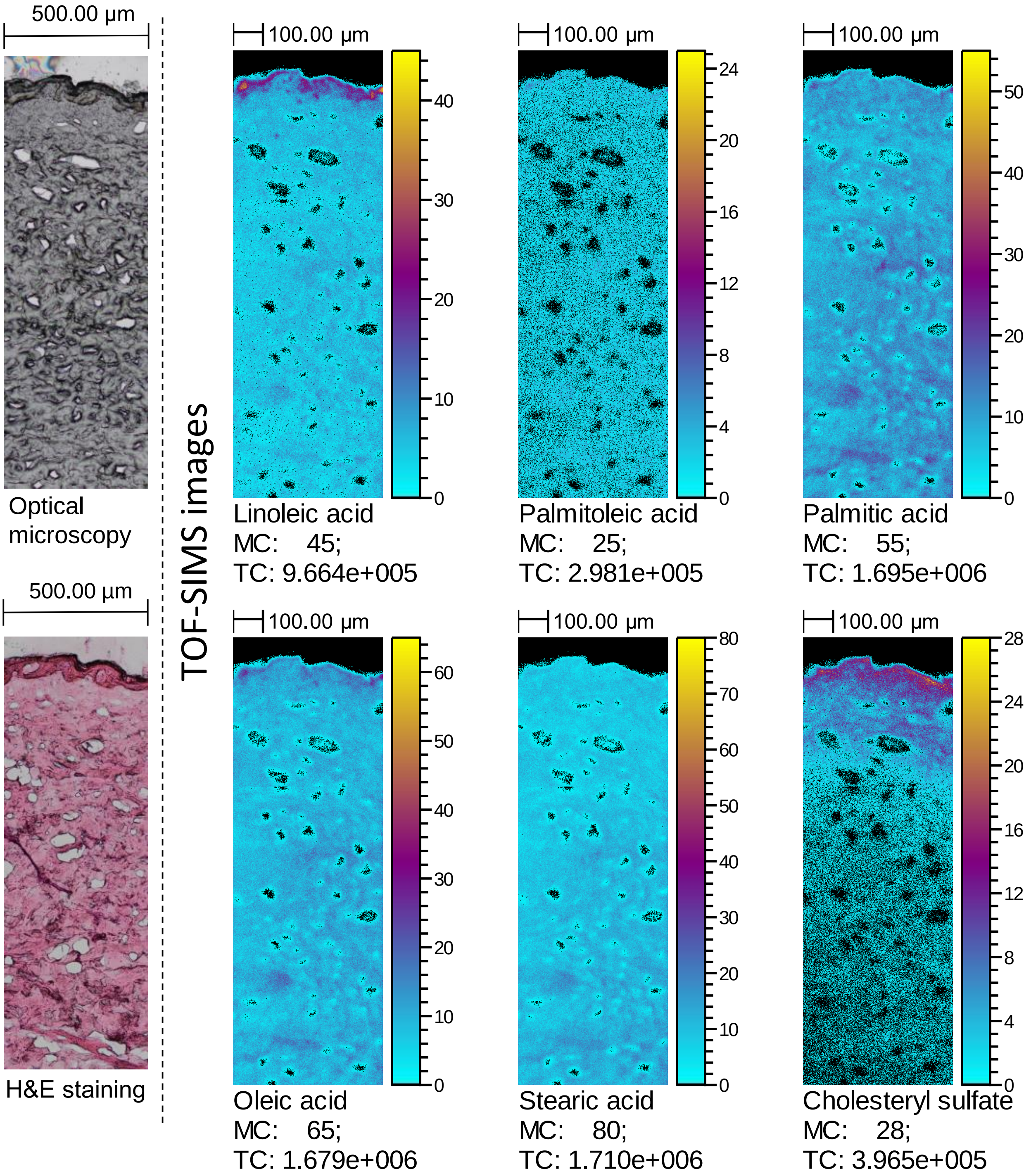

Human skin *ex vivo* sample treated with soybean oil.
TOF-SIMS analysis in negative ionisation mode. Each ion image represents spatial localisation of FA analysed in the sample. MC – maximum ion counts detected per image; TC – total ion counts per image.
Optical image represents the sample before TOF-SIMS analysis. H&E staining represents a subsequent section stained with hematoxylin and eosin.

Skin sample treated with soybean oil (3)

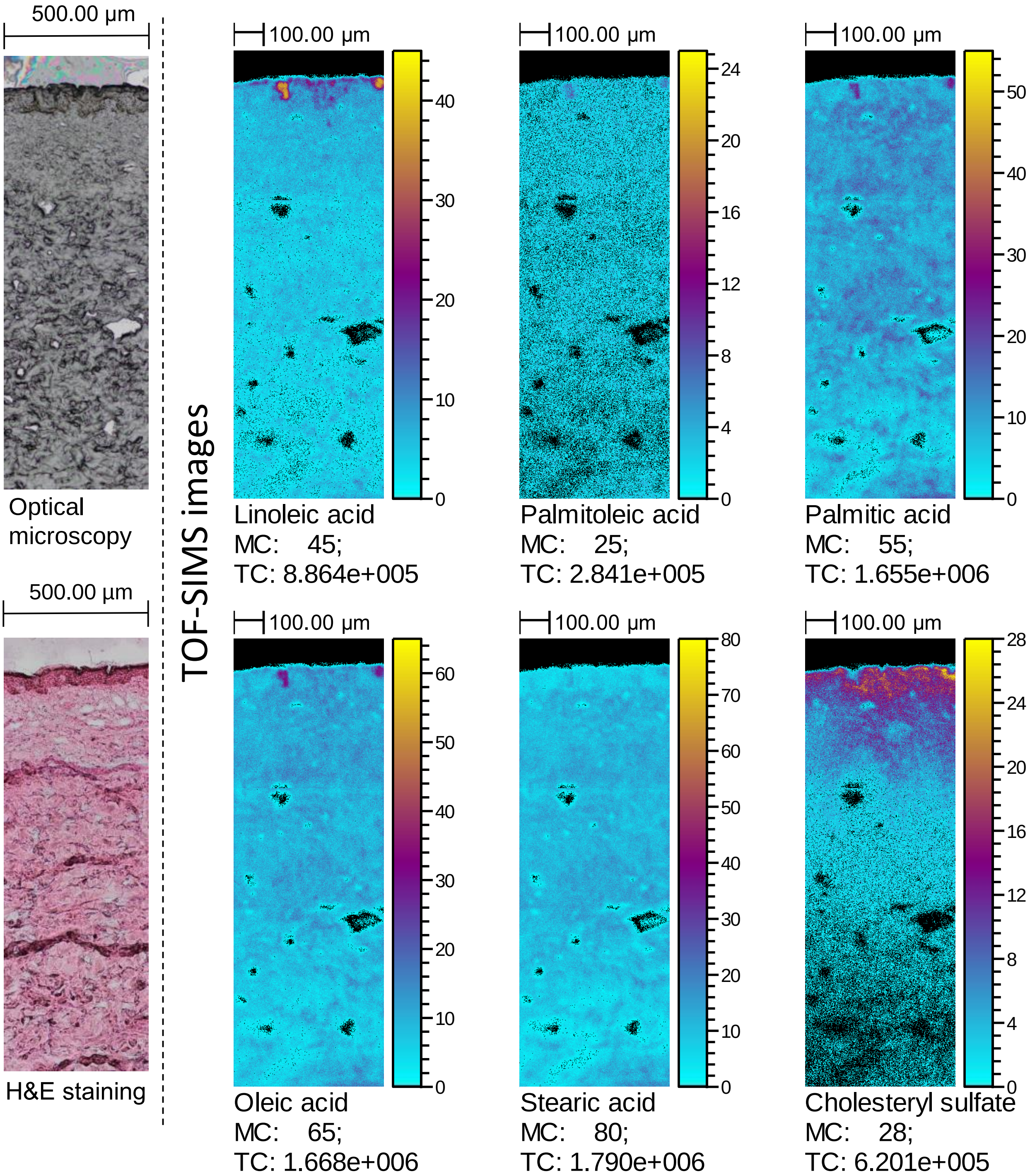

Human skin *ex vivo* sample treated with soybean oil.

TOF-SIMS analysis in negative ionisation mode. Each ion image represents spatial localisation of FA analysed in the sample. MC – maximum ion counts detected per image; TC – total ion counts per image.

Optical image represents the sample before TOF-SIMS analysis. H&E staining represents a subsequent section stained with hematoxylin and eosin.

TOF-SIMS analysis of skin samples treated with avocado oil

Skin sample treated with avocado oil (1)

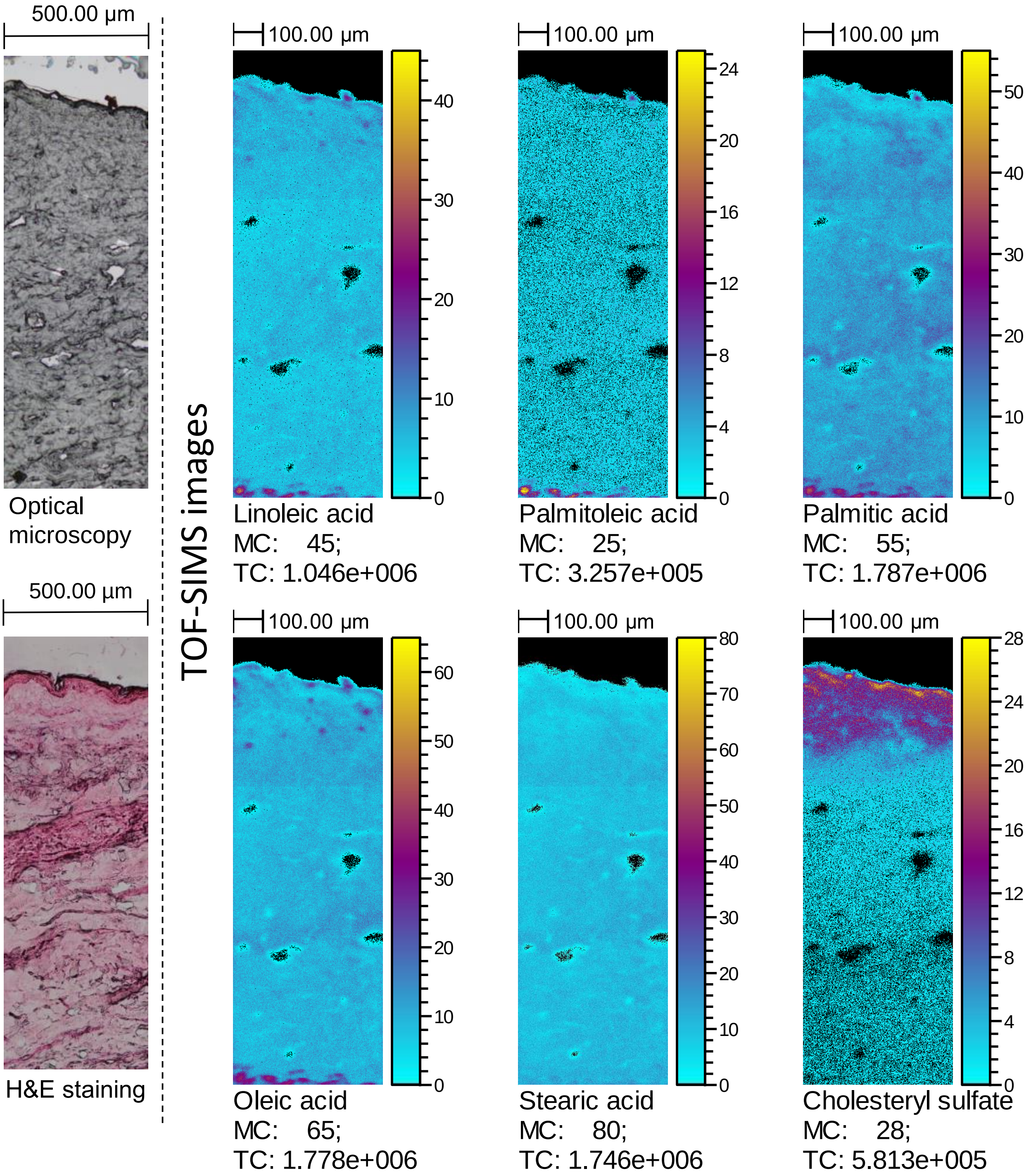

Human skin *ex vivo* sample treated with avocado oil.

TOF-SIMS analysis in negative ionisation mode. Each ion image represents spatial localisation of FA analysed in the sample. MC – maximum ion counts detected per image; TC – total ion counts per image.

Optical image represents the sample before TOF-SIMS analysis. H&E staining represents a subsequent section stained with hematoxylin and eosin.

Skin sample treated with avocado oil (2)

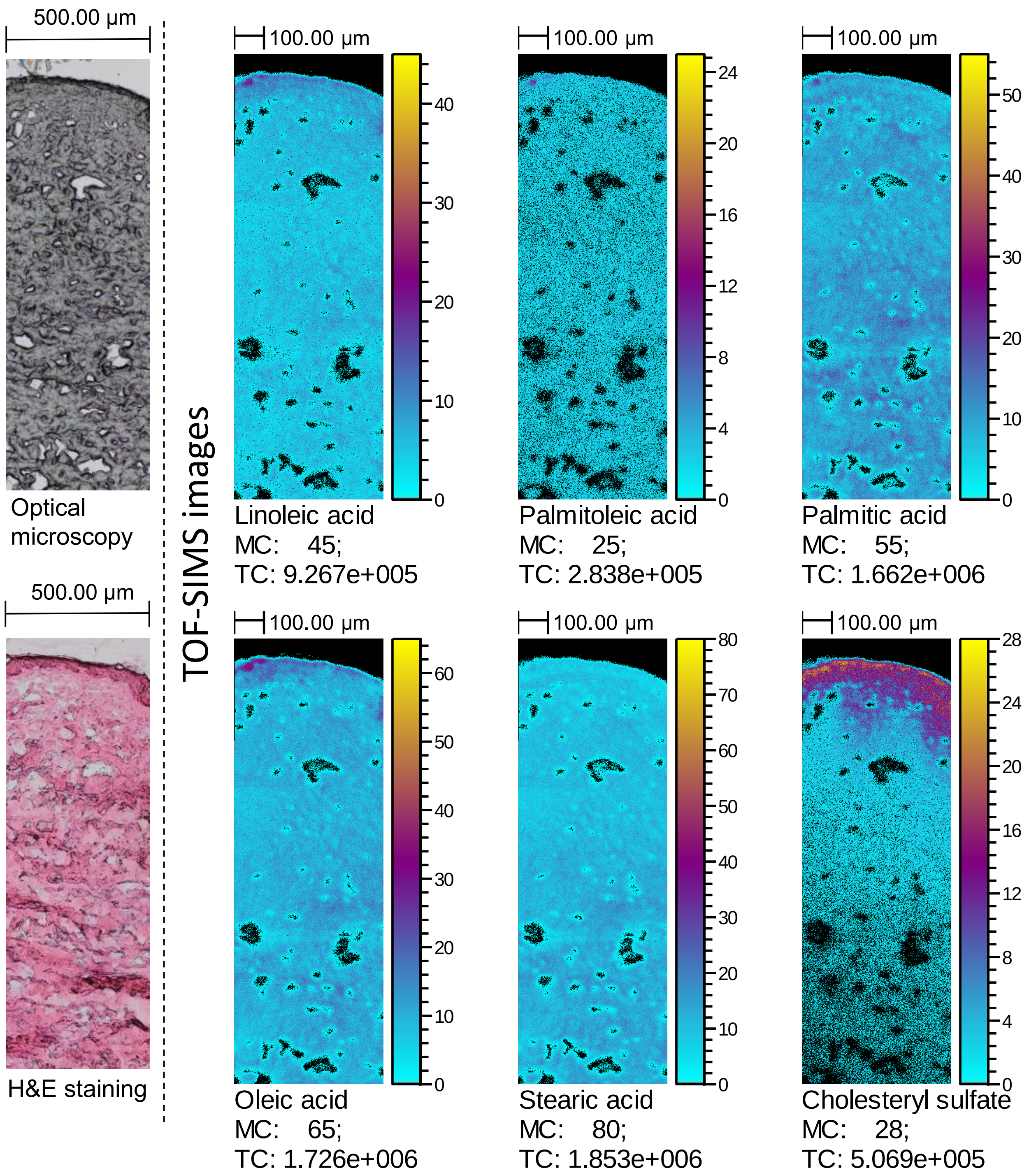

Human skin *ex vivo* sample treated with avocado oil.
TOF-SIMS analysis in negative ionisation mode. Each ion image represents spatial localisation of FA analysed in the sample. MC – maximum ion counts detected per image; TC – total ion counts per image.
Optical image represents the sample before TOF-SIMS analysis. H&E staining represents a subsequent section stained with hematoxylin and eosin.

Skin sample treated with avocado oil (3)

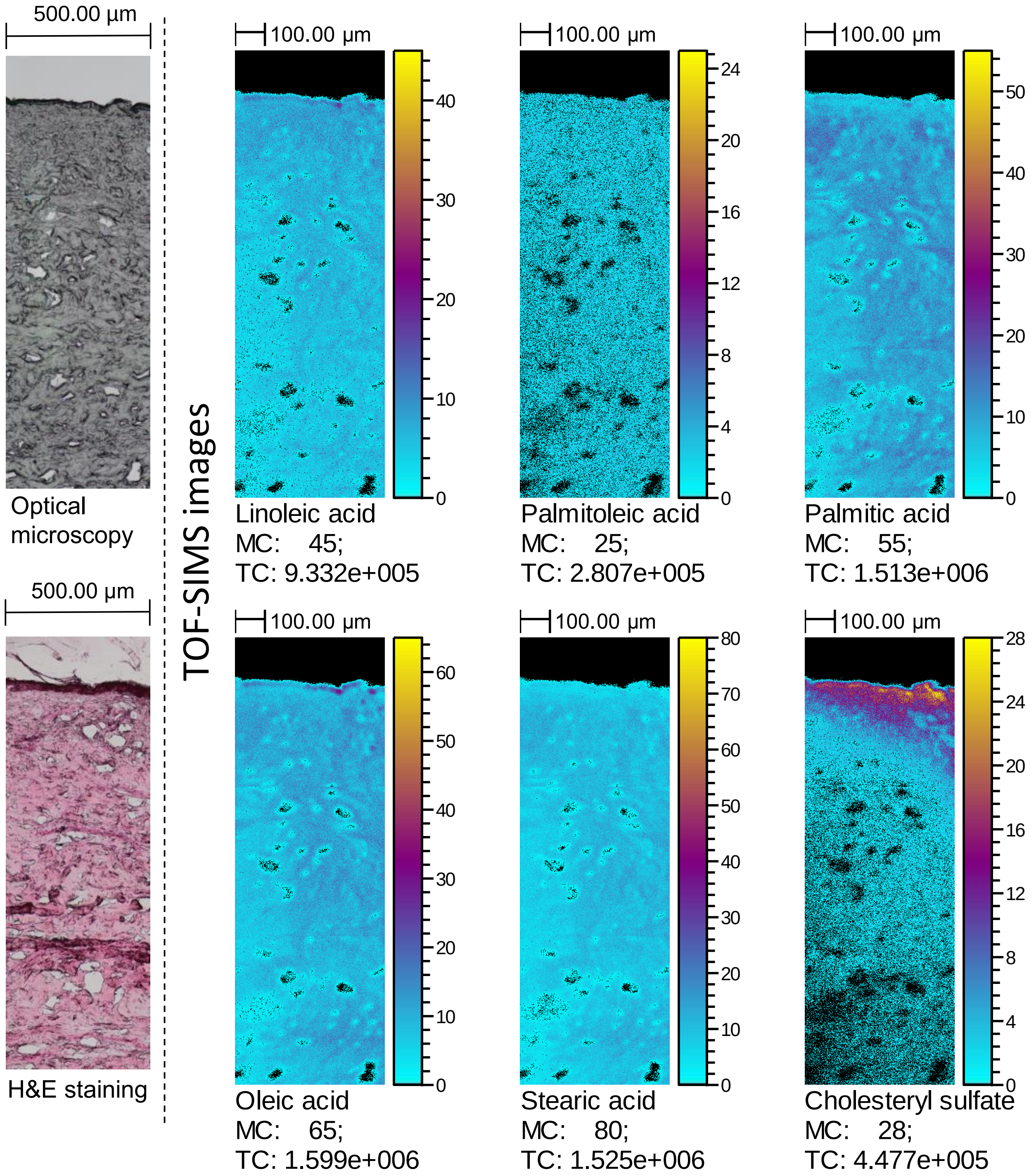

Human skin *ex vivo* sample treated with avocado oil.

TOF-SIMS analysis in negative ionisation mode. Each ion image represents spatial localisation of FA analysed in the sample. MC – maximum ion counts detected per image; TC – total ion counts per image.

Optical image represents the sample before TOF-SIMS analysis. H&E staining represents a subsequent section stained with hematoxylin and eosin.

Ion intensity profiles of fatty acids as a function of depth

Ion intensity profiles of fatty acids in the skin samples treated with avocado oil

Linoleic acid intensity profiles

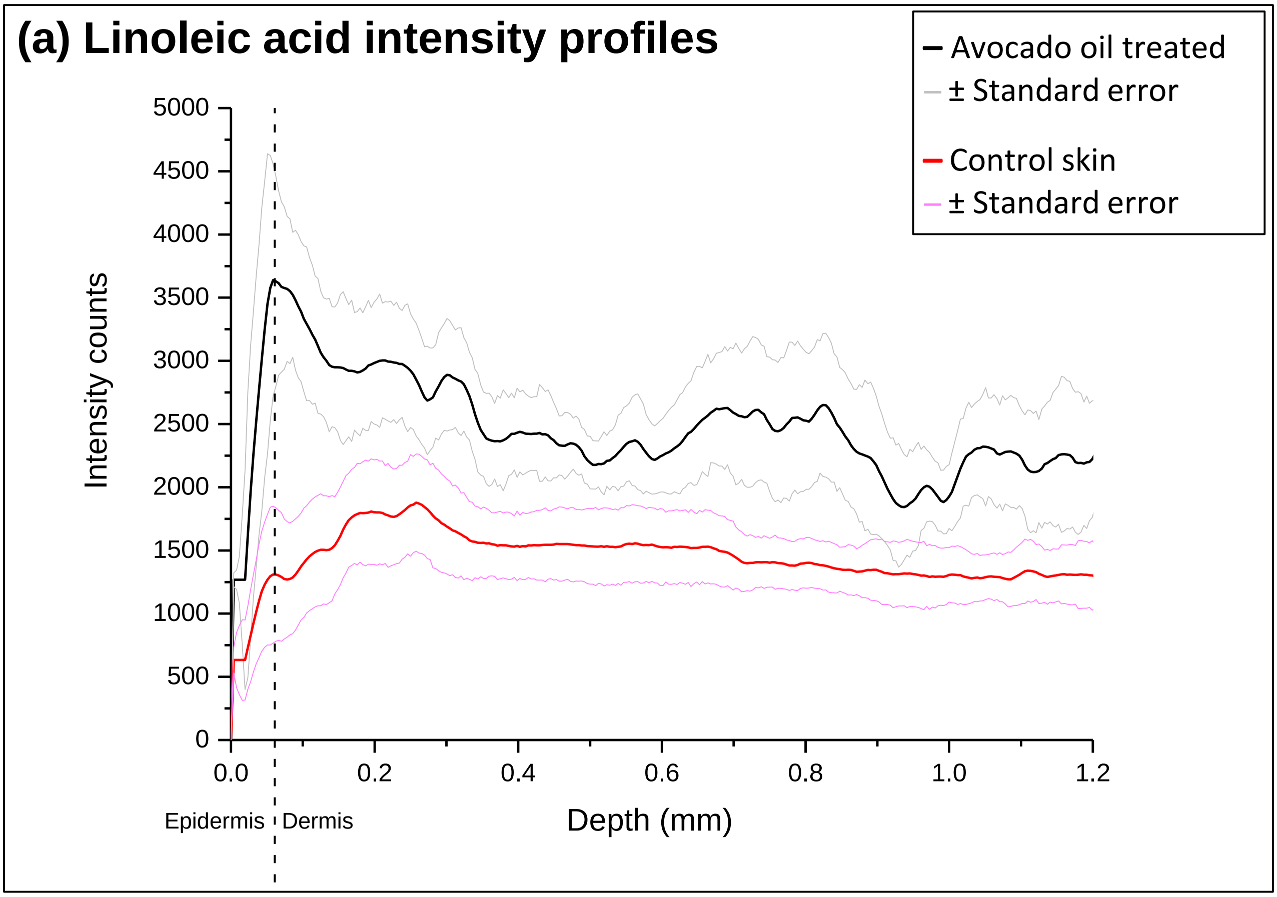

Linoleic acid ion intensity profile as the function of depth in avocado oil treated skin samples and compared to the profile obtained from the control skin samples.
Black line corresponds to the average ion intensity values obtained from integrated ion images of skin samples treated with avocado oil; red line corresponds to the profile obtained in control skin samples.

Palmitoleic acid intensity profiles

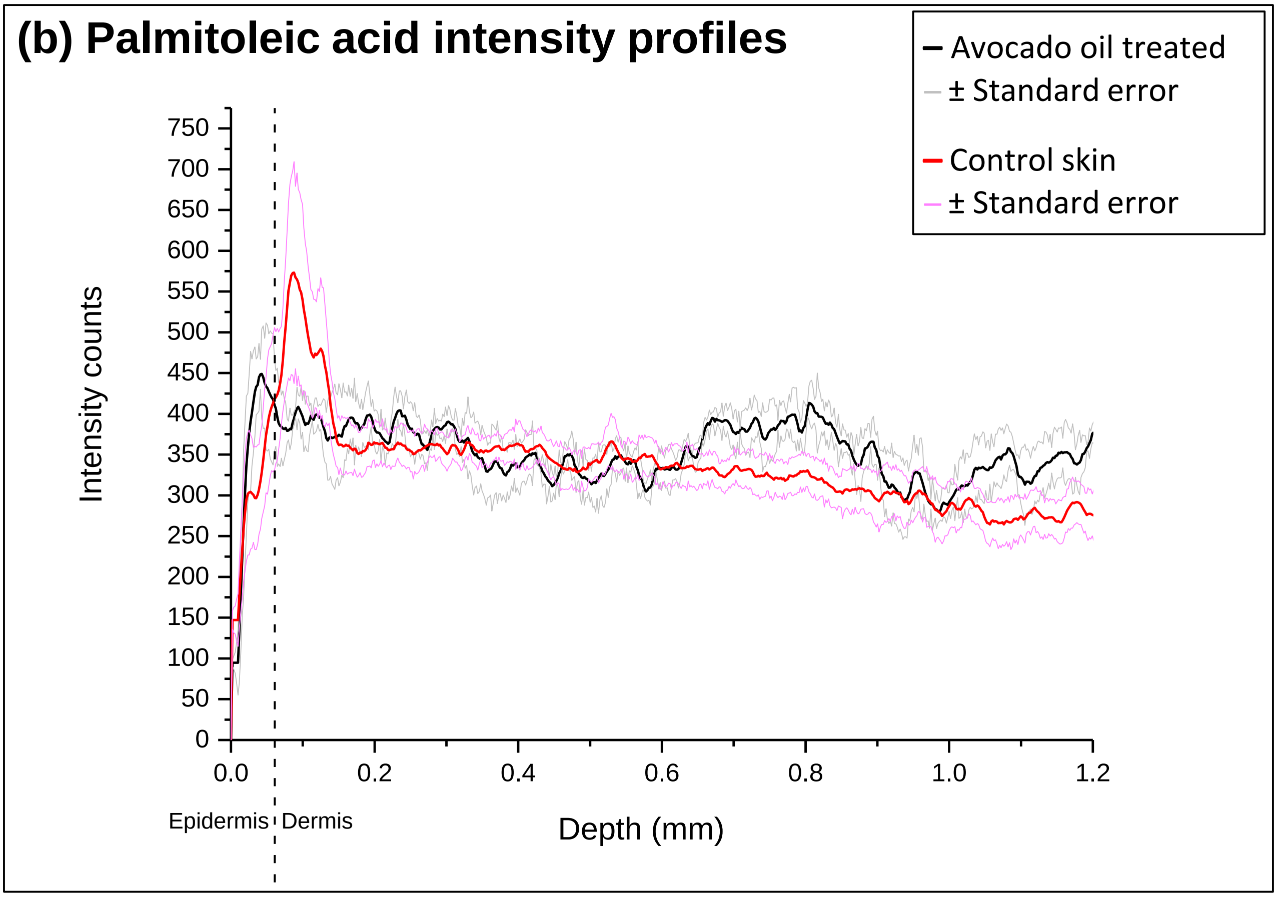

Palmitoleic acid ion intensity profile as the function of depth in avocado oil treated skin samples and compared to the profile obtained from the control skin samples.

Black line corresponds to the average ion intensity values obtained from integrated ion images of skin samples treated with avocado oil; red line corresponds to the profile obtained in control skin samples.

Palmitic acid intensity profiles

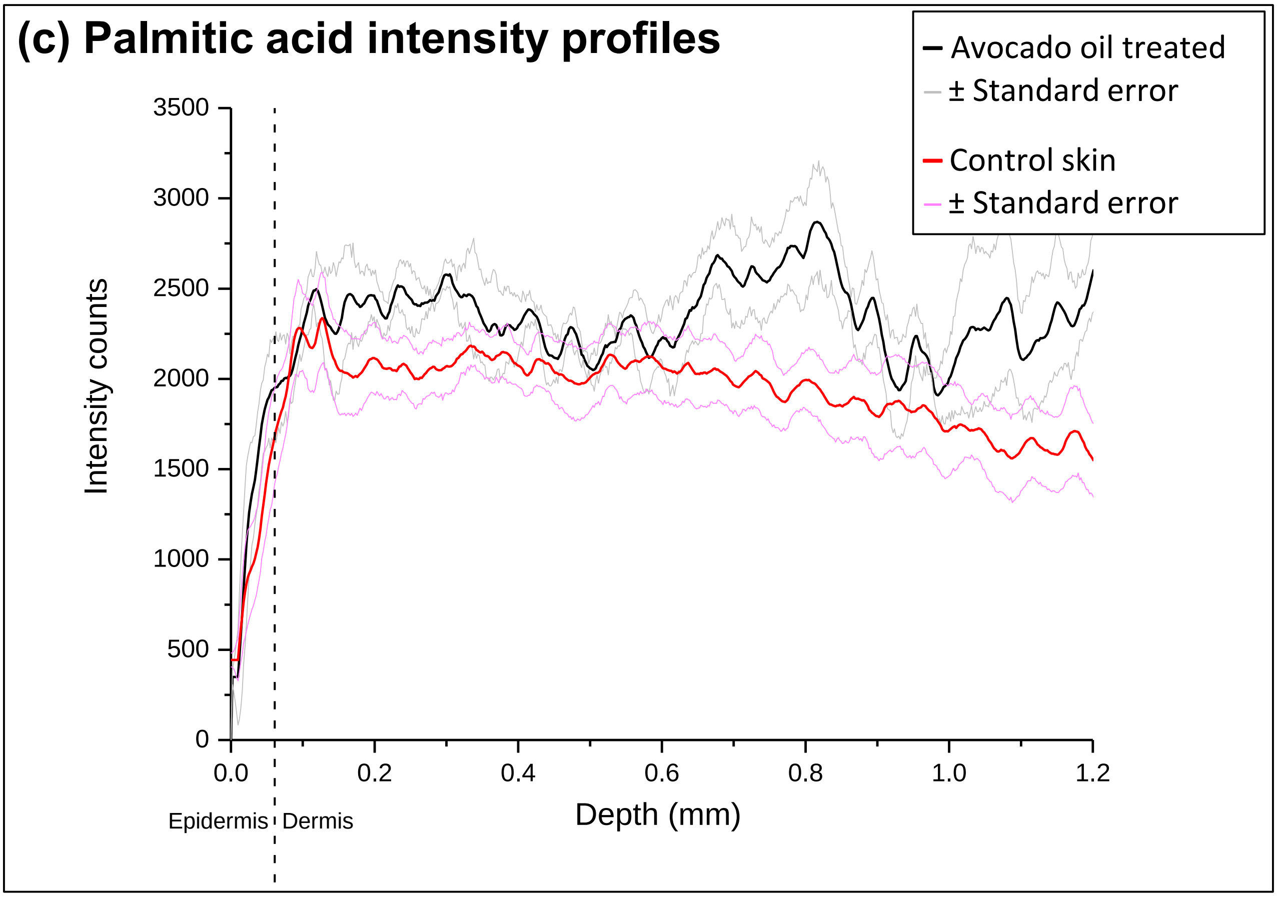

Palmitic acid ion intensity profile as the function of depth in avocado oil treated skin samples and compared to the profile obtained from the control skin samples.

Black line corresponds to the average ion intensity values obtained from integrated ion images of skin samples treated with avocado oil; red line corresponds to the profile obtained in control skin samples.

Oleic acid intensity profiles

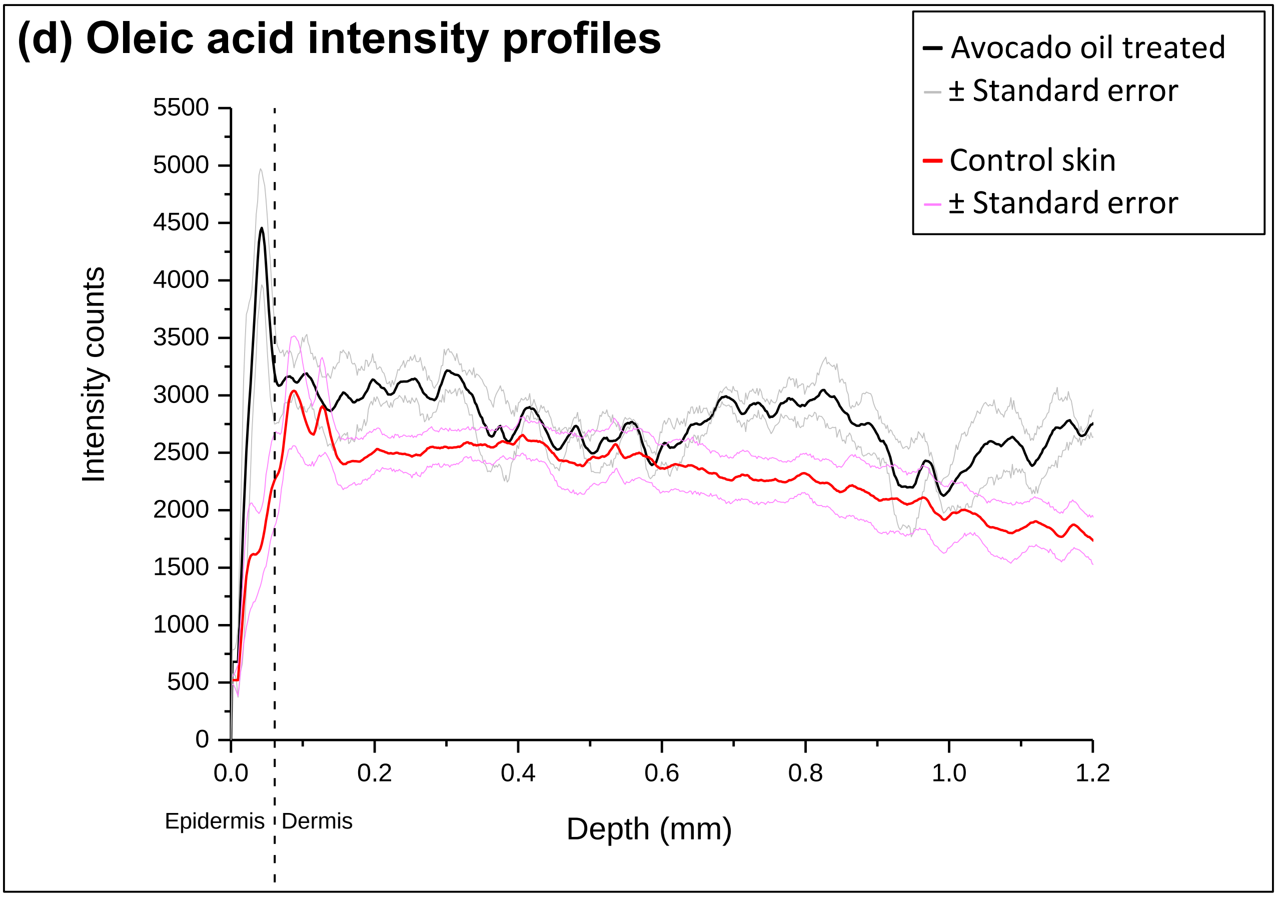

Oleic acid ion intensity profile as the function of depth in avocado oil treated skin samples and compared to the profile obtained from the control skin samples. Black line corresponds to the average ion intensity values obtained from integrated ion images of skin samples treated with avocado oil; red line corresponds to the profile obtained in control skin samples.

Stearic acid intensity profiles

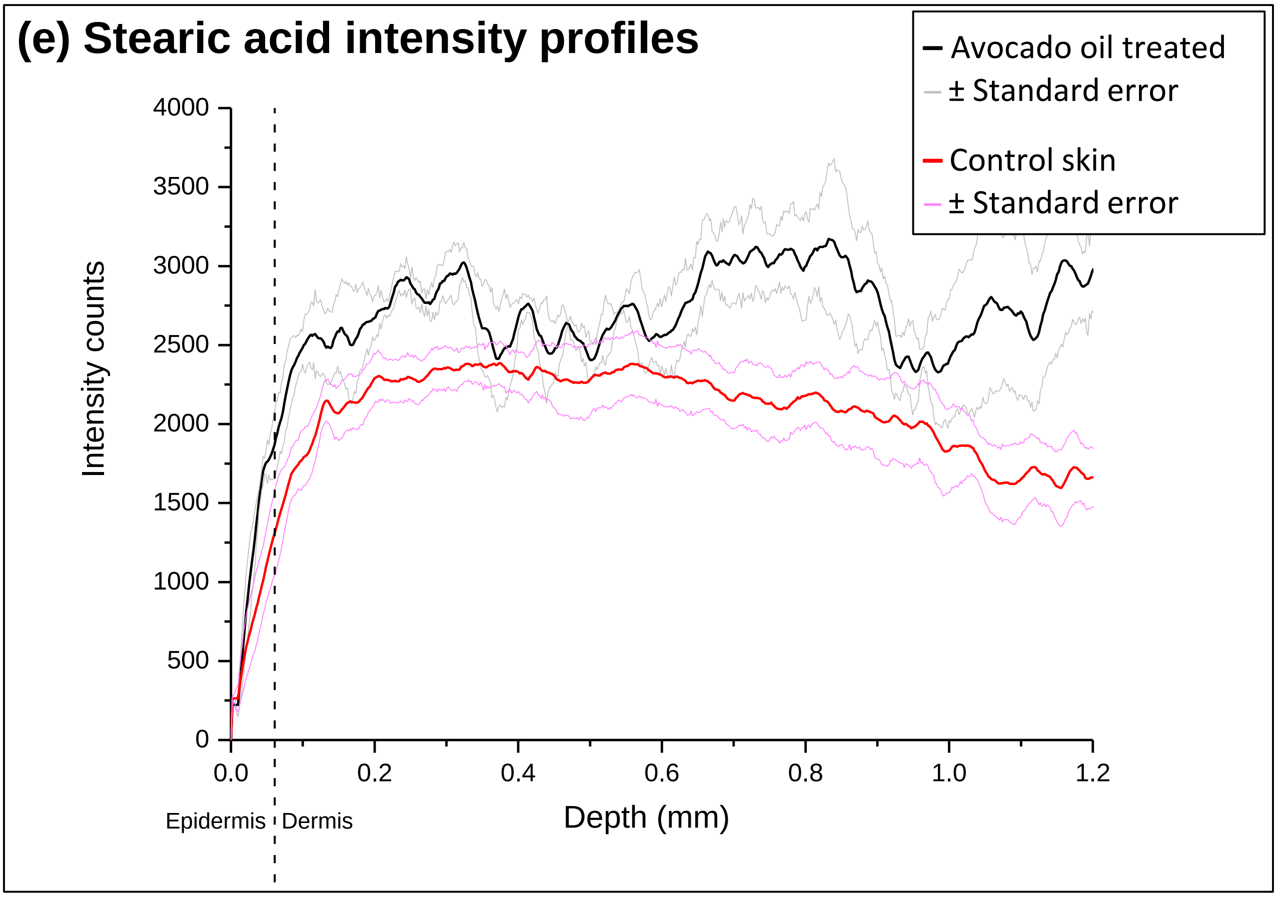

Stearic acid ion intensity profile as the function of depth in avocado oil treated skin samples and compared to the profile obtained from the control skin samples.

Black line corresponds to the average ion intensity values obtained from integrated ion images of skin samples treated with avocado oil; red line corresponds to the profile obtained in control skin samples.

Ion intensity profiles of fatty acids in the skin samples treated with coconut oil

Linoleic acid intensity profiles

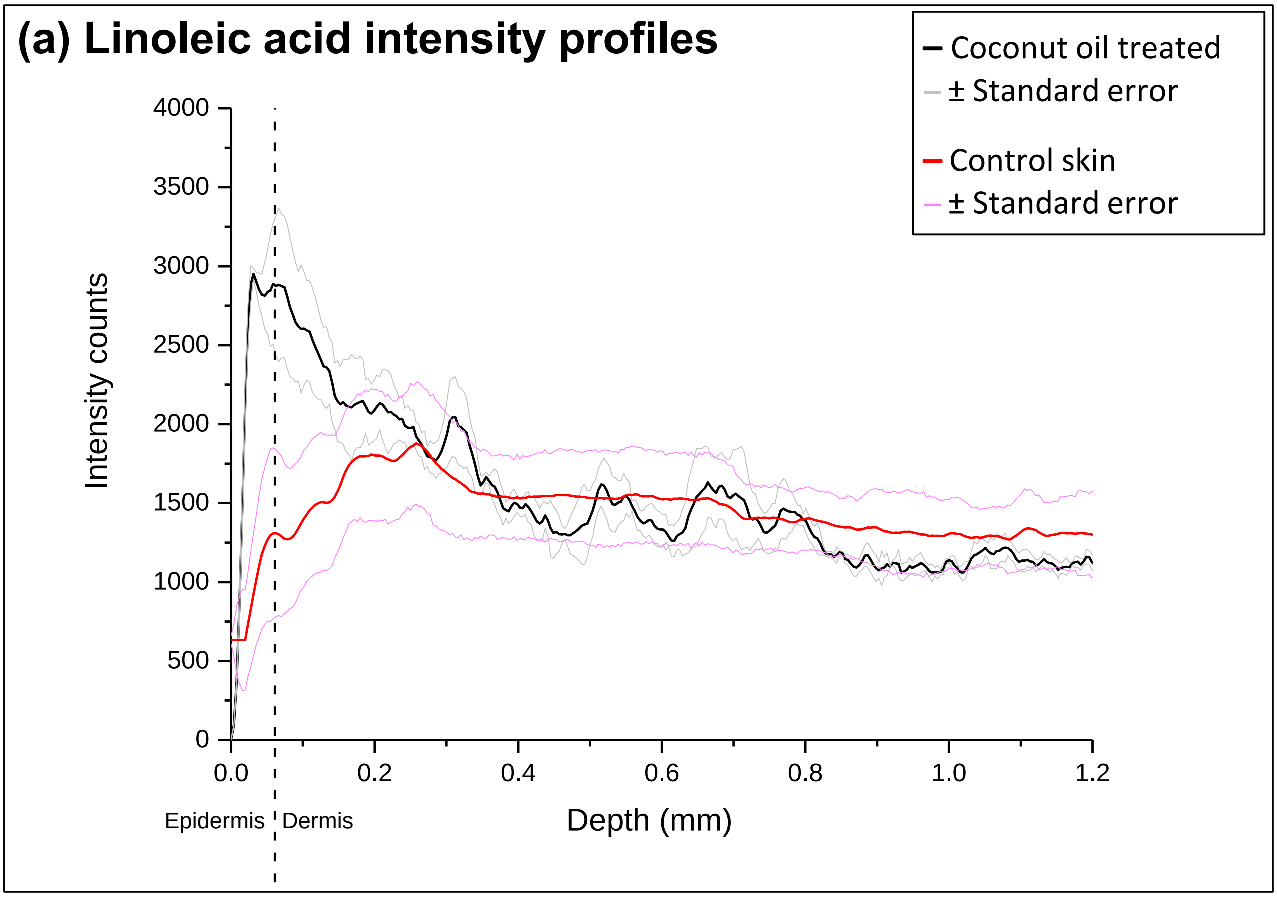

Linoleic acid ion intensity profile as the function of depth in coconut oil treated skin samples and compared to the profile obtained from the control skin samples.

Black line corresponds to the average ion intensity values obtained from integrated ion images of skin samples treated with coconut oil; red line corresponds to the profile obtained in control skin samples.

Palmitoleic acid intensity profiles

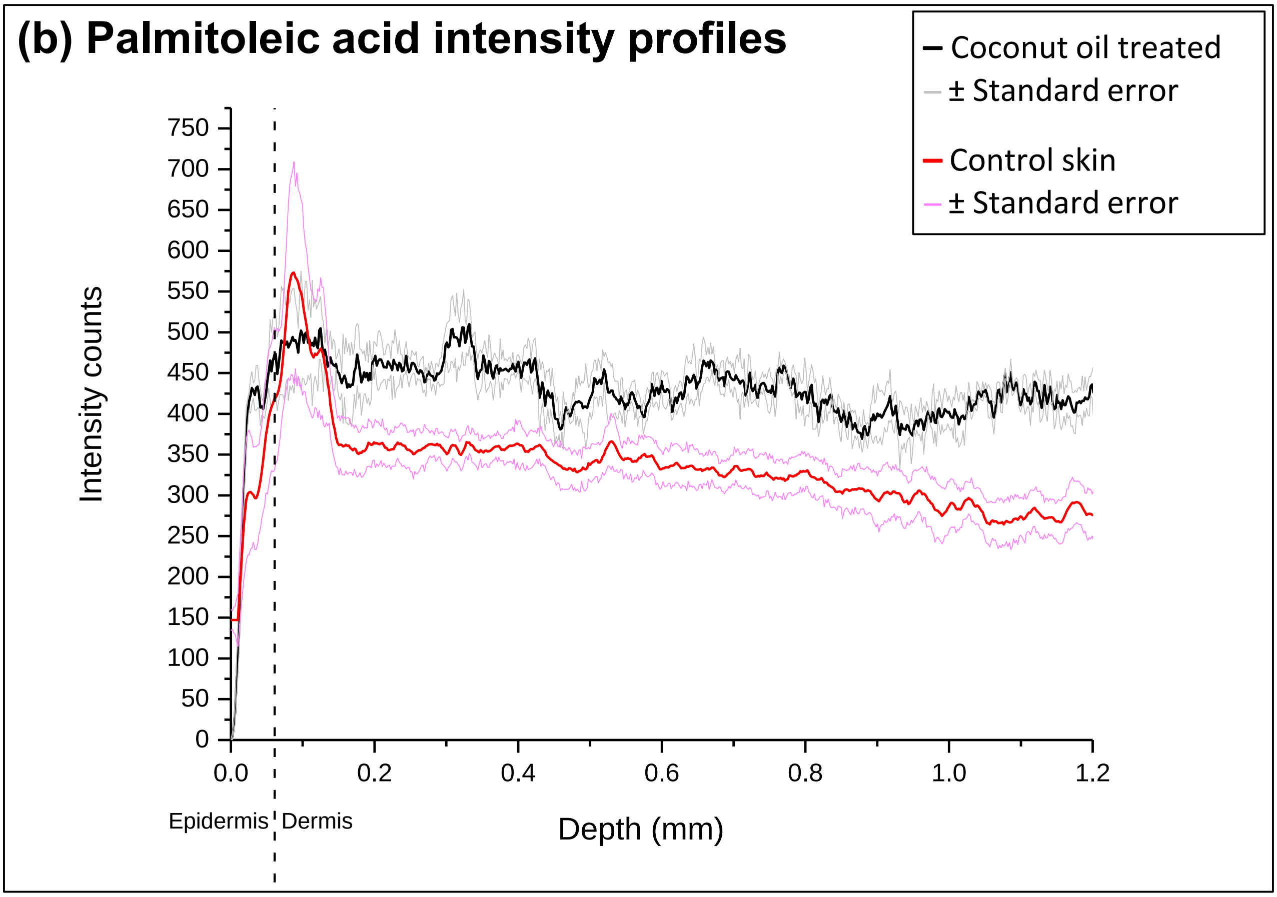

Palmitoleic acid ion intensity profile as the function of depth in coconut oil treated skin samples and compared to the profile obtained from the control skin samples.

Black line corresponds to the average ion intensity values obtained from integrated ion images of skin samples treated with coconut oil; red line corresponds to the profile obtained in control skin samples.

Palmitic acid intensity profiles

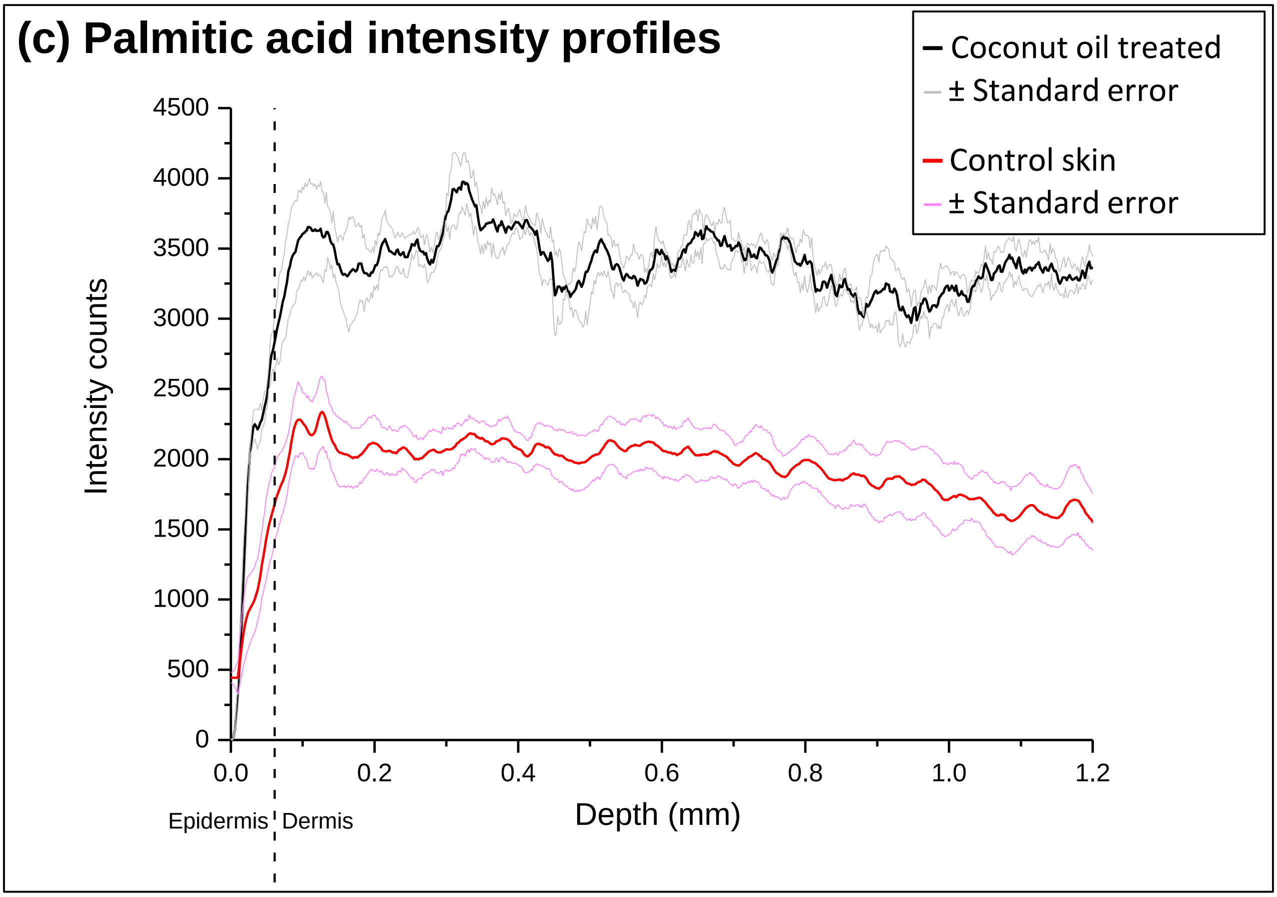

Palmitic acid ion intensity profile as the function of depth in coconut oil treated skin samples and compared to the profile obtained from the control skin samples.

Black line corresponds to the average ion intensity values obtained from integrated ion images of skin samples treated with coconut oil; red line corresponds to the profile obtained in control skin samples.

Oleic acid intensity profiles

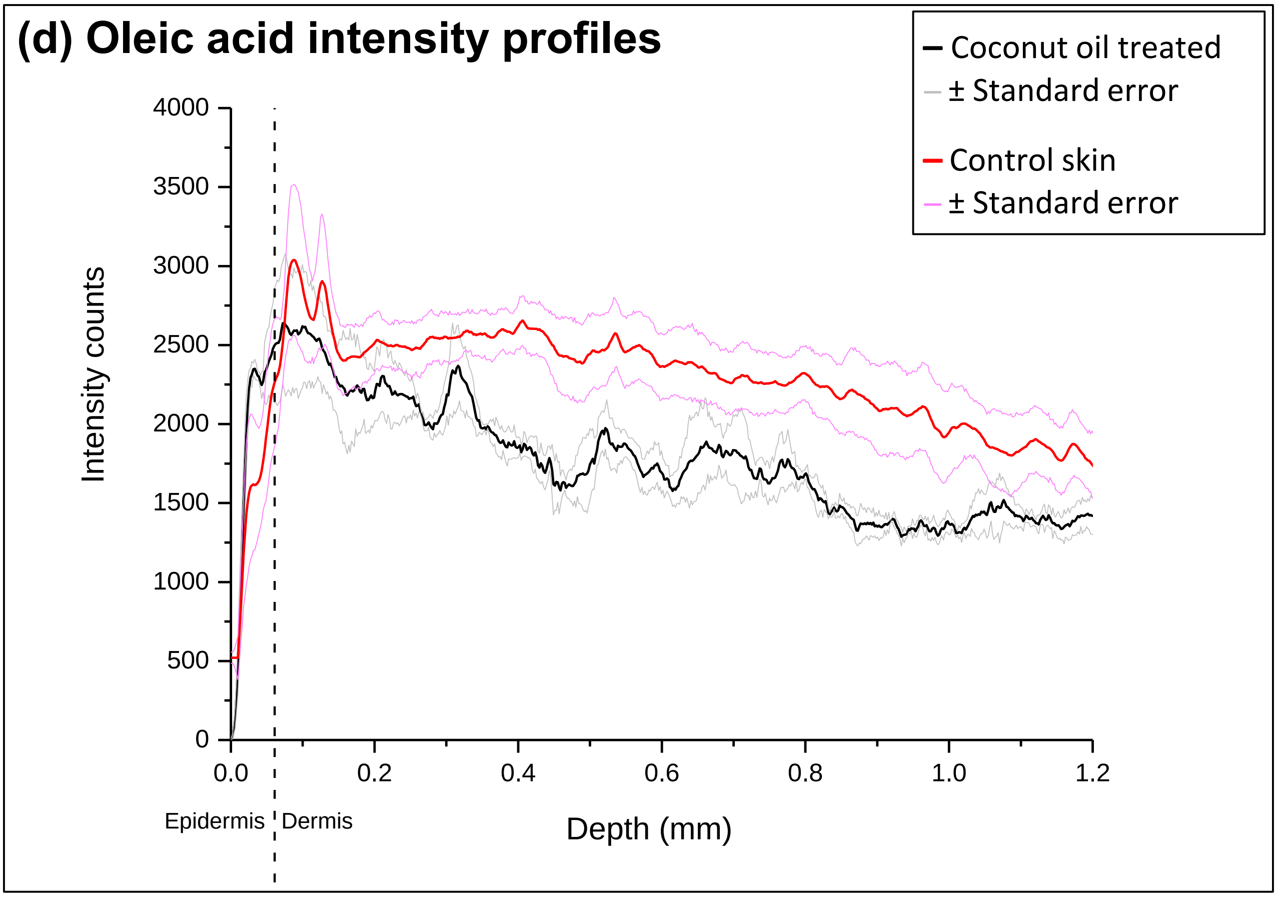

Oleic acid ion intensity profile as the function of depth in coconut oil treated skin samples and compared to the profile obtained from the control skin samples. Black line corresponds to the average ion intensity values obtained from integrated ion images of skin samples treated with coconut oil; red line corresponds to the profile obtained in control skin samples.

Stearic acid intensity profiles

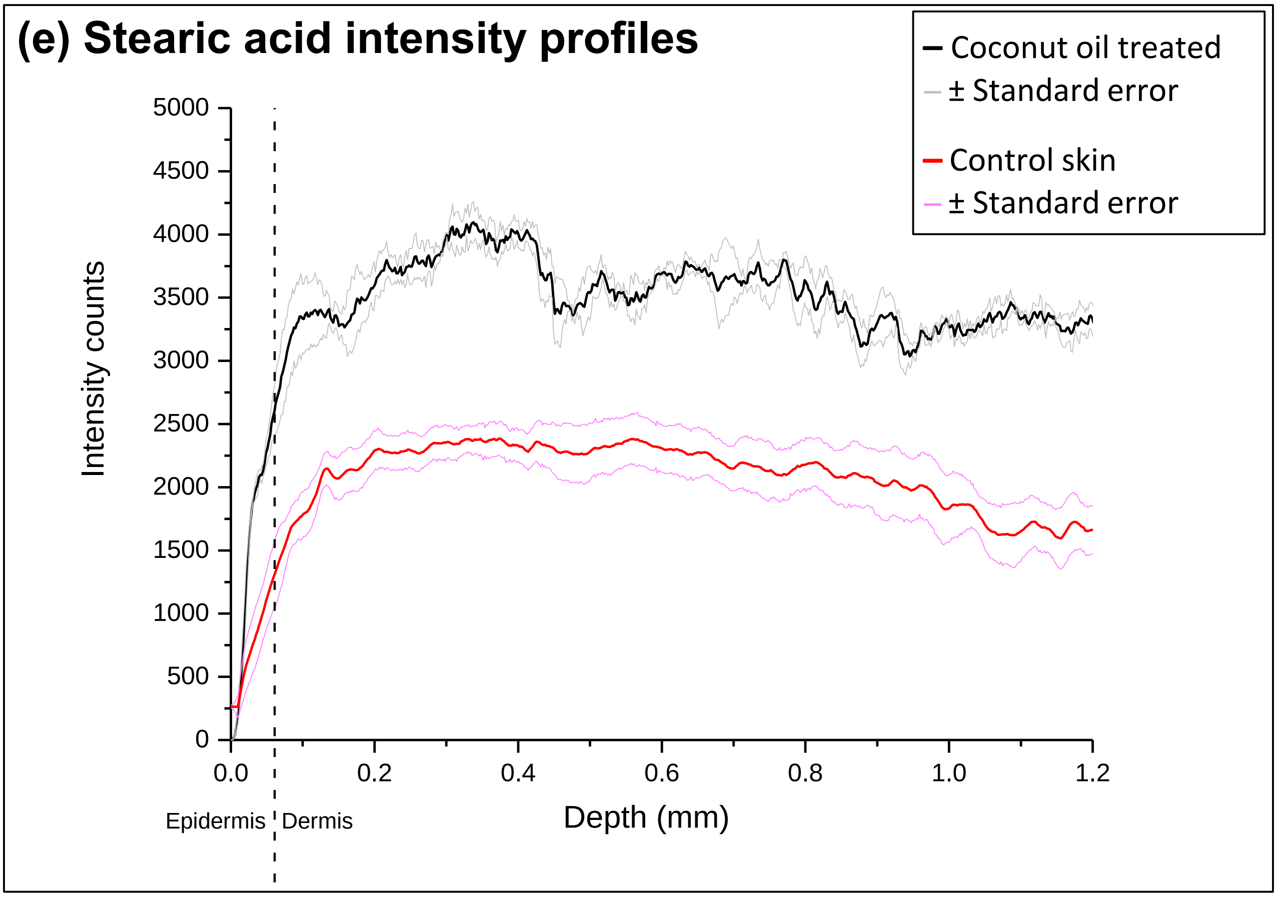

Stearic acid ion intensity profile as the function of depth in coconut oil treated skin samples and compared to the profile obtained from the control skin samples.
Black line corresponds to the average ion intensity values obtained from integrated ion images of skin samples treated with coconut oil; red line corresponds to the profile obtained in control skin samples.

Ion intensity profiles of fatty acids in the skin samples treated with olive oil

Linoleic acid intensity profiles

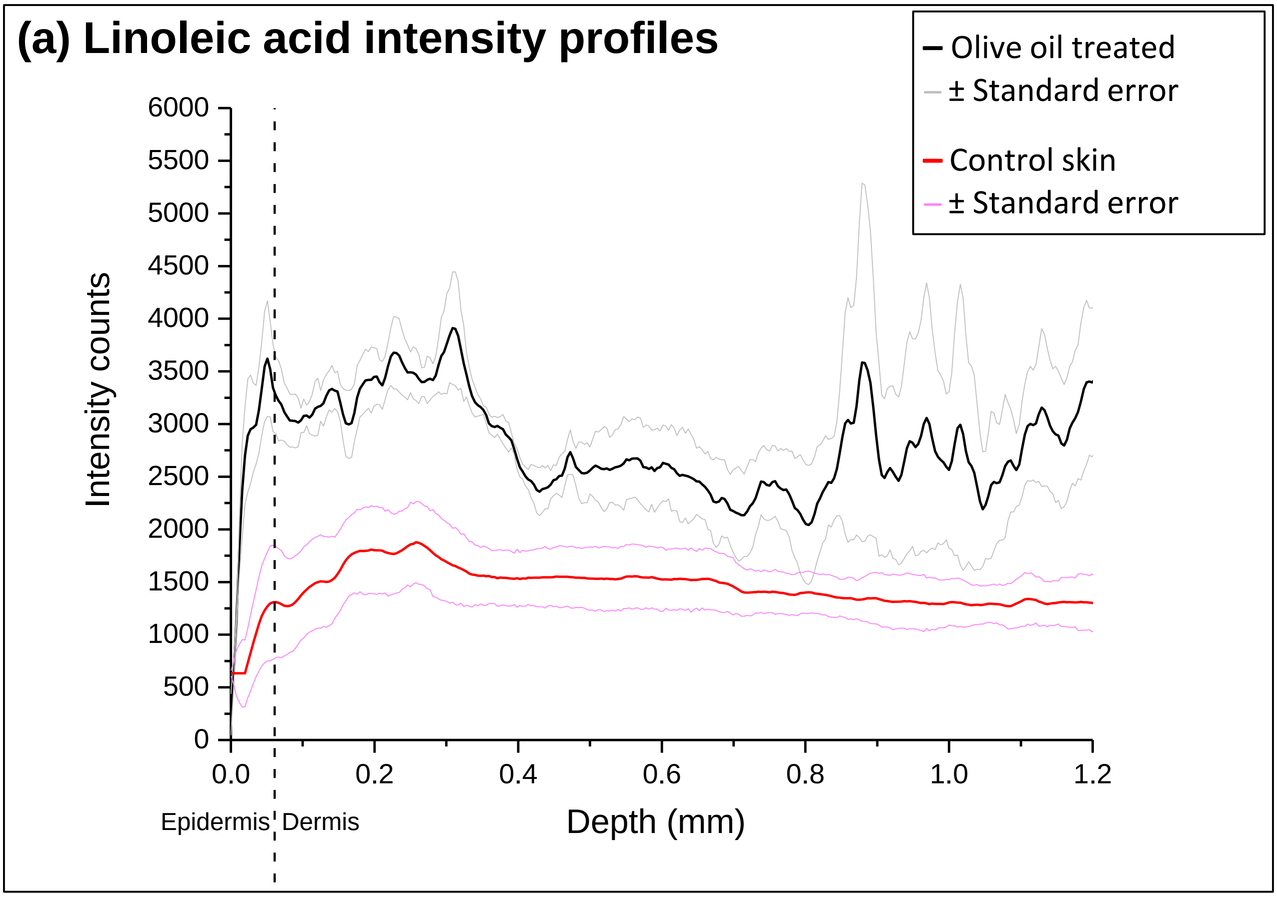

Linoleic acid ion intensity profile as the function of depth in olive oil treated skin samples and compared to the profile obtained from the control skin samples. Black line corresponds to the average ion intensity values obtained from integrated ion images of skin samples treated with olive oil; red line corresponds to the profile obtained in control skin samples.

Palmitoleic acid intensity profiles

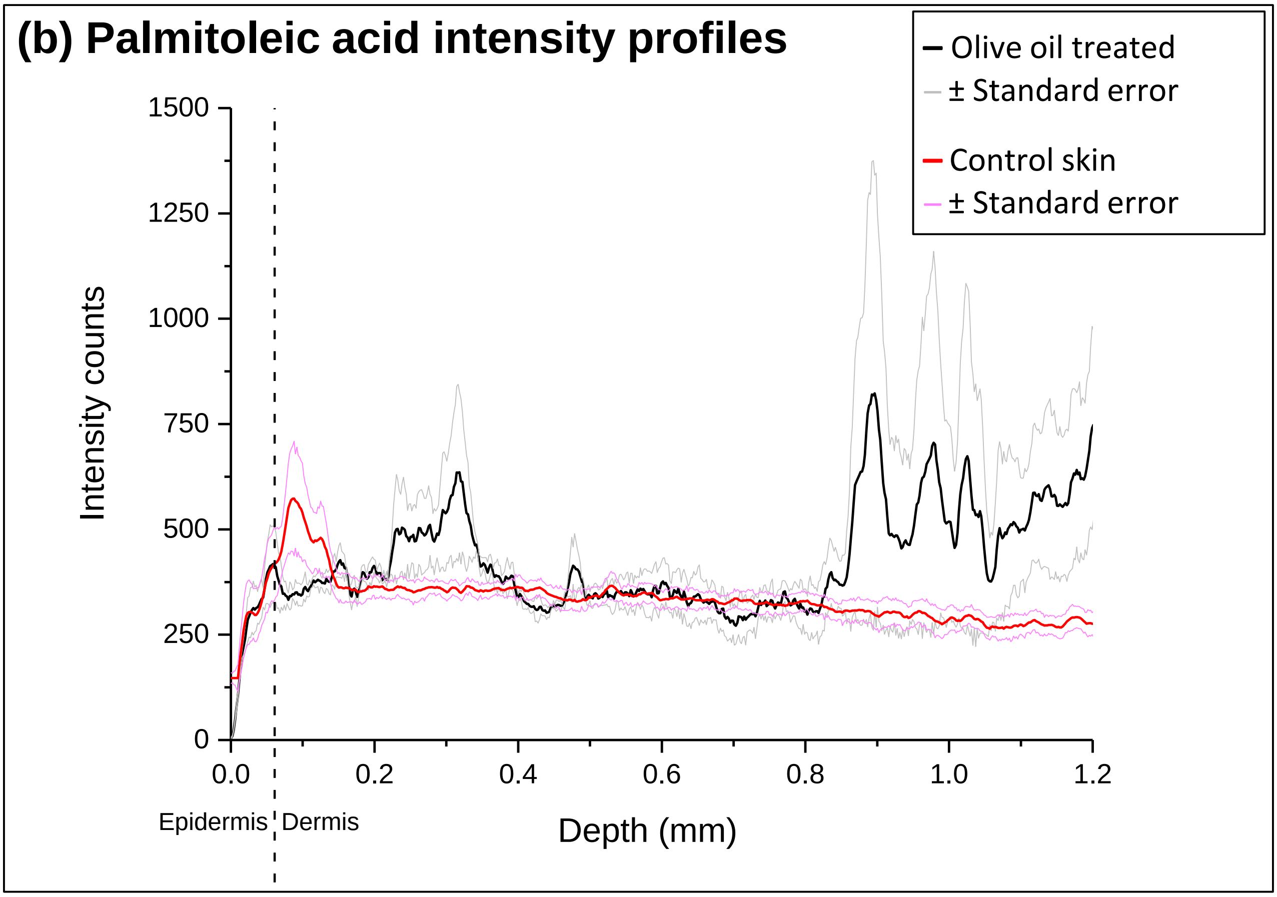

Palmitoleic acid ion intensity profile as the function of depth in olive oil treated skin samples and compared to the profile obtained from the control skin samples.

Black line corresponds to the average ion intensity values obtained from integrated ion images of skin samples treated with olive oil; red line corresponds to the profile obtained in control skin samples.

Palmitic acid intensity profiles

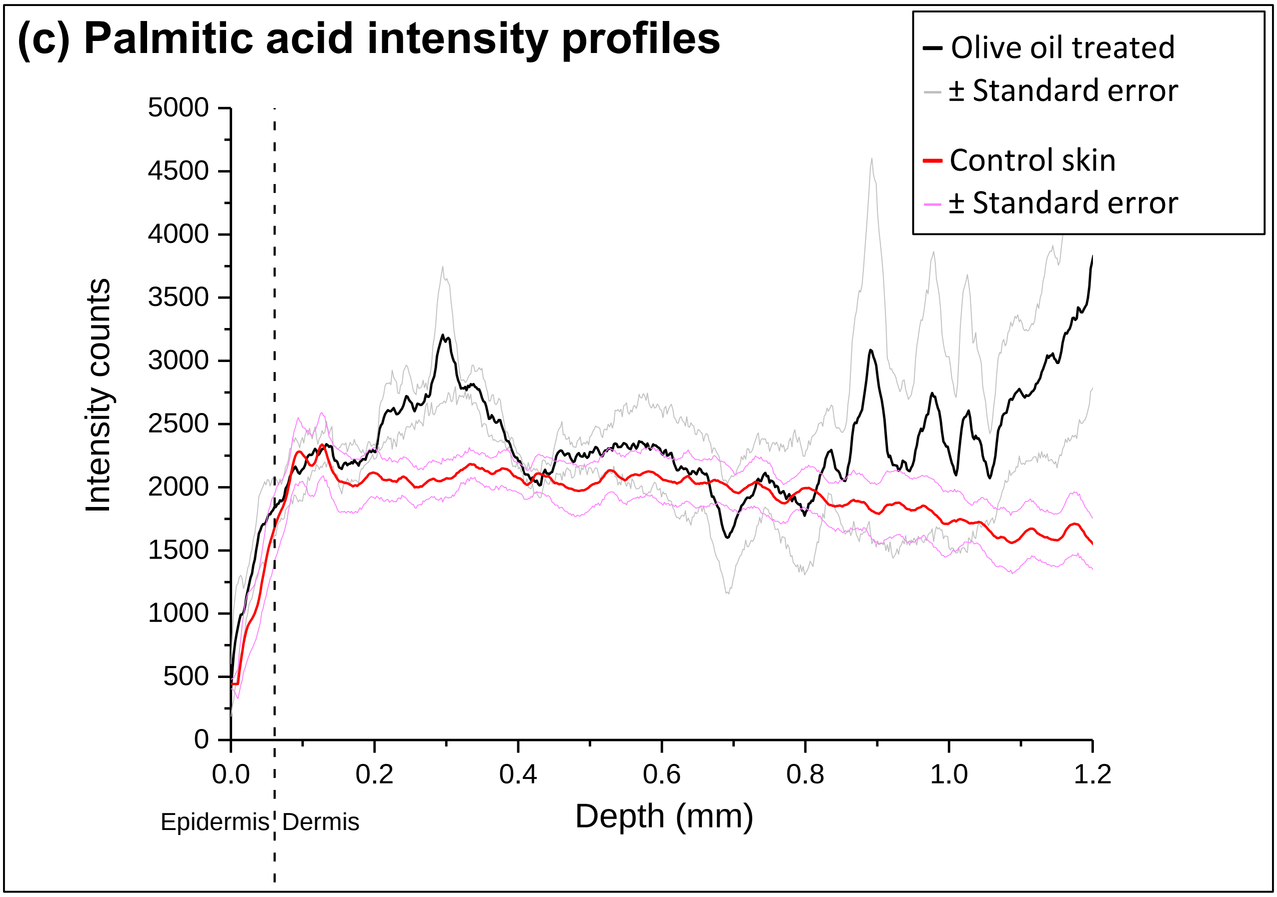

Palmitic acid ion intensity profile as the function of depth in olive oil treated skin samples and compared to the profile obtained from the control skin samples. Black line corresponds to the average ion intensity values obtained from integrated ion images of skin samples treated with olive oil; red line corresponds to the profile obtained in control skin samples.

Oleic acid intensity profiles

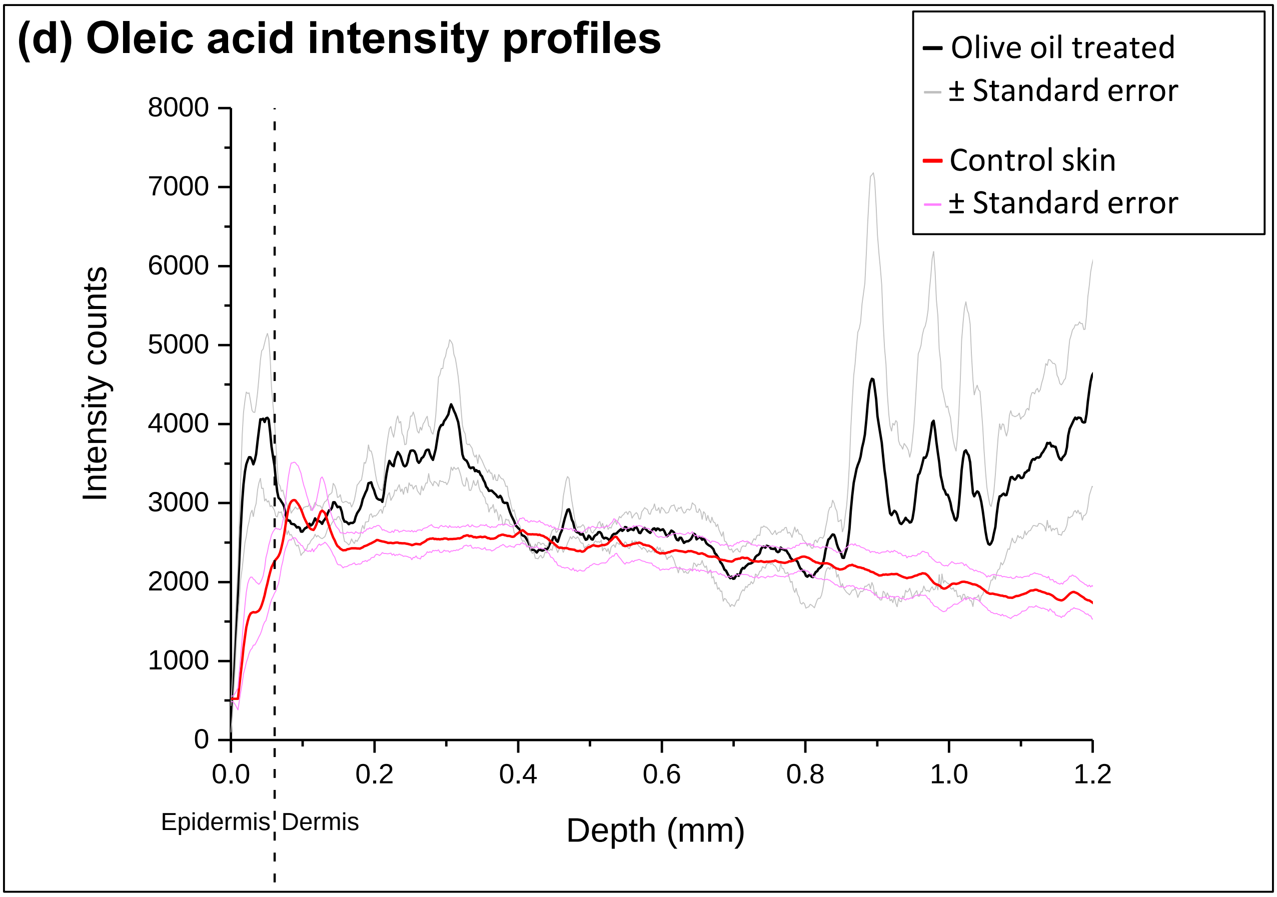

Oleic acid ion intensity profile as the function of depth in olive oil treated skin samples and compared to the profile obtained from the control skin samples. Black line corresponds to the average ion intensity values obtained from integrated ion images of skin samples treated with olive oil; red line corresponds to the profile obtained in control skin samples.

Stearic acid intensity profiles

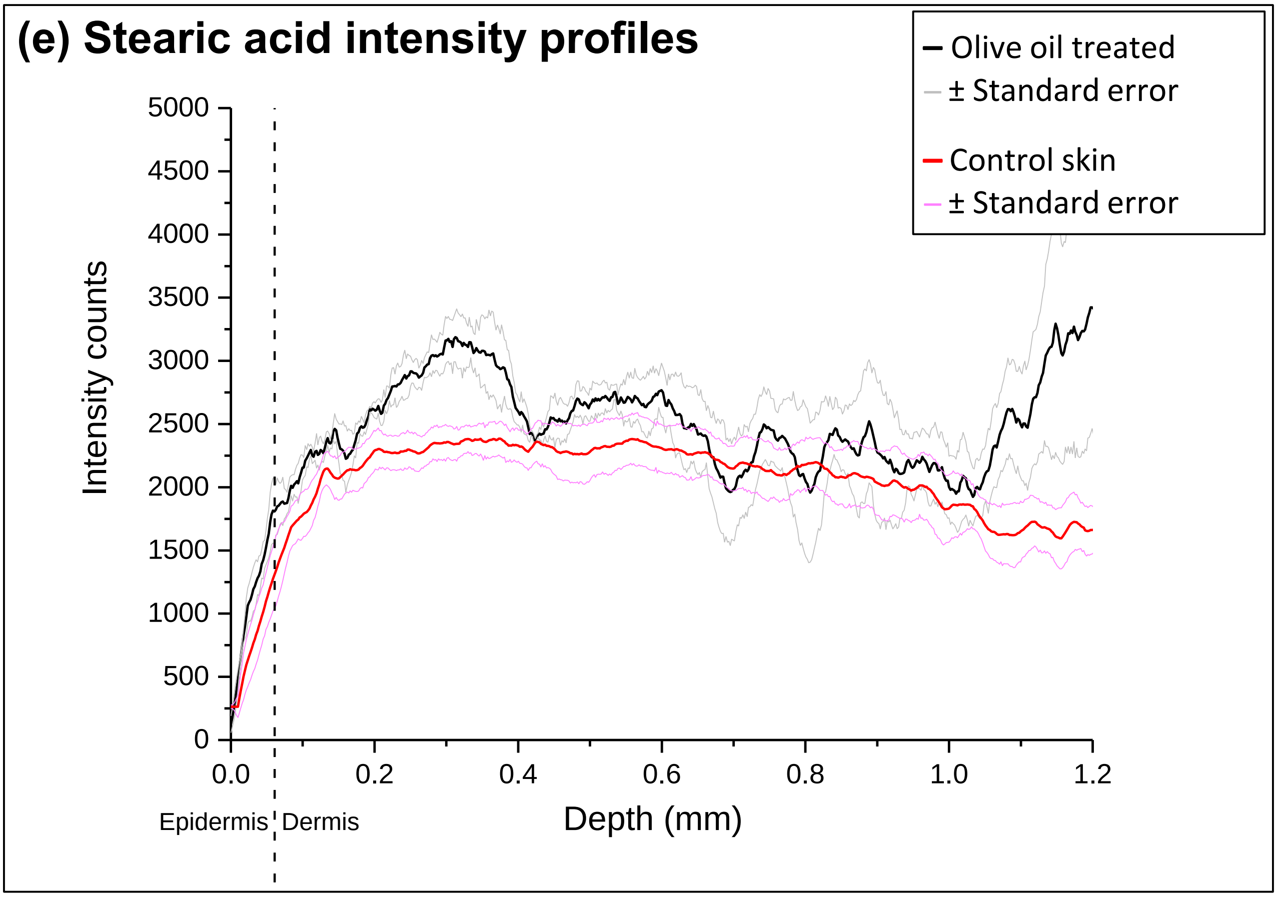

Stearic acid ion intensity profile as the function of depth in olive oil treated skin samples and compared to the profile obtained from the control skin samples. Black line corresponds to the average ion intensity values obtained from integrated ion images of skin samples treated with olive oil; red line corresponds to the profile obtained in control skin samples.

Ion intensity profiles of fatty acids in the skin samples treated with raspberry seed oil

Linoleic acid intensity profiles

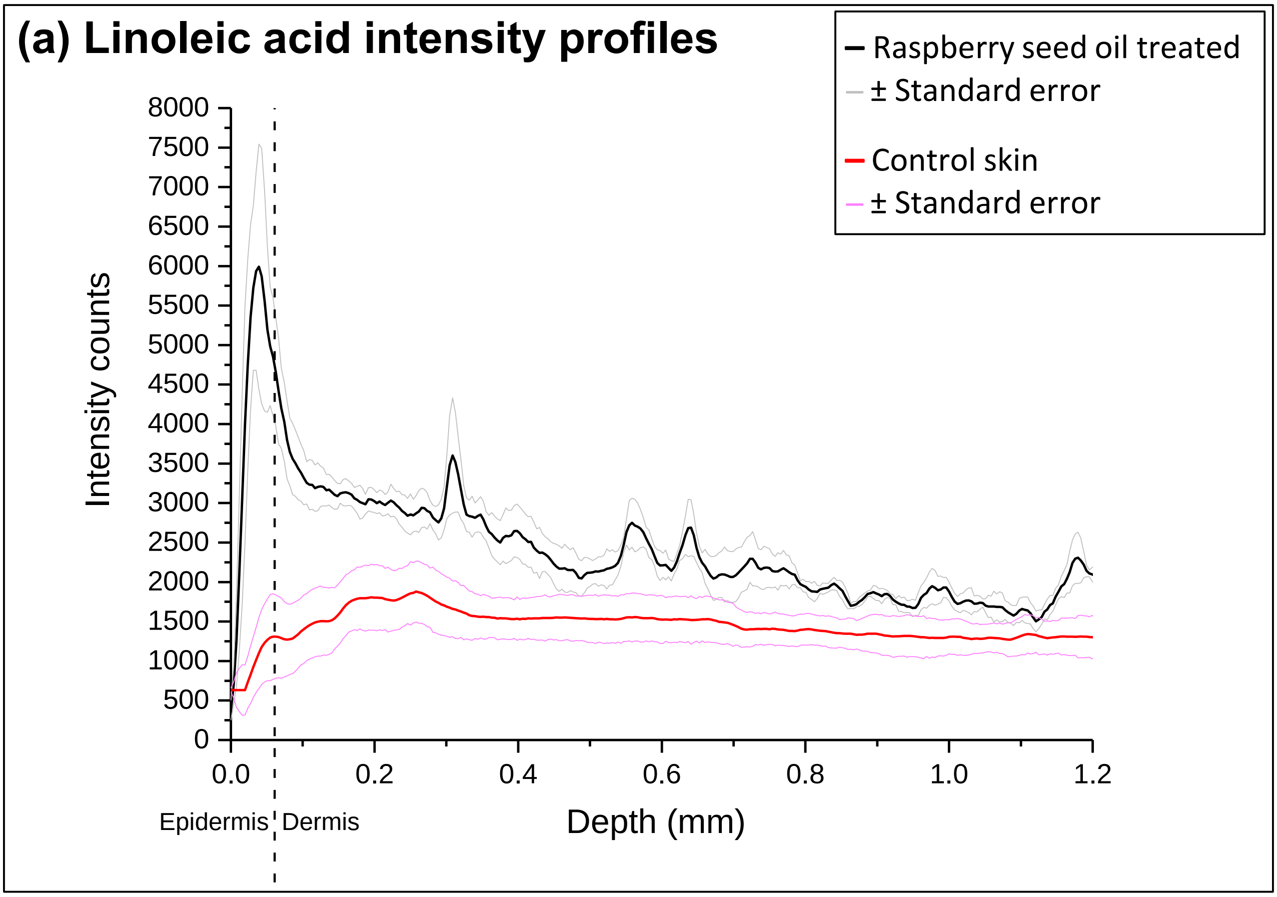

Linoleic acid ion intensity profile as the function of depth in raspberry seed oil treated skin samples and compared to the profile obtained from the control skin samples.

Black line corresponds to the average ion intensity values obtained from integrated ion images of skin samples treated with raspberry seed oil; red line corresponds to the profile obtained in control skin samples.

Palmitoleic acid intensity profiles

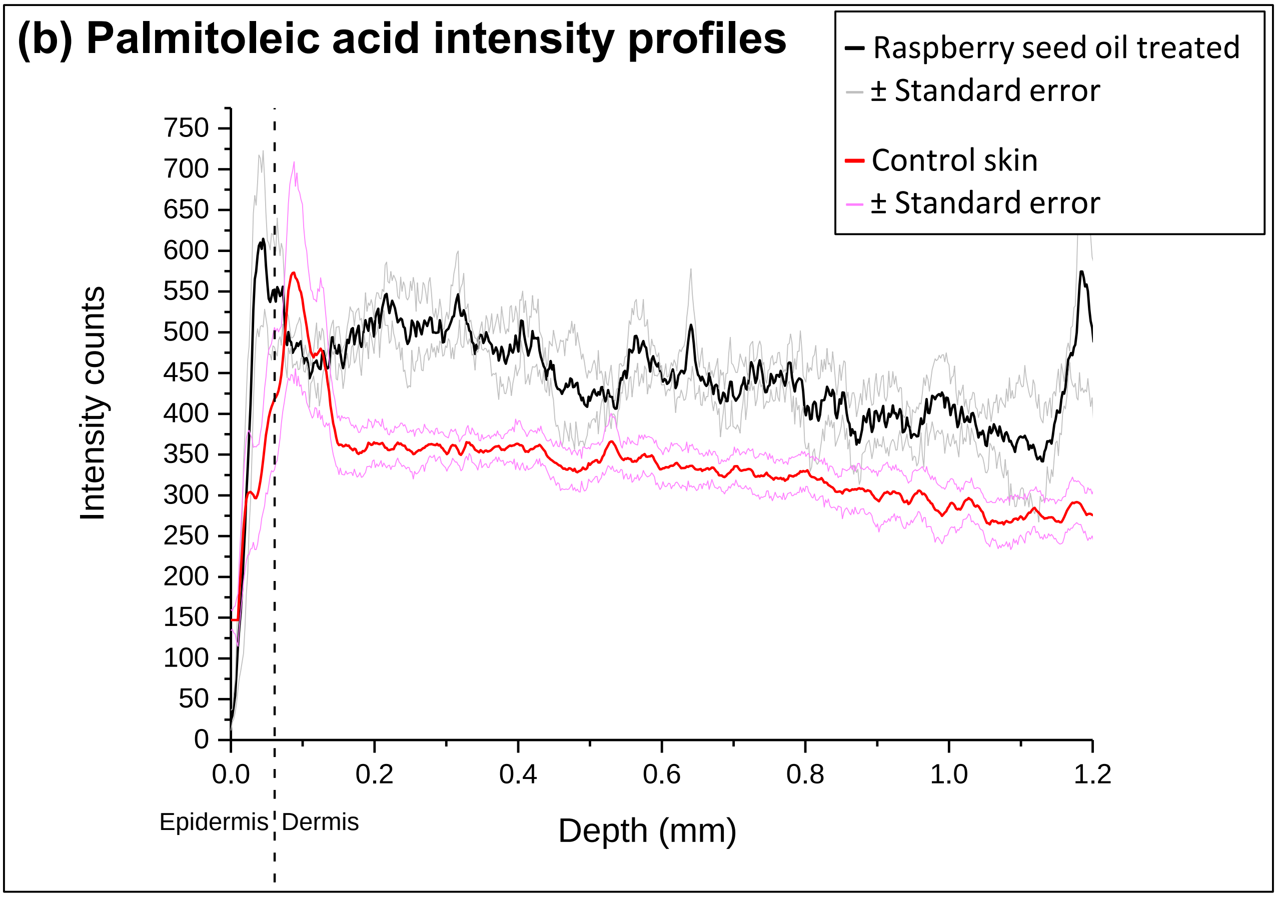

Palmitoleic acid ion intensity profile as the function of depth in raspberry seed oil treated skin samples and compared to the profile obtained from the control skin samples.

Black line corresponds to the average ion intensity values obtained from integrated ion images of skin samples treated with raspberry seed oil; red line corresponds to the profile obtained in control skin samples.

Palmitic acid intensity profiles

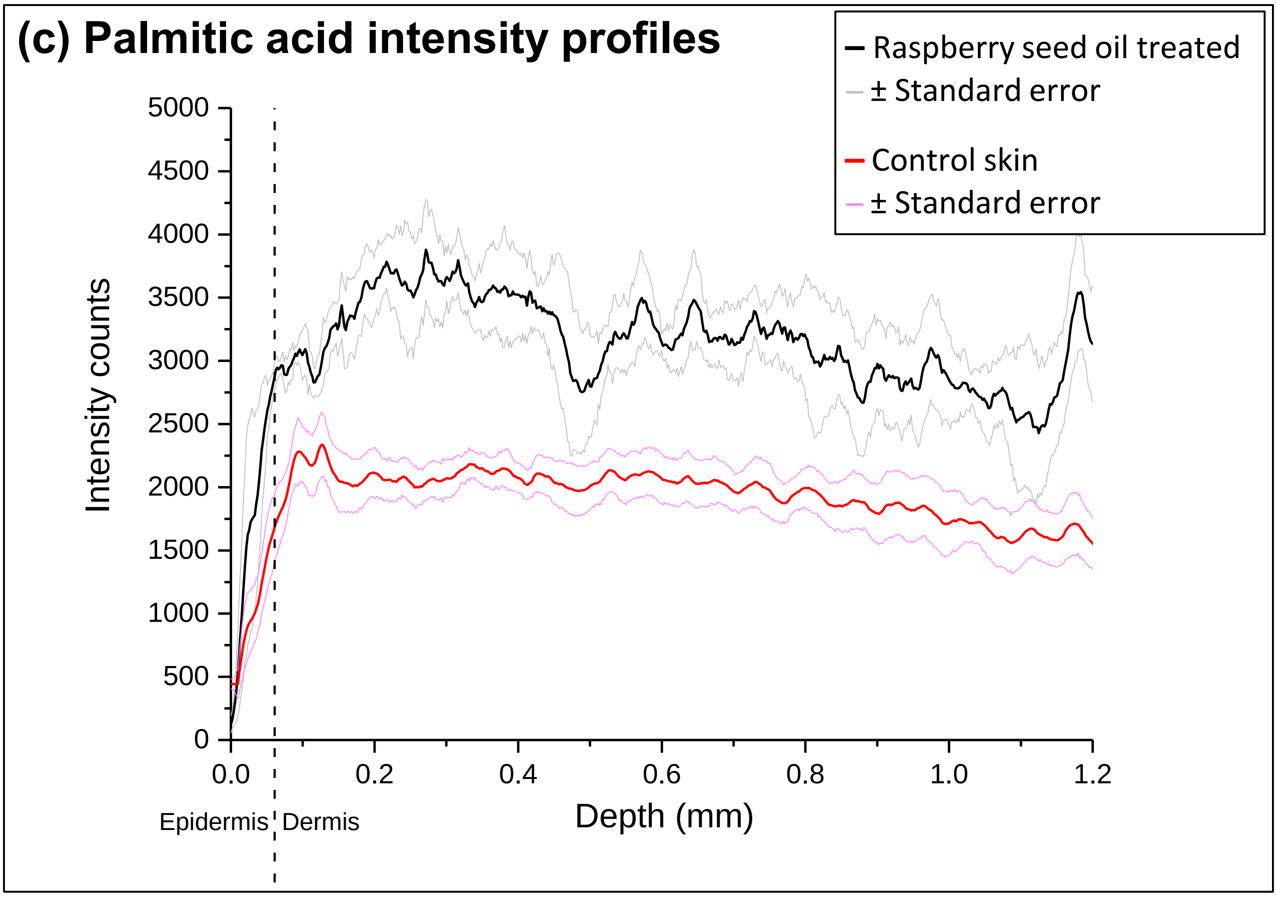

Palmitic acid ion intensity profile as the function of depth in raspberry seed oil treated skin samples and compared to the profile obtained from the control skin samples.

Black line corresponds to the average ion intensity values obtained from integrated ion images of skin samples treated with raspberry seed oil; red line corresponds to the profile obtained in control skin samples.

Oleic acid intensity profiles

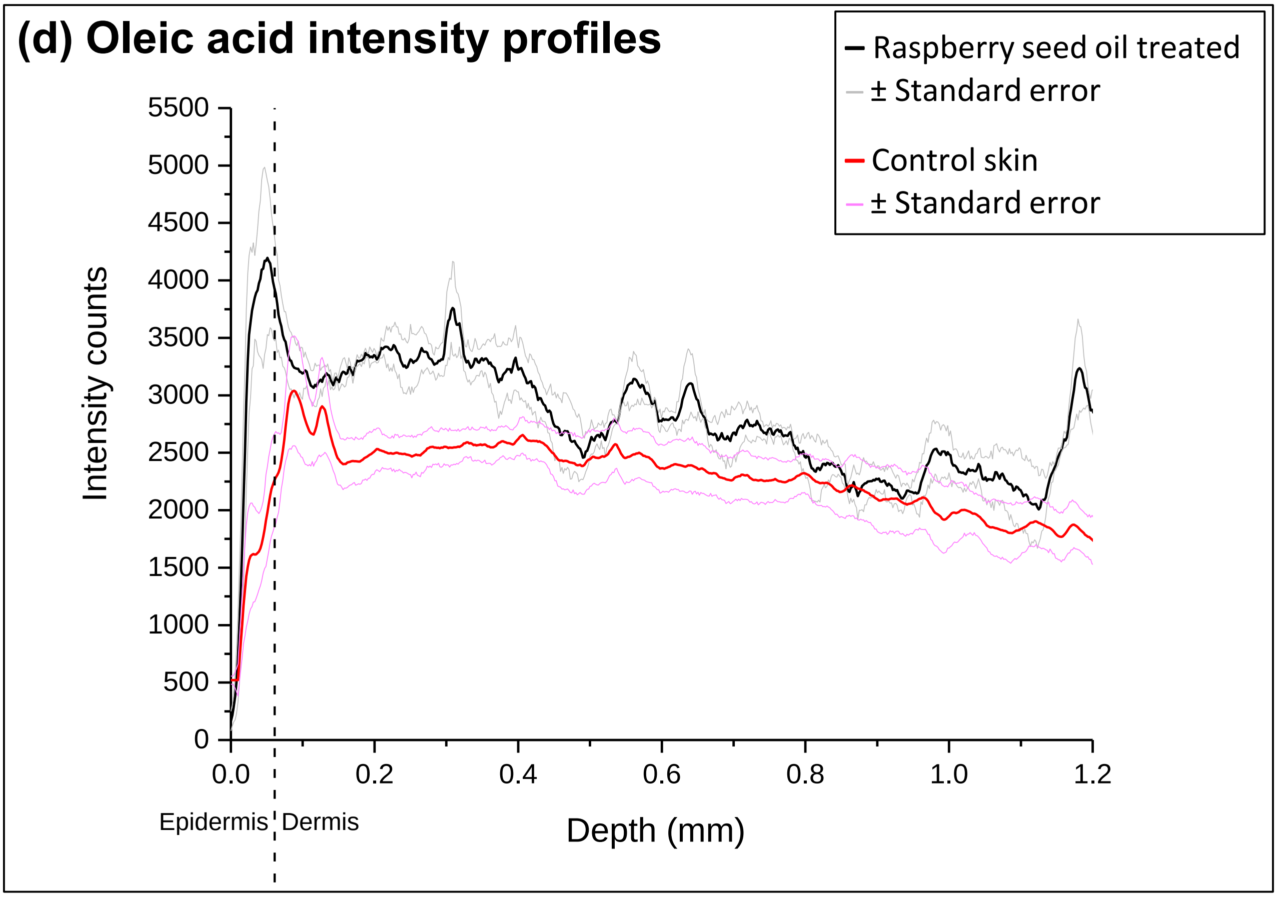

Oleic acid ion intensity profile as the function of depth in raspberry seed oil treated skin samples and compared to the profile obtained from the control skin samples.

Black line corresponds to the average ion intensity values obtained from integrated ion images of skin samples treated with raspberry seed oil; red line corresponds to the profile obtained in control skin samples.

Stearic acid intensity profiles

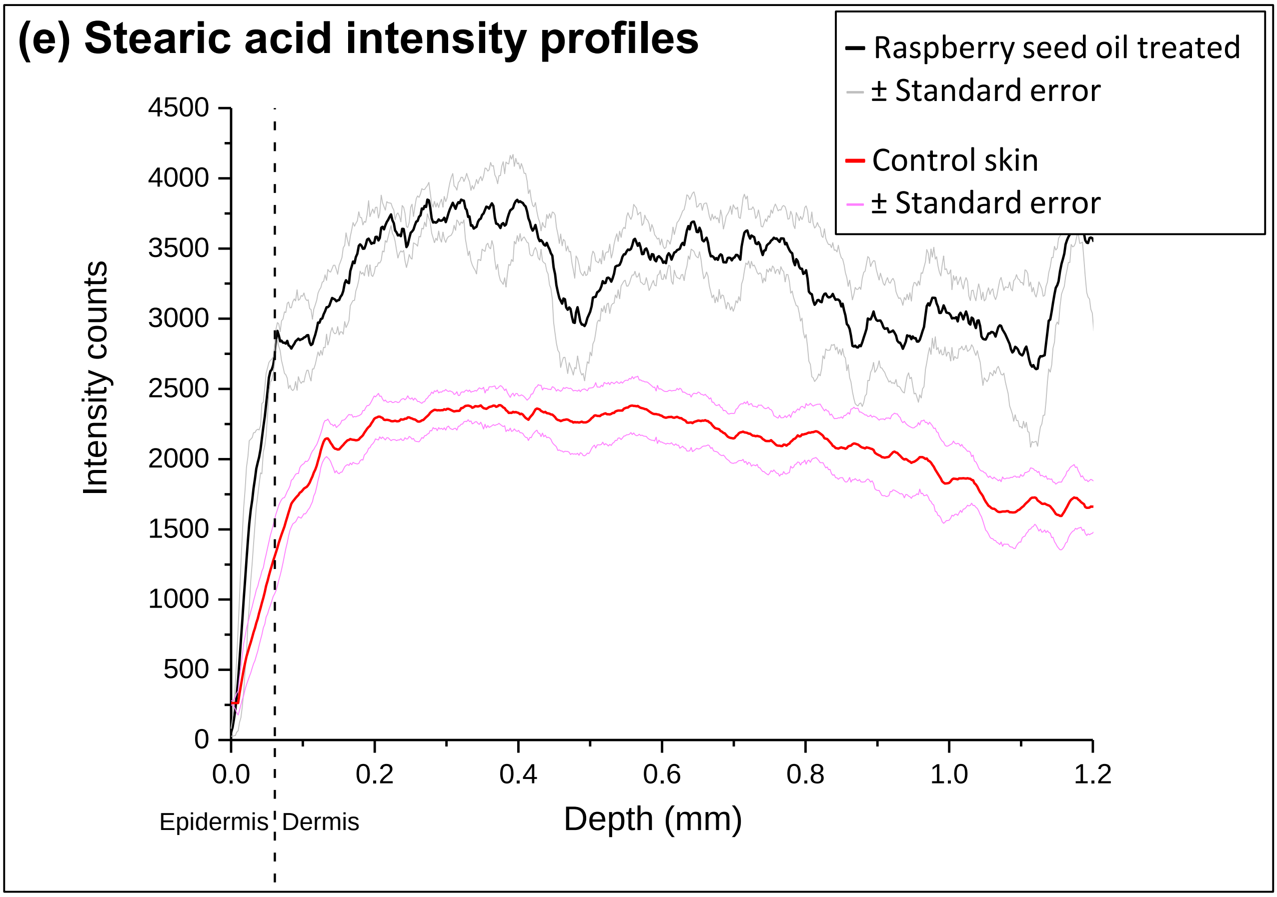

Stearic acid ion intensity profile as the function of depth in raspberry seed oil treated skin samples and compared to the profile obtained from the control skin samples.

Black line corresponds to the average ion intensity values obtained from integrated ion images of skin samples treated with raspberry seed oil; red line corresponds to the profile obtained in control skin samples.

Ion intensity profiles of fatty acids in the skin samples treated with sea-buckthorn pulp oil

Linoleic acid intensity profiles

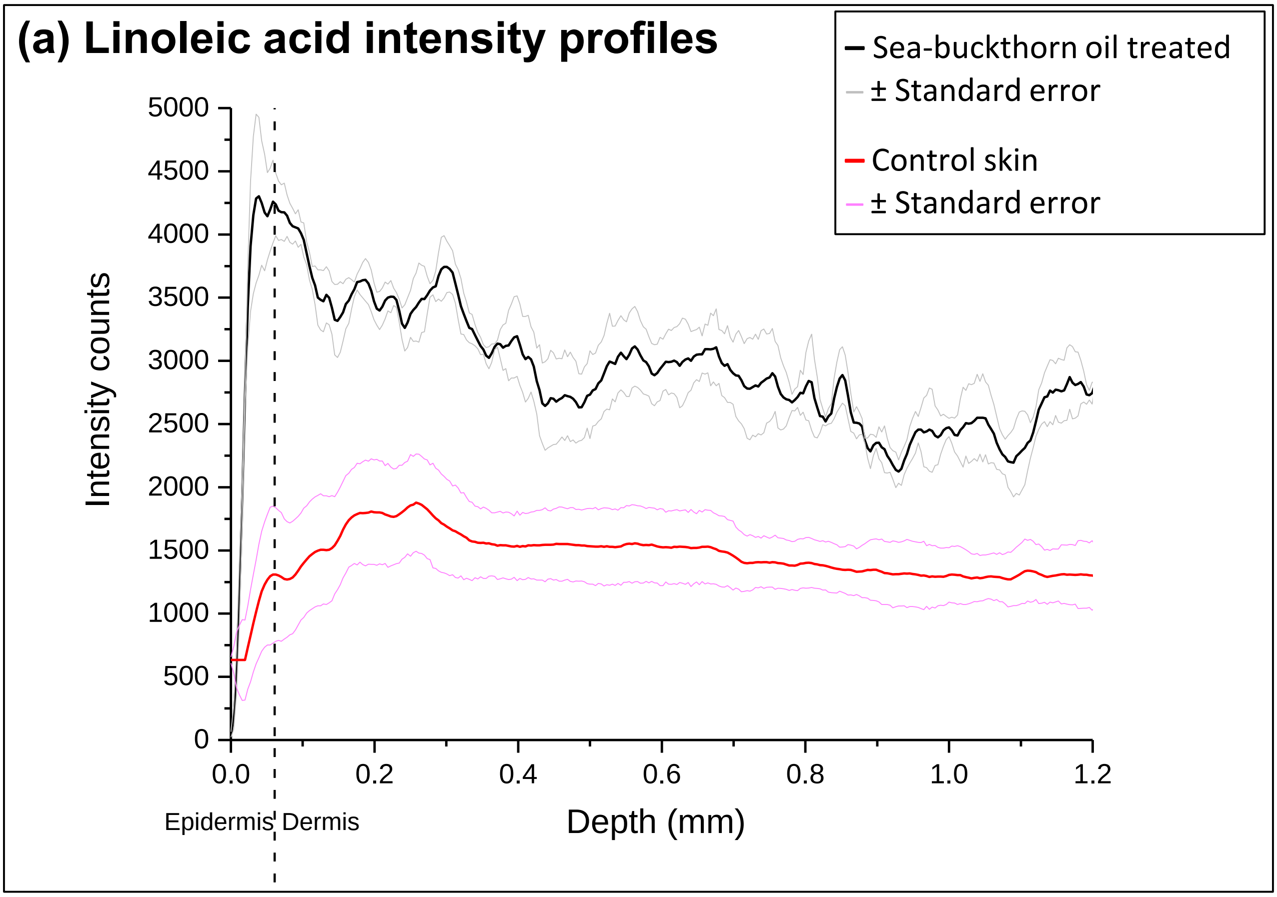

Linoleic acid ion intensity profile as the function of depth in sea-buckthorn pulp oil treated skin samples and compared to the profile obtained from the control skin samples.

Black line corresponds to the average ion intensity values obtained from integrated ion images of skin samples treated with sea-buckthorn pulp oil; red line corresponds to the profile obtained in control skin samples.

Palmitoleic acid intensity profiles

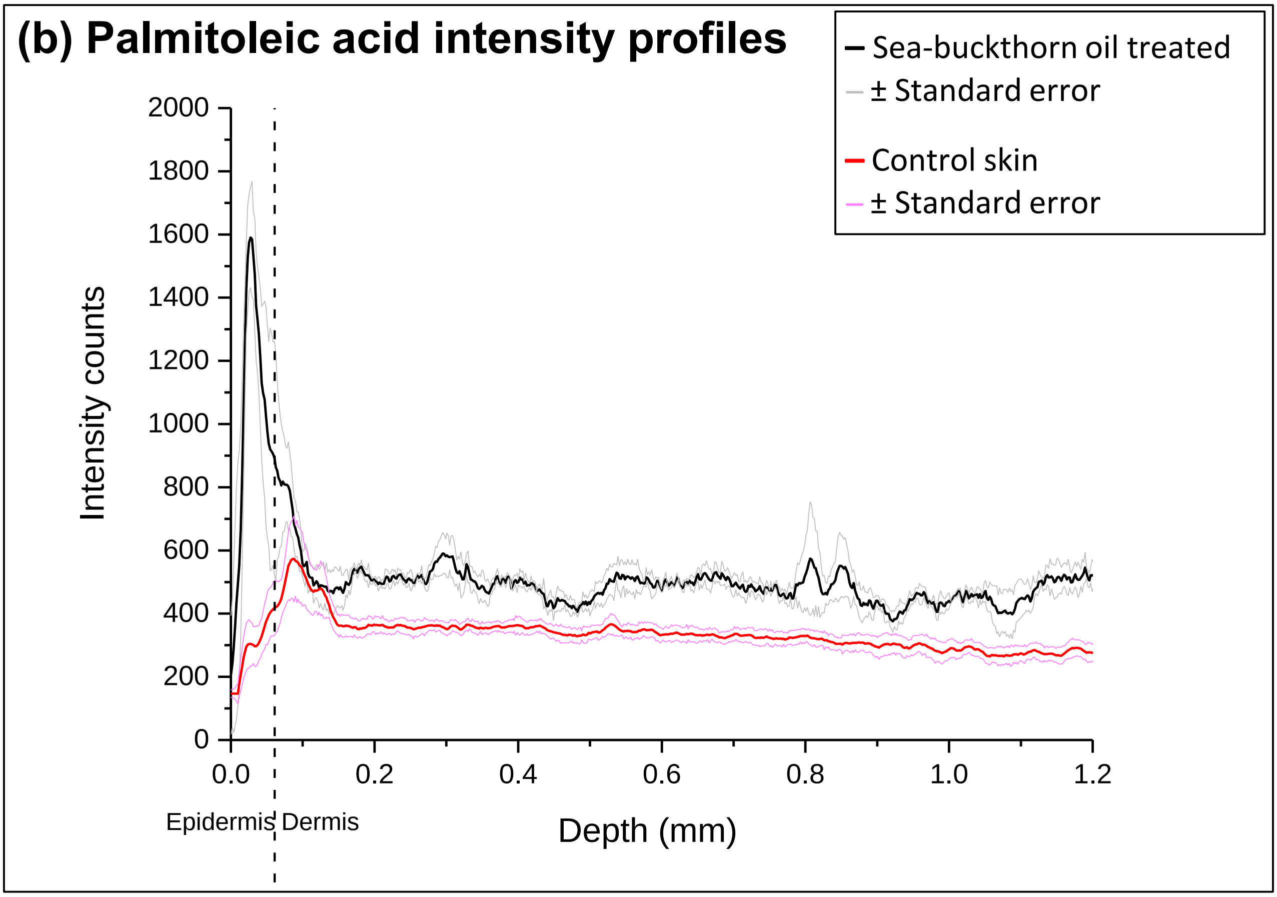

Palmitoleic acid ion intensity profile as the function of depth in sea-buckthorn pulp oil treated skin samples and compared to the profile obtained from the control skin samples.

Black line corresponds to the average ion intensity values obtained from integrated ion images of skin samples treated with sea-buckthorn pulp oil; red line corresponds to the profile obtained in control skin samples.

Palmitic acid intensity profiles

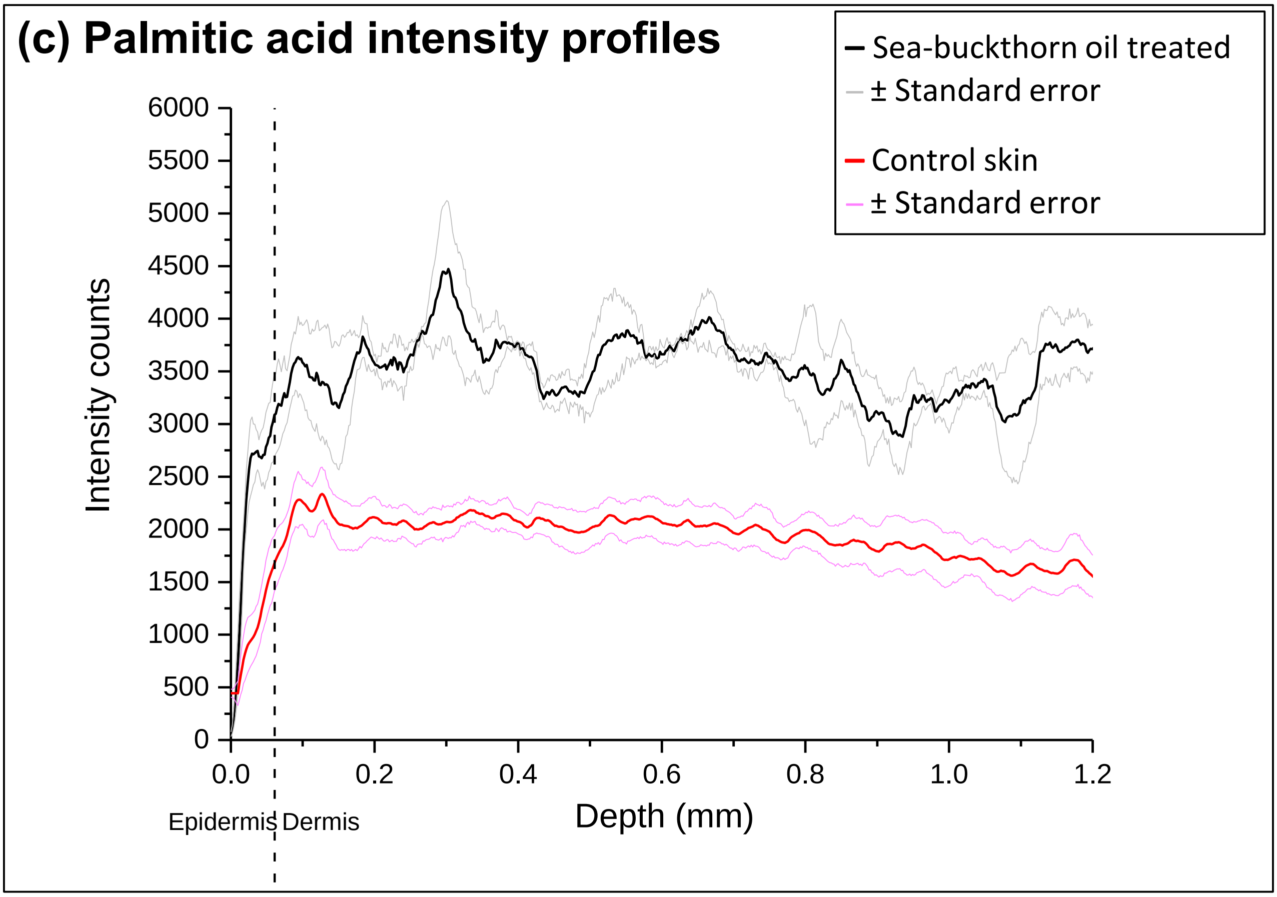

Palmitic acid ion intensity profile as the function of depth in sea-buckthorn pulp oil treated skin samples and compared to the profile obtained from the control skin samples.

Black line corresponds to the average ion intensity values obtained from integrated ion images of skin samples treated with sea-buckthorn pulp oil; red line corresponds to the profile obtained in control skin samples.

Oleic acid intensity profiles

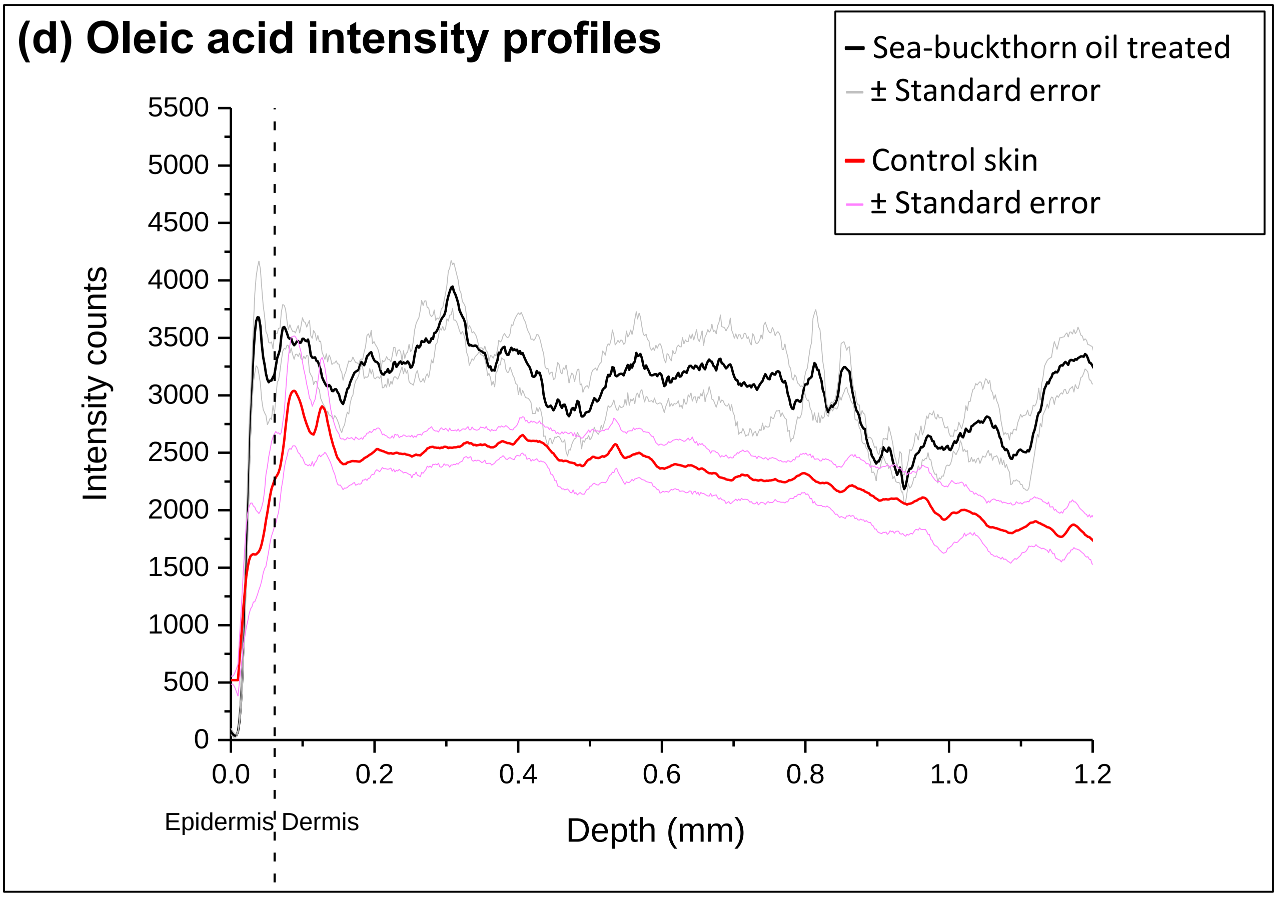

Oleic acid ion intensity profile as the function of depth in sea-buckthorn pulp oil treated skin samples and compared to the profile obtained from the control skin samples.

Black line corresponds to the average ion intensity values obtained from integrated ion images of skin samples treated with sea-buckthorn pulp oil; red line corresponds to the profile obtained in control skin samples.

Stearic acid intensity profiles

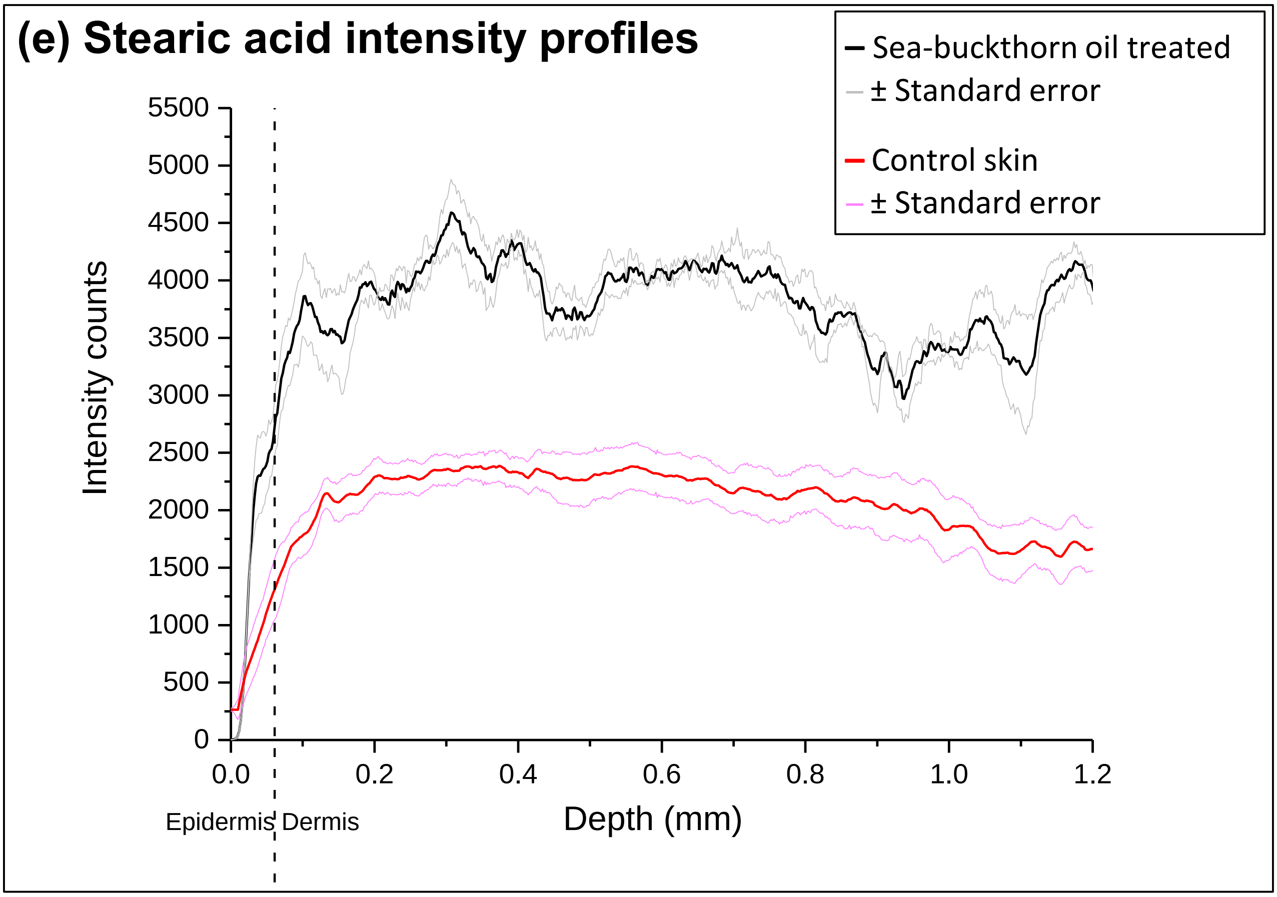

Stearic acid ion intensity profile as the function of depth in sea-buckthorn pulp oil treated skin samples and compared to the profile obtained from the control skin samples.

Black line corresponds to the average ion intensity values obtained from integrated ion images of skin samples treated with sea-buckthorn pulp oil; red line corresponds to the profile obtained in control skin samples.

Ion intensity profiles of fatty acids in the skin samples treated with soybean oil

Linoleic acid intensity profiles

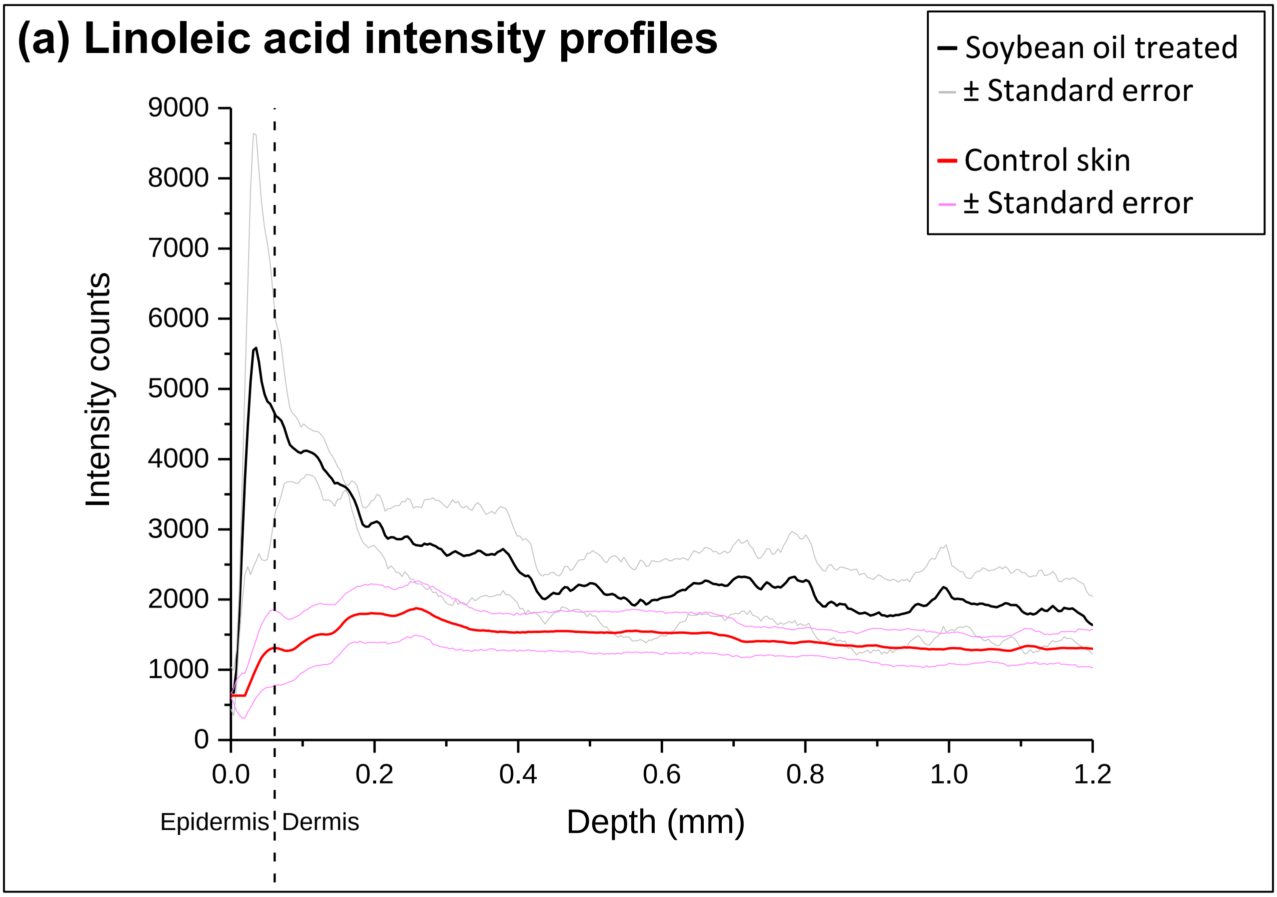

Linoleic acid ion intensity profile as the function of depth in soybean oil treated skin samples and compared to the profile obtained from the control skin samples.

Black line corresponds to the average ion intensity values obtained from integrated ion images of skin samples treated with soybean oil; red line corresponds to the profile obtained in control skin samples.

Palmitoleic acid intensity profiles

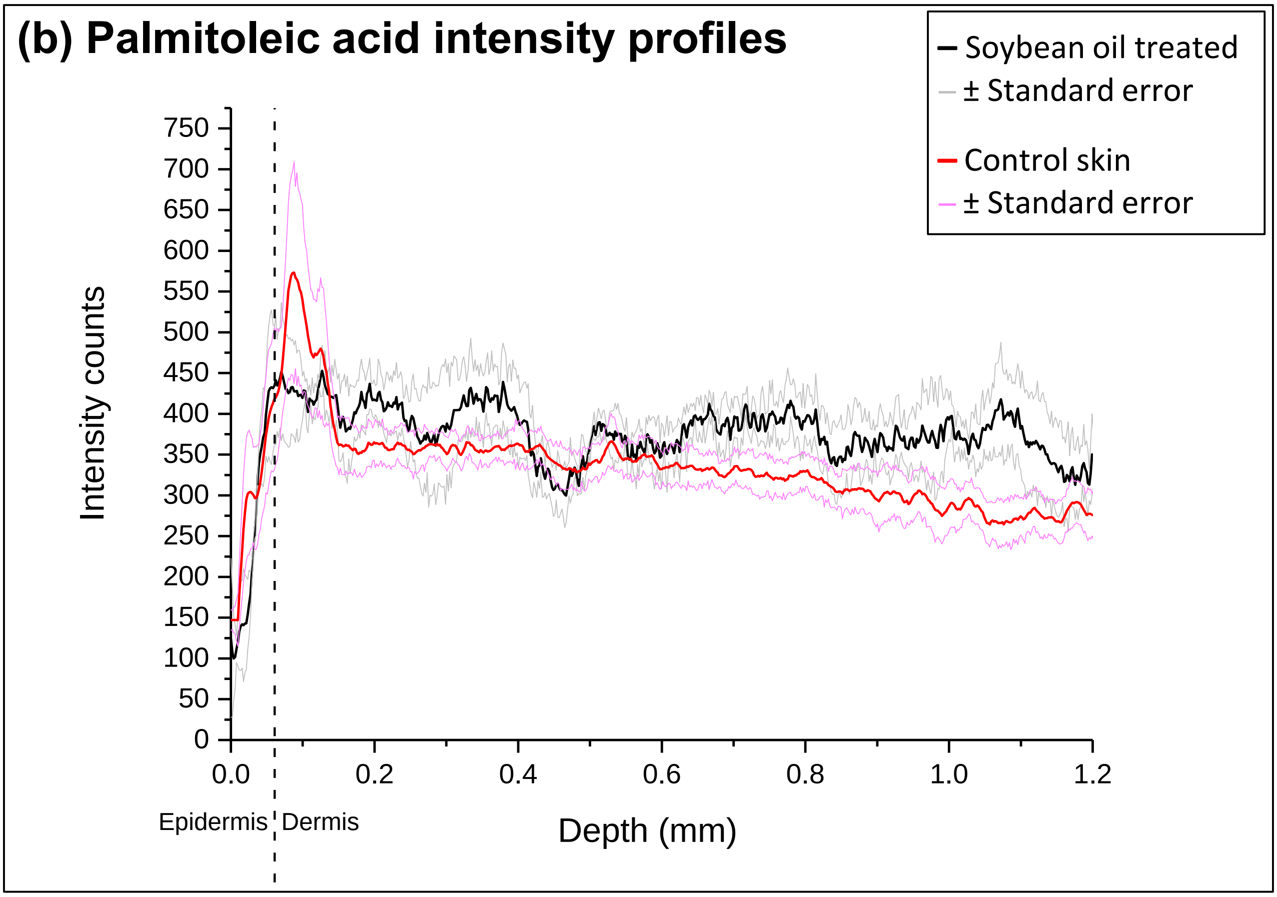

Palmitoleic acid ion intensity profile as the function of depth in soybean oil treated skin samples and compared to the profile obtained from the control skin samples.

Black line corresponds to the average ion intensity values obtained from integrated ion images of skin samples treated with soybean oil; red line corresponds to the profile obtained in control skin samples.

Palmitic acid intensity profiles

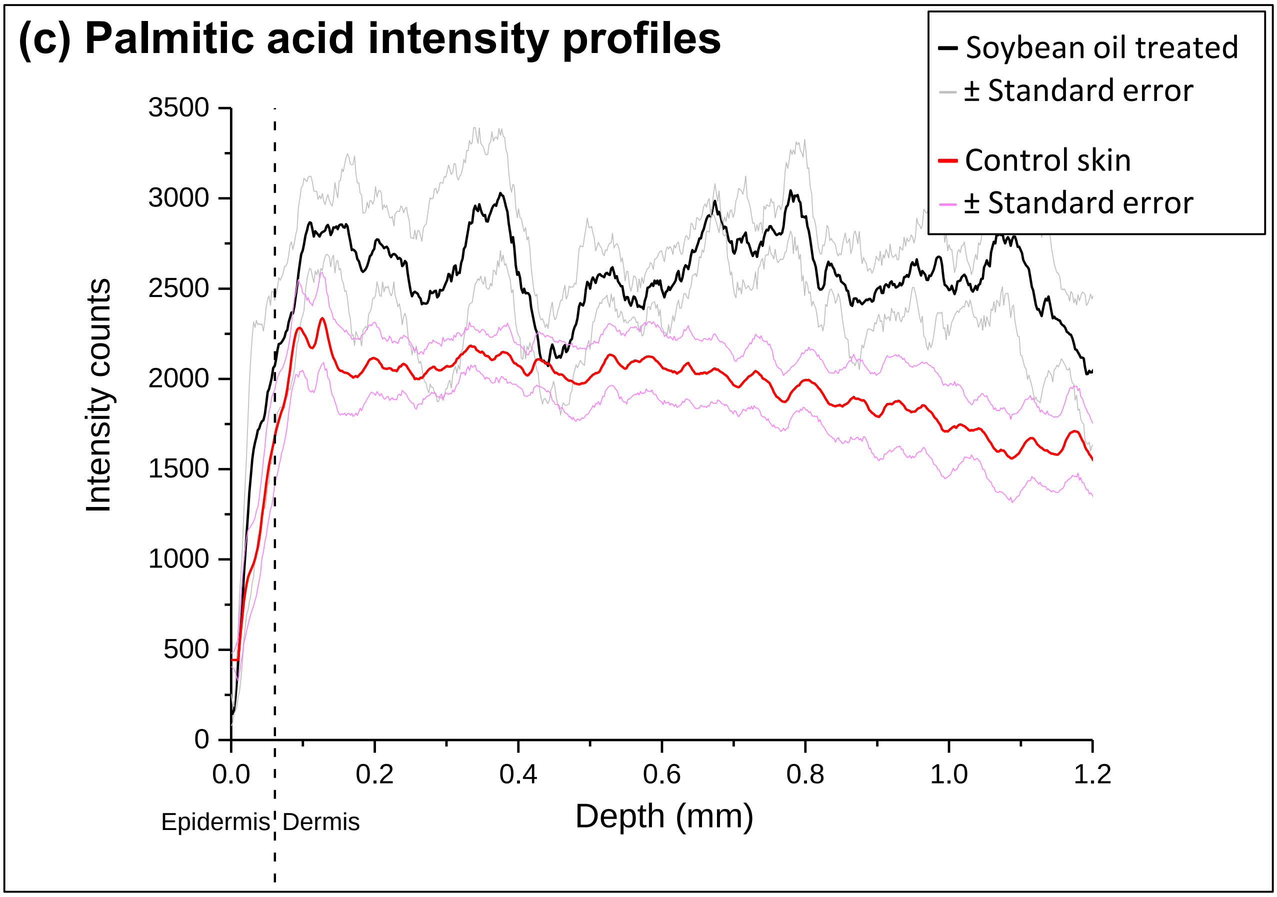

Palmitic acid ion intensity profile as the function of depth in soybean oil treated skin samples and compared to the profile obtained from the control skin samples.

Black line corresponds to the average ion intensity values obtained from integrated ion images of skin samples treated with soybean oil; red line corresponds to the profile obtained in control skin samples.

Oleic acid intensity profiles

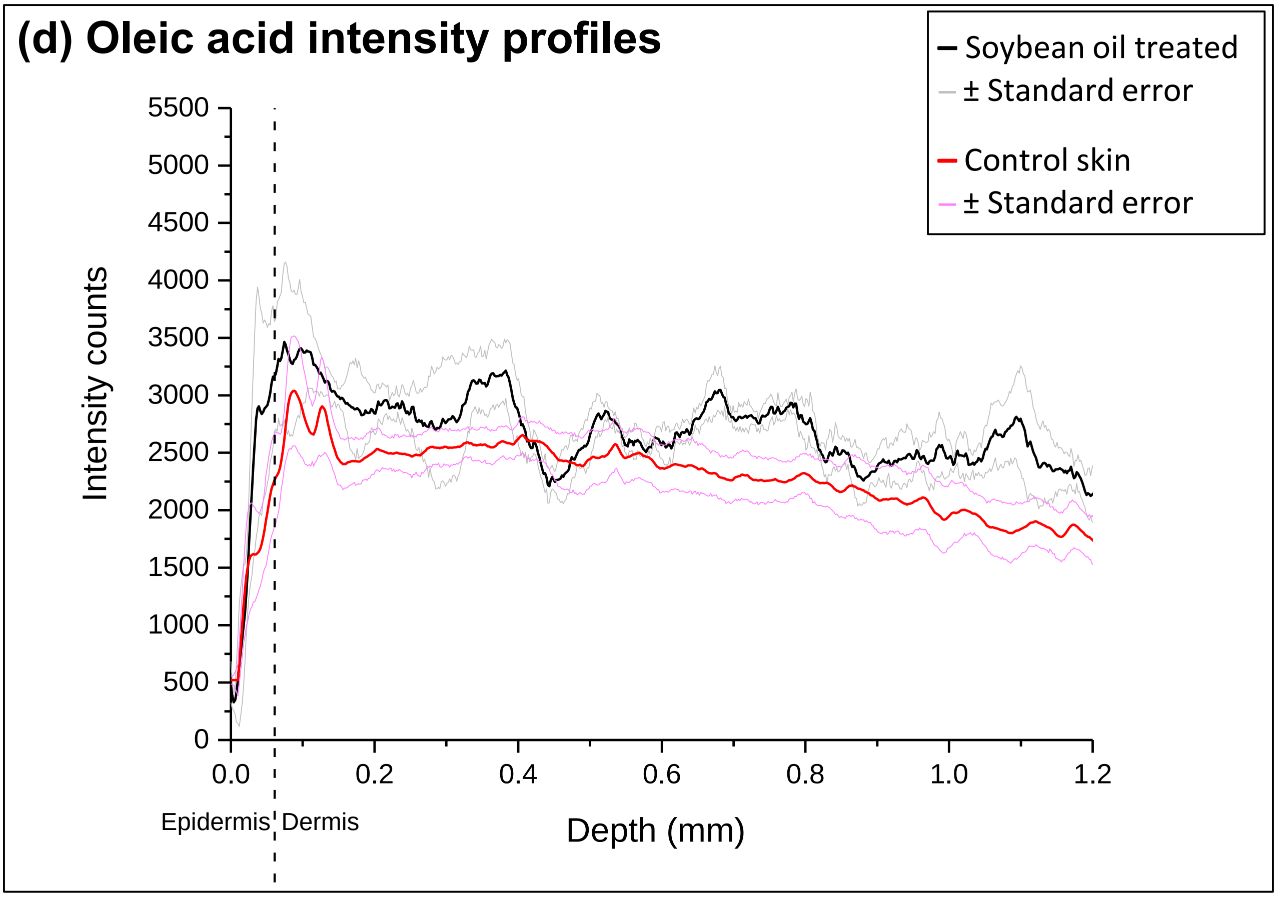

Oleic acid ion intensity profile as the function of depth in soybean oil treated skin samples and compared to the profile obtained from the control skin samples.

Black line corresponds to the average ion intensity values obtained from integrated ion images of skin samples treated with soybean oil; red line corresponds to the profile obtained in control skin samples.

Stearic acid intensity profiles

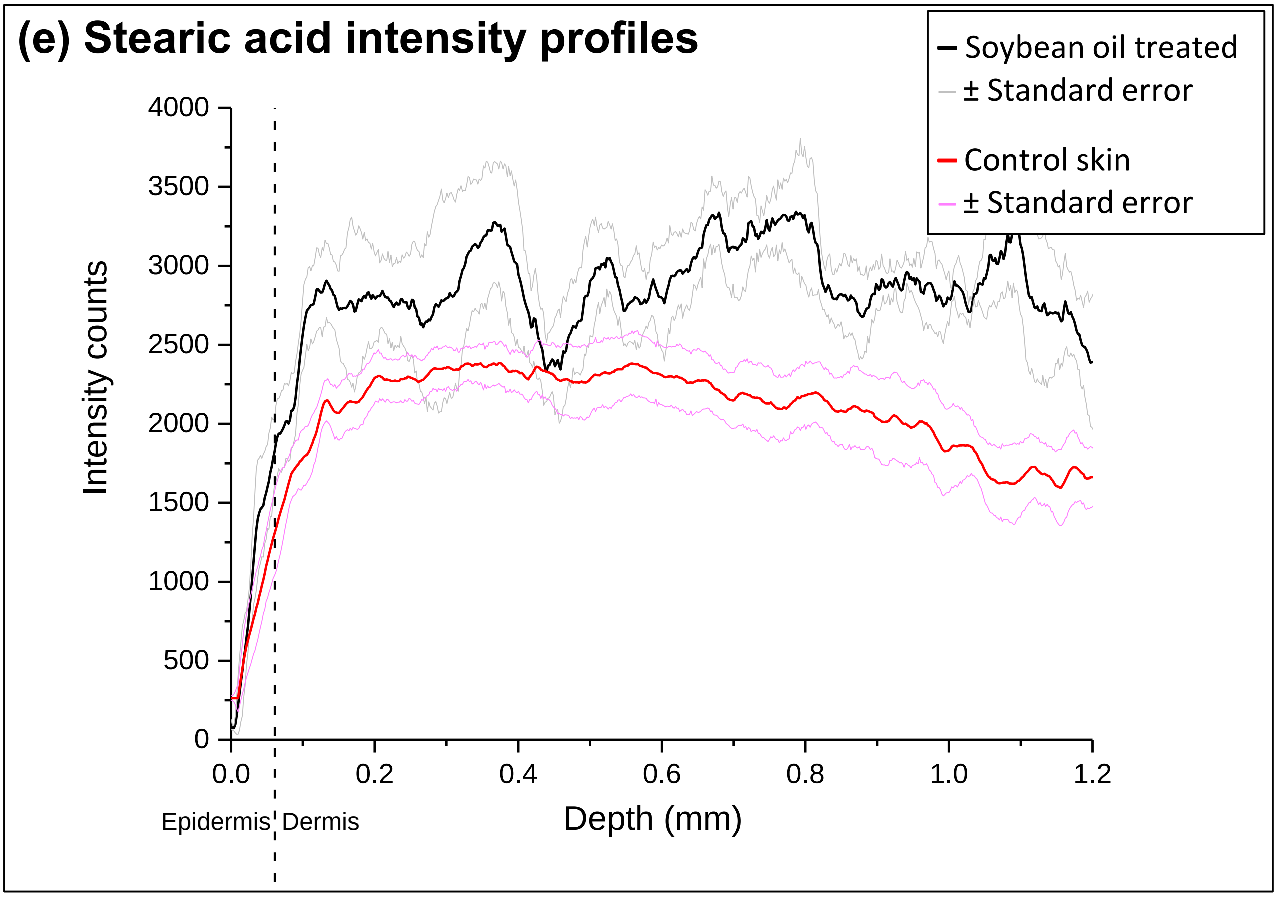

Stearic acid ion intensity profile as the function of depth in soybean oil treated skin samples and compared to the profile obtained from the control skin samples.

Black line corresponds to the average ion intensity values obtained from integrated ion images of skin samples treated with soybean oil; red line corresponds to the profile obtained in control skin samples.

Semi-quantitative changes of fatty acid content in the samples treated with natural oils

Fatty acid content changes in the skin layers after application of natural oils

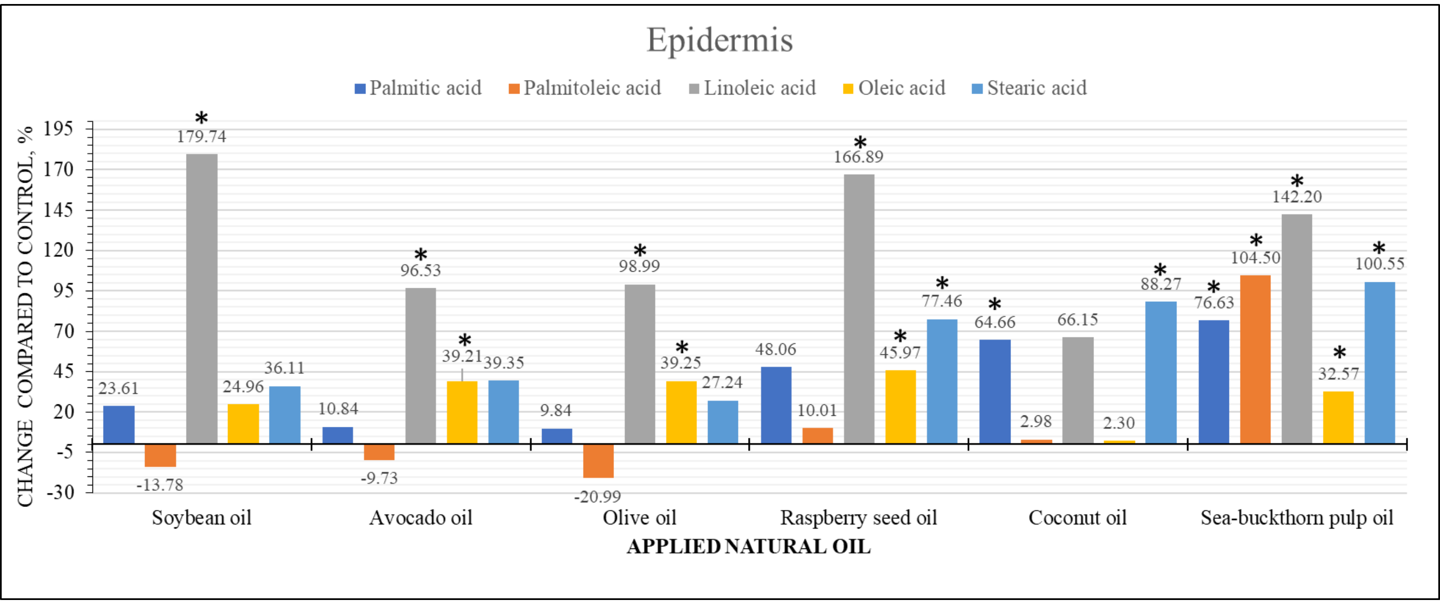

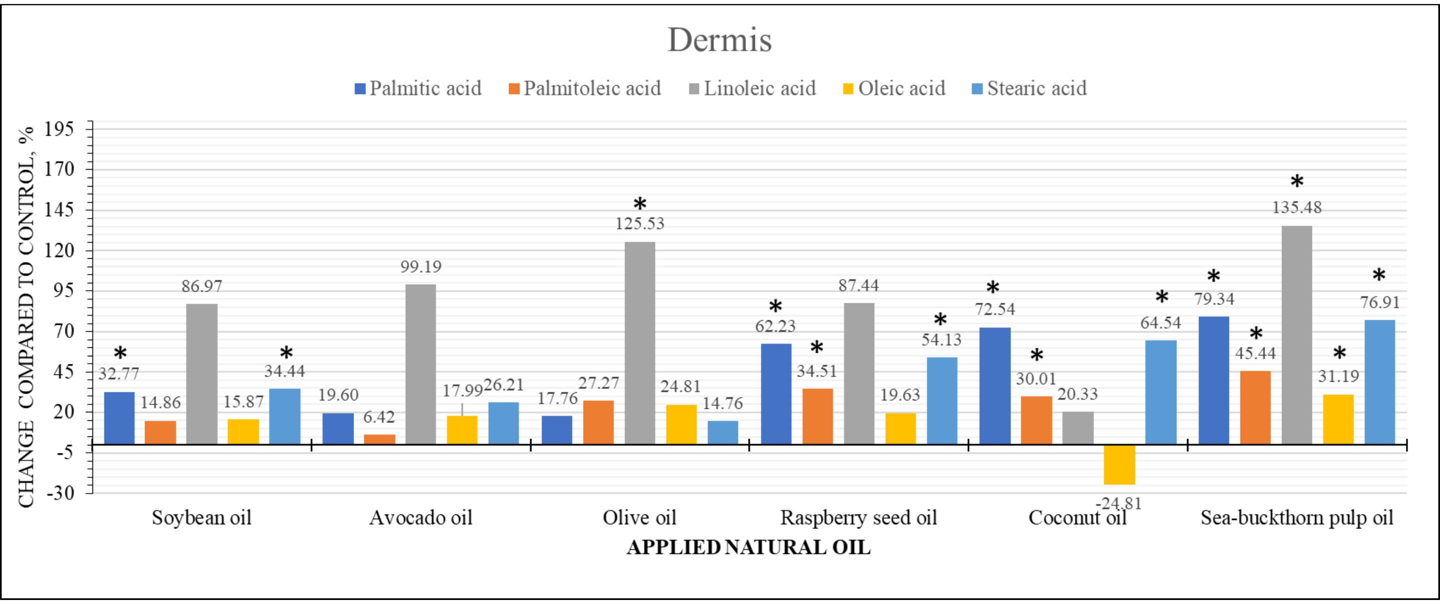

Epidermis thickness measurements of the skin samples

Sample treatment	Epidermis; Length [μm]				
	Thickness 1	Thickness 2	Thickness 3	Thickness 4	Thickness 5
Control skin 1	82.29	49.35	44.80	75.23	59.80
Control skin 2	36.12	60.23	45.17	35.85	38.41
Control skin 3	75.36	65.53	87.43	55.98	49.06
Avocado 1	55.98	37.21	53.04	56.81	48.18
Avocado 2	56.61	81.10	58.74	65.26	64.80
Avocado 3	39.81	78.17	43.00	52.54	50.33
Olive oil 1	50.30	66.56	62.29	70.99	64.00
Olive oil 2	59.10	55.05	56.61	79.07	61.60
Olive oil 3	32.81	81.78	50.07	53.35	59.61
Soybean oil 1	87.49	116.59	73.67	79.16	129.47
Soybean oil 2	76.80	70.47	39.74	55.24	48.00
Soybean oil 3	99.97	70.30	77.44	75.62	77.54
Sea-buckthorn pulp oil 1	80.83	45.60	37.12	44.84	61.49
Sea-buckthorn pulp oil 2	56.33	62.08	59.64	47.35	48.34
Sea-buckthorn pulp oil 3	82.91	74.94	68.03	59.56	48.79
Raspberry seed oil 1	74.42	61.55	58.88	74.22	40.50
Raspberry seed oil 2	46.24	83.35	119.99	41.04	67.50
Raspberry seed oil 3	52.09	70.18	45.82	87.87	37.72
Coconut 1	61.64	64.46	46.24	65.17	50.96
Coconut 2	46.76	50.56	46.76	50.71	53.10
Coconut 3	48.00	57.69	68.93	92.14	45.25
			**Total average:**		**61.62**
			**Standard deviation:**		**17.81**
